# Associations between five forms of child maltreatment and depression: a multilevel meta-analytic comparison

**DOI:** 10.1017/S003329172610381X

**Published:** 2026-04-06

**Authors:** Bruce Rind, Gerulf Rieger

**Affiliations:** 1 Independent Researcher; 2 Department of Psychology, Webster Vienna Private University, Vienna, Austria

**Keywords:** abuse and neglect, child maltreatment, depression, multilevel meta-analysis

## Abstract

**Background:**

Child maltreatment is strongly linked to depression, yet comparisons across maltreatment forms have been inconsistent. Prior meta-analyses mostly used single-level models and combined studies assessing different subsets of maltreatment forms, introducing statistical dependence and between-samples confounds that can distort cross-form comparisons.

**Methods:**

We synthesized data from 12 eligible meta-analytic reviews (those assessing at least emotional, physical, and sexual abuse, and providing effect size data), extracting 563 effect sizes from 217 depression risk studies and 501 effect sizes from 157 depression severity studies. Meta-analyses used two-level random-effects multilevel models, accounting for within-study dependence. Initial analyses compared all abuse forms plus emotional and physical neglect. Subsequent analyses compared just abuse forms either from samples assessing all three (‘complete-abuse’ samples) or only one or two (‘incomplete-abuse’ samples), which addressed between-samples confounds.

**Results:**

Effect sizes for different maltreatment forms were strongly correlated within studies (median *r*s ≈ .46–.48), confirming statistical dependence. Across all analytic layers, emotional abuse showed the strongest association with depression, and sexual abuse the weakest. In complete-abuse studies – the most internally comparable designs – a clear hierarchy emerged: emotional abuse > physical abuse > sexual abuse for both risk and severity. Incomplete-abuse studies obscured these differences.

**Conclusions:**

By modeling effect size dependence and reducing between-samples confounds, this study provides clearer evidence that emotional maltreatment – particularly emotional abuse – is most strongly linked to depression. These findings underscore the need for greater clinical and prevention focus on emotional forms of maltreatment.

## Introduction

Adverse childhood experiences are related to long-term psychological, social, and physical health difficulties (Felitti et al., 1998). Among these experiences, child maltreatment is commonly categorized as emotional, physical, and sexual abuse, and emotional and physical neglect (Norman et al., 2012; Wang, Chen, Zhou, & Zhang, 2023). Depression is among the most frequently examined outcomes of maltreatment, given its prevalence and global disease burden (Murray et al., 2012; World Health Organization, 2017).

Historically, research has emphasized sexual abuse, reflecting assumptions about its comparatively severe psychological consequences (Gardner, Thomas, & Erskine, 2019; Infurna et al., 2016). Accordingly, early meta-analyses focused largely or exclusively on sexual abuse (e.g. Jumper, 1995; Neumann, Houskamp, Pollock, & Briere, 1996). As research expanded, meta-analyses began addressing additional maltreatment forms, and more recent reviews have included multiple forms within the same analytic framework to allow cross-form comparisons (e.g. Li, D’Arcy, & Meng, 2016; Norman et al., 2012).

In the past decade, a series of meta-analyses has synthesized associations between depression and emotional, physical, and sexual abuse, and – less consistently – emotional and physical neglect (e.g. Gardner et al., 2019; Humphreys et al., 2020; Mandelli, Petrelli, & Serretti, 2015). These reviews have advanced comparative understanding, and their key characteristics are summarized in Table 1.

**Table 1.** Previous meta-analyses of child maltreatment (assessing emotional, physical, and sexual abuse at the minimum) versus depression, from 2015 to 2024

Meta-analysis	Key INTRO remarks	Samples included	*k*	CM (*k* samples), effect size (95% CI)	Moderator analyses
Mandelli et al. (2015)	Emphasized EA as ‘silent’ form that may have large effects; need to clarify contribution of separate forms to depression	Adults (≥18); with CMs or ACEs and depression (interviews or self-reports) assessed	26	Effect size = OR: EA (8) = 2.78 (1.89, 4.09) PA (10) = 1.98 (1.68, 2.33) SA (14) = 2.42 (1.94, 3.02) *N* (6) = 2.75 (1.59, 4.74)	SA effect size lower in clinical than community samples; N highest in clinical, EA highest in community samples
Infurna et al. (2016)	Most studies on SA and PA, but EA needs more recognition; separate estimates needed; predicted EA would be more potent	Studies using the Childhood Experience of Care and Abuse interview (CECA) to assess CMs; *k* = 8 based on adults, *k* = 4 on adolescents	12	Effect size = *d*: antip (5) = .52 (20, .83) psy ab (2) = .93 (.93, .93) PA (6) = .81 (.69, .93) SA (6) = .50 (.22, .78) *N* (6) = .81 (.61, 1.02) *(EA = antipathy, psychological abuse)*	Adolescent participants had lower effect size than adults for antipathy, equal for other CMs
Nelson et al. (2017) (1)	Prior research showed different effects by CM form on depression, with EA and EN most potent; need to study CMs separately	Adults; any CM; depression as diagnosis or symptom severity	83	Effect size = OR: EA (15) = 3.73 (2.88, 4.83) PA (38) = 2.68 (2.29, 3.12) SA (57) = 2.66 (2.38, 2.98) EN (9) = 3.54 (2.48, 5.04) PN (7) = 2.45 (1.63, 3.68)	Only 1/23 metaregressions was significant; in subgroup analyses, no difference between CTQ versus other measures of CM; in clinical versus other, only 1 difference (for PN)
Nelson et al. (2017) (2)	Test dose–response of CMs on depression via correlations (both measured on continuum)	(see before)	52	Effect size = *r*: EA (24) = .29 (0.25, 0.33) PA (27) = .20 (0.16, 0.24) SA (30) = .17 (0.12, 0.21) EN (15) = .26 (0.20, 0.32) PN (12) = .20 (0.15, 0.25)	(see before)
Yu, Zhao, and Liu (2017)	Aim was to examine multiple CMs in relation to depression; prior research mostly did not do this	Sought studies from English and Chinese databases assessing CM in relation to depression	31	Effect size = OR: EA (19) = 3.58 (2.87, 4.48) PA (20) = 2.30 (1.84, 2.86) SA (17) = 2.46 (1.94, 3.12) EN (12) = 3.24 (2.43, 4.32) PN (8) = 2.90 (2.06, 4.07)	Adolescents had a higher effect size than adults; females had higher than males
Gardner et al. (2019)	Only SA included in Global Burden of Disease Study; assessing other CMs may help add information	Used population-representative samples (no clinical or convenience)	106	Effect size = OR: EA (14) = 2.35 (1.74, 3.18) PA (41) = 1.78 (1.57, 2.01) SA (46) = 2.11 (1.83, 2.44) N (15) = 1.65 (1.35, 2.02)	Stratification did not reveal any significant effects and did not reduce heterogeneity
Humphreys et al. (2020) (1)	SA has received the most attention, but CMs frequently co-occur; so examine all forms; use a single measure of CM (the CTQ) to reduce variability	Studies included that used CTQ and assessed depression as diagnosis	39	Effect size = *g*: EA (35) = .85 (.77, .94) PA (35) = .47 (.37, .57) SA (35) = .44 (.36, .53) EN (35) = .96 (.85, 1.08) PN (35) = .65 (.53, .78)	EA had larger effect size for under 18 age; *g* was larger in English version of CTQ for SA, EN
Humphreys et al. (2020) (2)	(see before)	Studies included that used CTQ and assessed depression as severity of symptoms	92	Effect size = *zr*: EA (81) = .38 (.34, .41) PA (66) = .22 (.18, .25) SA (72) = .20 (.17, .23) EN (58) = .30 (.26, .34) PN (48) = .23 (.20, .27)	*zr* was less in population versus other samples for EN, PN, and EA;
LeMoult et al. (2020)	Different types of early life stress (ELS) likely create different risks for depression. So, study each separately in adolescent-onset	Diagnostic major depressive disorder (MDD) assessment prior to mean age of 18, with control group	57	Effect size = OR: EA (4) = 2.97 (1.51, 5.82) PA (13) = 2.13 (1.61, 2.81) SA (19) = 1.62 (1.26, 2.06)	For SA, source (volunteer, community > clinical) affected effect size, but not method of assessing ELS, in model including both moderators
Kuzminskaite et al. (2022) *^a^*	Compared depression in depressed patients with versus without CM, at baseline and after treatment	MDD patients in pharmacotherapy, psychotherapy, or both in randomized clinical or open trials to test effectiveness of therapies	54	Effect size = *g*: EA (50) = .31 (.22, .39) PA (52) = .34 (.25, .44) SA (53) = .29 (.21, .38) EN (45) = .20 (.13, .26) PN (45) = .27 (.18, .35) *(baseline values)*	Patients with CM benefited from active treatment similarly to patients without CM, equally across CM forms
Lai et al. (2023)	Used multilevel models to handle dependencies of effect sizes; used single measure of CM (CTQ); studied cultural influences on correlations	Chinese samples exclusively, including case–control, clinical, student, convenience	24	Effect size = *r*: *^b^* EA (23) = .29 (.25, .34) PA (24) = .15 (.11, .20) SA (23) = .14 (.08, .19) EN (24) = .24 (.21, .30) PN (23) = .23 (.18, .27)	SA-depression correlations stronger in Southern than Central China; attributed this to cultural norms (the south is much more Westernized)
Li et al. (2023)	Goal: to see if age at exposure to CM (developmental stage) affects depression risk	All samples (clinical, student, volunteer, shelters, forensic) and ages (children, adolescents, adults); had to have age of exposure	25	Effect size = *r*: *^b^* EA (21) = .17 (.11, .23) PA (29) = .13 (.10, .15) SA (41) = .18 (.15, .21) N (11) = .08 (.06, .11)	Highest effect size in age group 6–13 in CM exposure; then 12–19; least was 0–6
Souama et al. (2023) *^a^*	Compare effect sizes of depression, cardiometabolic disease, and comorbidity versus risks on CMs	Samples assessing these outcomes and PA, EA at minimum; result was 10 population-based cohort studies, 3 case–control studies	13	Effect size = OR: EA (6) = 2.88 (2.51, 3.31) PA (6) = 1.64 (1.49, 1.81) SA (6) = 1.66 (1.55, 1.78) *(depression-only and comorbid combined)*	PA and EA, but not SA, were ‘particularly strong predictors of comorbidity’
Tan and Mao (2023)	Abuse and neglect have received the most attention in meta-analyses; but other, ‘silent’, forms need attention, too	All published studies in English using ACE-IQ and assessing depression	9	Effect size = OR: EA (6) = 2.48 (1.97, 3.00) PA (6) = 1.71 (1.05, 2.37) SA (6) = 1.78 (1.41, 2.15) EN (3) = 1.94 (0.65, 3.23) PN (3) = 2.11 (1.08, 3.14)	ACE total scores versus depression tended higher in Africa than Asia (ORs = 3.14 versus 2.58) and residents than college students (ORs = 3.70 versus 2.13)
Wang et al. (2023)	20% of adults over 60 worldwide suffer from late-life depression; aim to study all forms of maltreatment	Searched Chinese and English databases; mean age of participants had to be >60 years	10	Effect size = OR: EA (5) = 1.92 (1.73, 2.12) PA (9) = 1.73 (1.55, 1.95) SA (5) = 1.46 (0.91, 2.32) EN (6) = 3.25 (1.43, 7.39) PN (4) = 2.05 (1.15, 3.67)	Found some differences, but based on too-small number of samples
Yu et al. (2024)	Focus was 9 early-life adversities versus child- or adolescent-onset depression	Mean sample age < 18; sample distribution was broad: Asia (44%); US/Can (31%); Europe (13%)	87	Effect size = OR: *^c^* EA (18) = 4.25 (3.04, 5.94) PA (23) = 2.21 (1.88, 2.59) SA (25) = 1.85 (1.57, 2.19) EN (7) = 3.09 (1.75, 5.17) PN (5) = 2.25 (2.00, 2.52)	Moderators were almost entirely unrelated to effect sizes across all forms of child maltreatment

*Note*: CM, child maltreatment; EA, emotional abuse; EN, emotional neglect; PA, physical abuse; PN, physical neglect; SA, sexual abuse; N, neglect (both EN and PN); OR, odds ratio; *d*, Cohen’s *d*; *g*, Hedge’s *g*; *r*, standardized correlation coefficient; *zr*, Fisher’s *z* transformation from *r.*

^a^ *In Kuzminskaite et al. (2022) and Souama et al. (2023), individual-study effect sizes for CM forms were not provided in the publications or online, so data from them were not included in the present meta-analyses.*

^b^ *A quarter to a half the r-effect sizes in Lai et al. (2023) and Li et al. (2023) were point-biserial and were converted to ORs for analysis; the remaining were Pearson’s correlations, entered into the severity analyses.*

^c^ *Some of the ORs in Yu et al. (2024) were identified as based on Pearson’s correlations; these were entered into the severity analyses instead.*

### Key limitations of previous meta-analyses

Most prior meta-analyses of maltreatment versus symptoms have used single-level models and treated effect sizes from different maltreatment forms as independent – the case for almost all the reviews in Table 1 (exceptions were Lai et al., 2023, who employed multilevel models, and Nelson et al., 2017, who used single-level models but employed robust variance estimation to address dependencies). However, primary studies have frequently reported multiple effect sizes for several maltreatment forms, making those estimates statistically dependent. Ignoring this dependence can bias pooled estimates and invalidate cross-form comparisons (Assink & Wibbelink, 2016; Cheung, 2014; van den Noortgate, López-López, Marín-Martínez, & Sánchez-Meca, 2013).

A second limitation concerns between-samples confounds. Many prior reviews included large numbers of studies that assessed only one or two maltreatment forms. If samples contributing effect sizes for different maltreatment forms differ systematically – for example, if researchers studying sexual abuse versus physical abuse disproportionately recruit different populations – then comparisons of pooled effects may conflate true differences with sample composition. More comparable estimates arise from ‘complete-form’ studies assessing most or all maltreatment forms, because sample characteristics are held constant across those forms.

### Emerging pattern

Despite these limitations, an overall pattern emerges across existing meta-analyses: emotional abuse and emotional neglect often show the strongest associations with depression, whereas sexual abuse tends to yield comparatively smaller effects (e.g. Humphreys et al., 2020; Infurna et al., 2016; Nelson, Klumparendt, Doebler, & Ehring, 2017; Yu, Cao, Shang, & Li, 2024). These findings suggest a central role for emotional maltreatment, though the methodological issues noted earlier temper the strength of this inference.

### The present study

We employed effect size data provided by the source meta-analyses (Table 1), eliminating duplicates. First, we compared all five child maltreatment forms (emotional, physical, and sexual abuse; emotional and physical neglect) in relation to depression. To account for statistical dependence within samples, we employed multilevel random-effects meta-analyses, with Level 1 representing sampling variance among effect sizes and Level 2 modeling the clustering of multiple effect sizes within studies (Cheung, 2014; Konstantopoulos, 2011). We performed two types of meta-analyses: *risk* (i.e. depression as a binary outcome, based on diagnoses—e.g. Mandelli et al., 2015—or dichotomized symptom scores—e.g. Gardner et al., 2019) and *severity* (i.e. depression and maltreatment as continuums). Second, focusing just on abuse, we meta-analyzed effect sizes (risk and severity) from ‘complete-abuse’ samples (assessing all three forms of abuse) and then ‘incomplete-abuse’ samples (assessing only one or two abuse forms) to address the issue of between-samples confounds.

Using these approaches, we obtained (a) pooled associations between each maltreatment form and depression; (b) direct pairwise comparisons among the forms; and (c) estimates of heterogeneity (*Q*, *I* ^2^) within the multilevel framework. This design along with the huge set of samples and effect sizes offered a methodologically more rigorous comparison of how different maltreatment experiences relate to depression than previous meta-analytic reviews.

Based on the pattern emerging from prior reviews (Table 1), we hypothesized that emotional abuse would exhibit the strongest association with depression, whereas sexual abuse would show substantially weaker associations. We further expected that this hierarchical pattern among abuse forms would emerge most clearly in analyses restricted to complete-abuse samples. In contrast, we anticipated greater inconsistency in analyses based on incomplete-abuse samples, which assessed only one or two abuse forms and therefore confounded comparisons with between-samples differences. Across all analyses, our overarching goal was to obtain valid comparative estimates and establish a credible ranking of maltreatment forms in their associations with depression, based on wide-ranging samples geographically (e.g. from the U.S. to China) and compositionally (e.g. from clinical to representative).

## Method

### Search and inclusion criteria

We conducted a systematic search for published meta-analyses examining associations between child maltreatment and depression. Eligibility was restricted to reviews that reported separate effect size estimates for at least three forms of maltreatment – emotional, physical, and sexual abuse. This criterion ensured provision of comparative estimates of key maltreatment forms and increased the likelihood that source studies included multiple maltreatment forms.

Searches were conducted on January 30, 2025, using Google Scholar and SciSpace. For Google Scholar, the following terms were entered: *meta-analysis*, *child maltreatment*, and *depression.* For SciSpace, the following query was entered: ‘Give a list of meta-analyses of child maltreatment and depression’. Two researchers with meta-analytic experience (the first author and an independent coder) independently screened the outputs. Because Google Scholar ranks results by relevance rather than enforcing strict term matching, screening on that platform was limited to the first 100 results, after which retrieved records were largely irrelevant to the research question. Reviews were retained for further consideration if their titles included the terms *meta-analysis* and either *depression* or *depressive*, along with *child maltreatment* or a related term indicating exposure to adverse experiences in childhood (e.g. *abuse*, *adverse childhood experiences*, *trauma*).

Abstracts of selected reviews were then examined. Following discussion and consensus, full texts were obtained for reviews, in which provision of separate effect size estimates for emotional, physical, and sexual abuse in relation to depression was considered possible. No restriction was placed on publication year.

Full texts were independently evaluated to determine whether they reported separate meta-analytic effect size estimates for at least emotional, physical, and sexual abuse. Intercoder agreement was complete. Reviews meeting this criterion were retained for analysis. Reference lists of included reviews were also examined to identify additional eligible meta-analyses.

**Figure 1.**
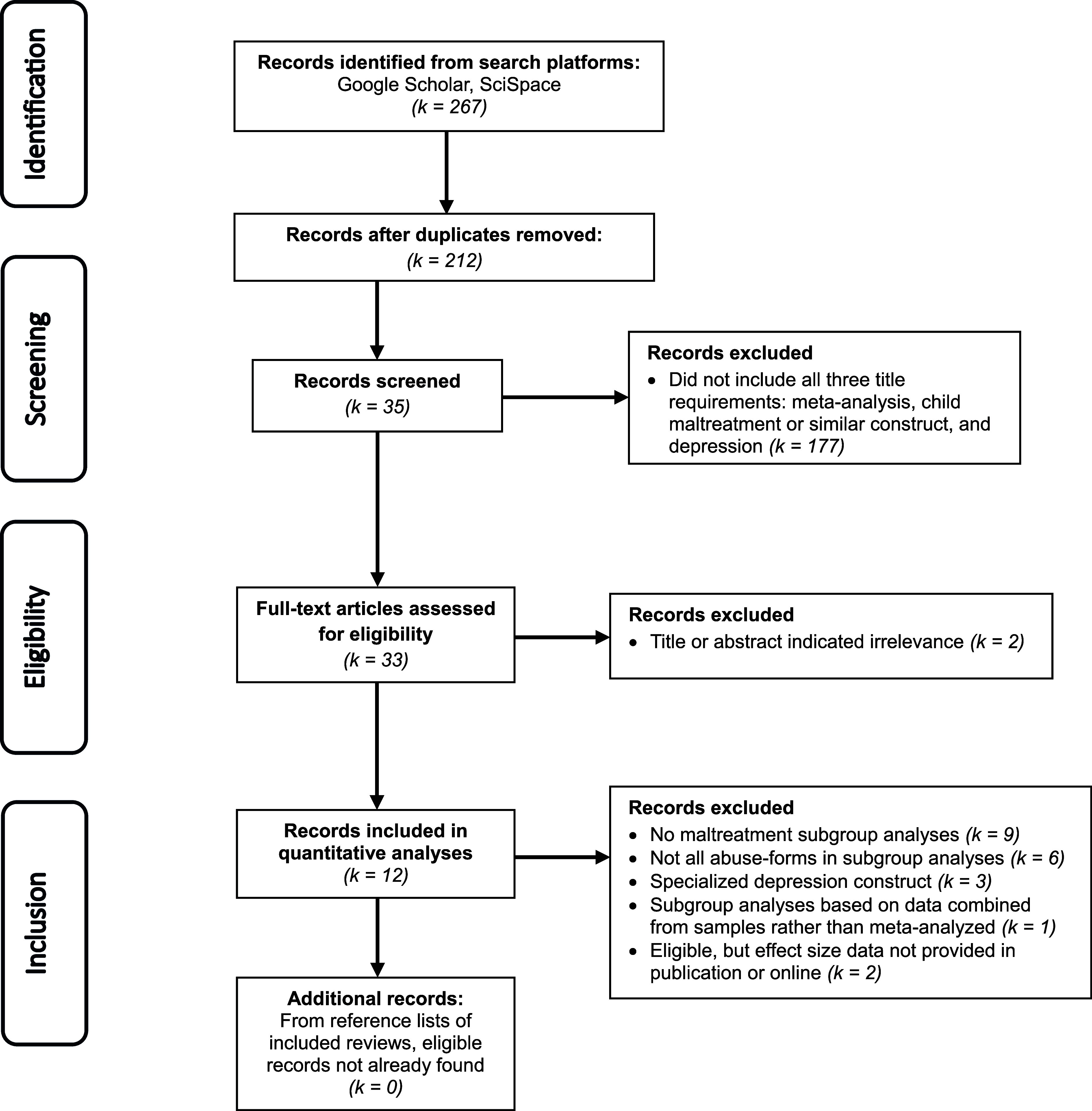
Flowchart of included reviews.

Ten reviews, published between 2015 and 2023 and comprising 12 meta-analyses, met the inclusion criteria. As a recheck and final verification step, the search was repeated on January 20, 2026, using identical search terms, to determine whether additional eligible reviews had become listed or were originally missed. Two were found and added (Lai, Fan, Man, & Huang, 2023; Yu et al., 2024), yielding a final sample of 12 reviews encompassing 14 meta-analyses, which were used to extract effect size data for the present meta-analyses (Table 1). Two additional reviews deemed eligible (Kuzminskaite et al., 2022; Souama et al., 2023) did not provide effect size data in their publication or online; hence, their data did not enter the present meta-analyses (for completeness, their results are also included in Table 1). A flowchart summarizing this updated search and selection process is provided in Figure 1.

Although this study is not a primary-level systematic review and therefore did not implement a full PRISMA protocol, the meta-analytic reviews from which effect sizes were drawn almost uniformly reported PRISMA-compliant searches, screenings, and reporting procedures, sometimes with preregistered protocols. In addition, several of these reviews formally evaluated the quality or risk of bias of original studies using established tools (e.g. the Newcastle–Ottawa Scale, NIH Quality Assessment Tool, or structured quality checklists), generally finding that most included studies met moderate to good quality thresholds (e.g. LeMoult et al., 2020; Li et al., 2023; Mandelli et al., 2015; Tan & Mao, 2023; Wang et al., 2023; Yu et al., 2024). Where examined, study quality did not significantly moderate effect size estimates, and sensitivity analyses excluding lower quality studies yielded comparable results (e.g. LeMoult et al., 2020; Mandelli et al., 2015). Other reviews suggested that publication bias, when present, had only modest influence on pooled estimates (e.g. Humphreys et al., 2020; Nelson et al., 2017). Thus, while this review did not independently assess study quality, it drew on an evidence base that was systematically evaluated for methodological rigor and potential sources of bias, providing relevant context for interpreting the findings summarized here.

### Definitions of child maltreatment

This research examined emotional, physical, and sexual abuse, plus, if assessed in the reviewed research, emotional and physical neglect. Definitions of the child maltreatment forms here reflect their definitions in the included studies, which were based on measures such as the Childhood Trauma Questionnaire (Bernstein & Fink, 1998), the Adverse Childhood Experiences Questionnaire (Felitti et al., 1998), and the Maltreatment and Abuse Chronology of Exposure Scale (Teicher & Parigger, 2015). Definitions varied across measures and studies, but their conceptual cores can be outlined as follows.

Emotional abuse referred to repeated acts of verbal hostility, rejection, humiliation, or threats by caregivers or adults, which undermine a child’s emotional development and self-worth (Teicher, Samson, Polcari, & McGreenery, 2006). Examples include name-calling, belittling, and making the child feel unwanted.

Emotional neglect involved persistent failure to meet a child’s basic needs for affection, support, and belonging (Glaser, 2002). Examples include a lack of warmth shown to the minor or a lack of comfort provided during distress.

Physical abuse denoted intentional use of physical force that results in, or potentially results in, injury (Gilbert et al., 2009). Examples are hitting, kicking, slapping, or burning.

Physical neglect referred to chronic failure to provide for a child’s basic physical needs, including failure to provide food, clothing, hygiene, medical care, and protection (Dubowitz, Pitts, & Black, 2004).

Definitions for sexual abuse were more varied than those for other maltreatment forms (e.g. in types of acts, ages involved). In general, they focused on unwanted, negative sexual experiences before age 18 with other minors or adults (Bernstein & Fink, 1998; World Health Organization, 2020). Less often, they were based solely on age-discrepancies, for instance, sexual experiences before age 18 with someone at least 5 years older, including willing encounters (Wyatt, 1985).

### Data extraction

Effect size extraction was performed by the first author. Effect sizes were obtained directly from the included meta-analytic reviews (Table 1), taken from tables or figures in the publications or online, when provided (as noted previously, two reviews did not provide these data). When information in a review was unclear or missing, effect sizes were extracted from the source studies.

For the risk analyses, odds ratios, Cohen’s *d*s, and Hedges’ *g*s (with 95% confidence intervals) were converted to log odds with corresponding standard errors (Cooper, Hedges, & Valentine, 2019). Point–biserial correlations (in Lai et al., 2023 and Li et al., 2023) were likewise converted to log odds with standard errors. For the severity analyses, in which both maltreatment and depression were assessed using continuous scales, Pearson’s correlations (with 95% confidence intervals) were converted to Fisher’s *z*s with standard errors, and reported Fisher’s *z*s (in Humphreys et al., 2020) were entered directly, with confidence intervals converted to standard errors.

Duplicates of source studies across reviewed meta-analyses were removed (if their reported effect sizes differed, effect sizes were extracted from the source studies). When a source study reported multiple effect sizes for the same maltreatment form (e.g. different age groups), effect sizes were aggregated following standard procedures (Borenstein, Hedges, Higgins, & Rothstein, 2009) to avoid violating independence. However, effect sizes were kept separate by sex, consistent with practices in the reviewed meta-analyses.

Finally, information on moderating variables was extracted by both coders from information provided in the reviews, or from each source study when necessary, including publication year, maltreatment measure, sample type (case–control, representative, clinical, convenience), sample size, percentage of female participants, country or region, and language of assessment.

### Statistical analyses

Two-level random-effects multilevel meta-analyses were conducted in JASP (JASP Team, 2025). Level 1 represented the sampling variance of effect sizes, whereas Level 2 modeled the clustering of multiple effect sizes within studies, thereby accounting for their statistical dependence (Cheung, 2014; Konstantopoulos, 2011). When dependencies are addressed directly through an appropriate multilevel structure, additional corrections such as robust variance estimation are generally unnecessary and may yield overly conservative standard errors (Borenstein, Hedges, Higgins, & Rothstein, 2021; Cheung, 2014; Hedges, Tipton, & Johnson, 2010).

Restricted maximum likelihood (REML) estimation was used. Residual heterogeneity was evaluated using the *Q* statistic, which tests whether the observed variability in effect sizes exceeds what would be expected from sampling error alone after accounting for the predictors in the model. The *I* ^2^ statistic quantified the proportion of total observed variance attributable to true heterogeneity rather than sampling error. Moderator analyses were conducted using metaregression within the multilevel framework.

Pairwise comparisons among maltreatment forms were conducted using iterative reference-category coding. With five maltreatment forms (three abuse, two neglect), this produced 10 planned pairwise contrasts, requiring a Bonferroni-adjusted familywise significance level of *α* = .005. A separate set of contrasts examined only the three abuse forms, yielding three pairwise comparisons and a Bonferroni-adjusted *α* = .017. As noted earlier, risk (binary outcome; analyzed using log odds) and severity (continuous outcome; analyzed using Fisher *z*) were examined in parallel models. For interpretability, log-odds estimates were converted to odds ratios, and Fisher’s *z* values were converted to Pearson’s *r*s.

## Results

### Effect size dependencies

We first examined the extent to which effect sizes for different maltreatment forms were statistically dependent within samples. Because complete-abuse studies (i.e. those assessing all three abuse forms) provided the fullest information for pairwise comparisons, we restricted this analysis to those samples. Log-odds ratios (risk) and Fisher’s *z* (severity) correlations were examined separately.

Effect sizes were strongly correlated across maltreatment forms (Table S1 in the Supplementary Material). For risk, all pairwise correlations were significant, with a median *r* = .46. For severity, all but one were significant, with a median *r* = .48. These substantial cross-form correlations confirm that effect sizes were not independent within studies, supporting our use of multilevel modeling to account for statistical dependence.

### Multilevel meta-analyses: all samples and maltreatment forms

Our initial multilevel meta-analyses drew on all effect sizes extracted from the 12 eligible meta-analytic reviews. Although all reviews assessed at least the three abuse forms, individual primary studies varied: some assessed all five maltreatment forms, whereas many assessed only one or two.

#### Depression risk

Table 2 summarizes results for depression risk (225 samples, 217 studies; 563 effect sizes; *N* = 379,383). Emotional abuse showed the strongest association with depression (OR = 3.64, 95% CI [3.38, 3.92]). Sexual abuse showed the weakest association (OR = 2.06, 95% CI [1.92, 2.22]). Physical abuse and physical neglect (ORs = 2.74 and 2.67, respectively) exceeded sexual abuse, whereas emotional neglect was intermediate (OR = 2.40). Substantial residual heterogeneity was observed, *Q*(558) = 6844.18, *p* < .001, *I* ^2^ = 91.8%.

**Table 2.** Meta-analysis (multilevel, random-effects, all samples) of risk of depression in relation to five forms of child maltreatment, employing samples used in meta-analyses from 2015 to 2024

			95% CI		
Child maltreatment	*k*	OR	LL	UL	Post hoc *^a^*
Emotional abuse	99	3.64	3.38	3.92	a
Emotional neglect	61	2.40	2.23	2.58	c
Physical abuse	158	2.74	2.55	2.94	b
Physical neglect	58	2.67	2.47	2.88	b
Sexual abuse	187	2.06	1.92	2.22	d

*k, number of samples for a given maltreatment; OR, odds ratio risk of depression; Q(558) = 6844.18, p < .001; I^2^ = 91.8%.*

^a^ *Maltreatments without common letters (in last column) were significantly different at Bonferroni-adjusted alpha level of .005.*

#### Depression severity

Results for severity (162 samples, 157 studies; 501 effect sizes; *N* = 87,659) appear in Table 3. Emotional abuse again showed the strongest association (*r* = .35, 95% CI [.33, .37]), followed by emotional neglect (*r* = .31, 95% CI [.29, .33]). Sexual abuse was least associated (*r* = .18, 95% CI [.17, .20]). Physical abuse and physical neglect were intermediate. Heterogeneity remained high, *Q*(496) = 4068.66, *p* < .001, *I* ^2^ = 87.8%.

**Table 3.** Meta-analysis (multilevel, random-effects, all samples) of severity of child maltreatment versus severity of depression, employing samples used in meta-analyses from 2015 to 2024

			95% CI		
Child maltreatment	*k*	*r*	LL	UL	Post hoc *^a^*
Emotional abuse	123	0.35	0.33	0.37	a
Emotional neglect	81	0.31	0.29	0.33	b
Physical abuse	110	0.22	0.20	0.24	c
Physical neglect	67	0.23	0.21	0.25	c
Sexual abuse	120	0.18	0.17	0.20	d

*k, number of samples for a given maltreatment; r, Pearson’s correlation representing magnitude of association between severity of maltreatment and severity of depression; Q (496) = 4068.66, p < .001; I^2^ = 87.8%.*

^a^ *Maltreatments without common letters (in last column) were significantly different at Bonferroni-adjusted alpha level of .005.*

### Complete-abuse versus incomplete-abuse samples

We next examined whether the pattern of associations depended on whether studies assessed all three abuse forms (complete-abuse samples) or only one or two (incomplete-abuse samples). Neglect forms were excluded from this comparison because incomplete studies too infrequently assessed them, making parallel comparisons infeasible.

#### Depression risk

Results for complete-abuse samples (*k* = 81; effect sizes = 356; *N* = 71,054) appear in Table 4 (upper panel). A clear hierarchy emerged: emotional abuse produced the largest increase in risk (OR = 3.42, 95% CI [3.09, 3.78]), sexual abuse the smallest (OR = 1.85, 95% CI [1.68, 2.05]), with physical abuse intermediate (OR = 2.68, 95% CI [2.42, 2.96]). Heterogeneity was high, *Q*(240) = 2446.21, *p* < .001, *I* ^2^ = 90.2%.

**Table 4.** Meta-analysis (multilevel, random-effects) of risk of depression in relation to the three abuse forms of child maltreatment, conducted separately for complete- and incomplete-abuse samples, employing samples used in meta-analyses from 2015 to 2024

			95% CI		
	*k*	OR	LL	UL	Post hoc *^a^*
Complete-abuse samples					
Emotional abuse	81	3.42	3.09	3.78	a
Physical abuse	81	2.68	2.42	2.96	b
Sexual abuse	81	1.85	1.68	2.05	c
Incomplete-abuse samples					
Emotional abuse	18	3.09	2.67	3.59	a
Physical abuse	77	2.18	1.98	2.39	b
Sexual abuse	106	2.35	2.15	2.58	b

Complete-abuse samples assessed all 3 abuses (emotional, physical, sexual); incomplete-abuse samples did not. *k*, number of samples for a given maltreatment; OR, odds-ratio risk of depression. Complete-abuse: *Q*(240) = 2446.21, *p* < .001; *I* ^2^ = 90.2%. Incomplete-abuse: *Q*(198) = 1166.14, *p* < .001; *I* ^2^ = 83.0%.

^a^ *Maltreatments without common letters (in last column) were significantly different at Bonferroni-adjusted alpha level of .0167.*

For incomplete-abuse samples (*k* = 144; effect sizes = 207; *N* = 308,329), the pattern was attenuated (Table 4, lower panel). Emotional abuse reduced somewhat but retained the highest association (OR = 3.09), while sexual abuse increased (OR = 2.35), nominally exceeding physical abuse (OR = 2.18). Heterogeneity remained substantial, *Q*(198) = 1166.14, *p* < .001, *I* ^2^ = 83.0%.

#### Depression severity

Severity analyses for complete-abuse samples (*k* = 87; effect sizes = 386; *N* = 51,747) again produced a clear hierarchy (Table 5, upper panel): emotional abuse (*r* = .34) > physical abuse (*r* = .20) > sexual abuse (*r* = .17). Heterogeneity was high, *Q*(258) = 2021.76, *p* < .001, *I* ^2^ = 87.2%.

**Table 5.** Meta-analysis (multilevel, random-effects) of severity of depression in relation to severity of the three abuse forms of child maltreatment, conducted separately for complete- and incomplete-abuse samples, employing samples used in meta-analyses from 2015 to 2024

			95% CI		
	*k*	*r*	LL	UL	Post hoc *^a^*
Complete-abuse samples					
Emotional abuse	87	0.34	0.31	0.36	a
Physical abuse	87	0.20	0.18	0.22	b
Sexual abuse	87	0.17	0.14	0.19	c
Incomplete-abuse samples					
Emotional abuse	36	0.36	0.33	0.39	a
Physical abuse	23	0.23	0.19	0.26	b
Sexual abuse	33	0.23	0.19	0.26	b

Complete-abuse samples assessed all 3 abuses (emotional, physical, sexual); incomplete-abuse samples did not. *k*, number of samples for a given maltreatment; *r*, Pearson correlation representing magnitude of association between severity of maltreatment and severity of depression. Complete-abuse: *Q*(258) = 2021.76, *p* < .001; *I* ^2^ = 87.2%. Incomplete-abuse: *Q*(89) = 526.11, *p* < .001; *I* ^2^ = 83.1%.

^a^ *Maltreatments without common letters (in last column) were significantly different at Bonferroni-adjusted alpha level of .0167.*

Among incomplete-abuse samples (*k* = 75; effect sizes = 115; *N* = 35,912), emotional abuse remained significantly strongest (*r* = .36), but sexual abuse increased notably relative to the complete-abuse analysis (*r* = .23 vs .17), becoming equal to physical abuse (*r* = .23). Heterogeneity was substantial, *Q*(89) = 526.11, *p* < .001, *I* ^2^ = 83.1%.

### All maltreatment forms in complete-abuse studies

Complete-abuse studies (unlike incomplete-abuse studies) provided enough neglect data to allow a full five-form comparison. For risk, emotional abuse showed the strongest association (*k* = 81, OR = 3.79, 95% CI [3.39, 4.24]). Physical abuse (*k* = 81, OR = 2.98, 95% CI [2.66, 3.33]) was equivalent to physical neglect (*k* = 55, OR = 2.80, 95% CI [2.50, 3.14]). Emotional neglect (*k* = 58, OR = 2.49, 95% CI [2.23, 2.79]) was moderately lower. Sexual abuse (*k* = 81, OR = 2.06, 95% CI [1.84, 2.30]) was significantly lowest. Heterogeneity was very high, *Q*(351) = 5019.20, *p* < .001, *I* ^2^ = 93.0%.

For severity, emotional abuse (*k* = 87, *r* = .33; 95% CI, [.31, .35]) was significantly more correlated with depression than emotional neglect (*k* = 64, *r* = .30; 95% CI [.28, .32]), which was greater than physical neglect (*k* = 61, *r* = .22; 95% CI [.20, .25]), which was greater than physical abuse (*k* = 87, *r* = .20; 95% CI [.17, .22]). Sexual abuse was significantly least associated (*k* = 87, *r* = .17; 95% CI [.14, .19]). Heterogeneity remained high, *Q*(381) = 3087.26, *p* < .001, *I* ^2^ = 87.7%.

### Moderator analyses

Bivariate multilevel metaregressions were conducted for publication year, maltreatment measure, sample type, sample size, percentage of female participants, country, and language of assessment. Moderators satisfying *p* < .10 were entered into a multivariate model, when two or more moderators satisfied this criterion, with the following results.

For risk, the multivariate model was significant, *F*(5, 524) = 5.40, *p* < .001. Sample type, *F*(3, 524) = 5.14, *p* < .01, and sample size, *F*(1, 524) = 5.05, *p* = .025, were significant moderators, while percentage female, *F*(1, 524) = 2.78, *p* = .096, was a marginally significant moderator. Jointly, these moderators reduced unexplained heterogeneity by 23.5%. Case–control samples yielded larger effects than clinical samples. Smaller sample sizes yielded larger effect sizes, but only modestly (the spread from one standard deviation below to one above the mean was narrow; Table S2 in the Supplementary Material). Thus, there was minor evidence for small sample bias. Higher proportions of female participants were marginally significantly associated with larger effects.

For severity, multivariate metaregression was not performed because only one moderator met the criterion of *p* < .10. Sample type was significant, *F*(3, 496) = 4.06, *p* < .01, reducing residual heterogeneity modestly (*R* ^2^ change = 6.8%). Representative samples showed smaller effects than convenience samples (Table S3 in the Supplementary Material).

## Discussion

This study synthesized data from 12 meta-analytic reviews to provide the most methodologically comparable examination to date of how five forms of child maltreatment relate to depression. By restricting certain analyses to source studies that assessed most maltreatment forms – thereby reducing between-samples confounds – and by modeling statistical dependence via multilevel meta-analysis, we were able to evaluate cross-form differences with greater validity than prior reviews. Across analytic layers, emotional abuse consistently emerged as the form most strongly associated with depression, whereas sexual abuse yielded the weakest associations. Emotional neglect approached emotional abuse in magnitude in the severity analysis. Physical abuse and physical neglect showed intermediate associations. These findings refine the emerging conclusion that emotional maltreatment – particularly emotional abuse – has a strong link to depressive outcomes.

A key contribution of the present work is demonstrating how analytic design shapes cross-form comparisons. Complete-abuse studies, which assessed all three abuse forms within the same sample, provided the most internally valid test because they held sample characteristics constant across forms. In these studies, a consistent hierarchy emerged: emotional abuse > physical abuse > sexual abuse. This pattern appeared for both depression risk and severity and was replicated across effect size metrics. In contrast, analyses of incomplete-abuse studies – paralleling the structure of most prior reviews – showed much weaker differentiation. In these analyses, sexual abuse estimates increased to equal or nominally surpass those for physical abuse. Our results indicate that this inflation does not reflect true stronger effects but rather systematic differences in the populations and research designs used for certain maltreatment forms. This methodological artifact clarifies inconsistencies across the literature and underscores the necessity of complete-form samples for valid cross-form comparisons.

The multilevel analyses also confirmed that effect sizes for different maltreatment forms were statistically dependent within studies: correlations among forms were substantial (median *r*s ≈ .46–.48). This dependence is expected, given shared developmental pathways, environmental contexts, and measurement approaches across maltreatment types. If unmodeled, such dependence can distort pooled estimates and inflate Type I errors in pairwise contrasts. By modeling these dependencies directly through a multilevel structure, the present analyses yielded more accurate standard errors and effect size estimates than single-level models typically used in earlier reviews.

### Prominence of emotional maltreatment in depressive outcomes

Although emotional abuse consistently produced the largest associations with depression, emotional neglect was also relatively strongly associated – exceeding physical abuse and physical neglect in the severity analysis. This pattern aligns with theoretical perspectives emphasizing the importance of emotional communication, validation, and caregiver attunement for the development of depressive cognitions and emotion-regulation capacities (e.g. Rose & Abramson, 1992). Emotional maltreatment directly shapes interpersonal schemas and self-evaluative processes central to depression (Bowlby, 1982), and its theoretical coherence and biological plausibility (Bradford Hill, 1965) in relation to depression are supported by cross-cultural evidence (e.g. Khaleque & Rohner, 2002) and nonhuman primate models (e.g. Suomi, 1991, 1997, 2011).

In contrast, although sexual abuse has historically been regarded as uniquely harmful (Gardner et al., 2019; Infurna et al., 2016; Mandelli et al., 2015), its association with depression in the present analyses was notably smaller than that of emotional abuse. While emotional abuse produced medium-to-large effects (per Cohen, 1988; Salgado, 2018), sexual abuse yielded small-to-medium effects. This difference is clinically meaningful, particularly because emotional abuse is roughly three times more prevalent than sexual abuse, combining rates for boys and girls (World Health Organization, 2014). These findings support recent calls for greater prioritization of emotional abuse in global prevention initiatives (World Health Organization, 2014).

### Limitations

Several limitations should be noted. First, most source studies relied on retrospective self-reports, which may inflate associations of maltreatment with maladjustment relative to prospective research (Baldwin, Coleman, Francis, & Danese, 2024). Second, although multilevel modeling accounted for within-study dependencies, substantial residual heterogeneity remained, suggesting the influence of additional moderators – such as genetic factors, cultural norms, maltreatment chronicity, and co-occurring adversities (Baldwin et al., 2023a, 2023b; Khan et al., 2015). Third, because this study was a review of reviews, certain relevant original studies may have been missed, especially more recent ones.

Finally, caution is needed in assuming causal effects in the present findings, given potential confounds. Forms of child maltreatment frequently co-occur, and observed associations with depression may therefore be only partially causal. Or they may reflect preexisting vulnerabilities that both increase the likelihood of maltreatment exposure and contribute to later maladjustment (Baldwin et al., 2023a, 2023b, 2024; Humphreys et al., 2020; Infurna et al., 2016). Genetically informed meta-analytic evidence indicates that genetic liability accounts for a substantial proportion of the risk of exposure to child maltreatment (approximately 40%; Dahoun et al., 2025). Further, associations between maltreatment and mental health outcomes are markedly attenuated (by approximately 45%) when adjusting for nonmaltreatment factors, including genetic influences (Baldwin et al., 2023a). Accordingly, estimates of the relative strength of associations across maltreatment forms in this study should be interpreted as descriptive patterns rather than direct indicators of causal impact.

### Conclusion

Despite these limitations, the present findings provide a strengthened empirical foundation for theory, prevention, and intervention. The consistent prominence of emotional abuse – and, to a lesser degree, emotional neglect – suggests that emotional maltreatment warrants increased attention in clinical assessment and public-health prevention efforts, which have historically emphasized physical and sexual abuse. Future work should further refine models of emotional maltreatment, examine developmental timing and subtype distinctions, and prioritize designs that assess multiple maltreatment forms within the same sample to permit valid comparative inferences.

Overall, this study offers a methodologically rigorous synthesis showing that emotional abuse has the strongest association with depression, whereas sexual abuse has the weakest. By reducing between-samples confounds and explicitly modeling statistical dependence, the present analyses resolved several longstanding inconsistencies and reinforced the fundamental link between emotional maltreatment and depressive outcomes.

## Supporting information

10.1017/S003329172610381X.sm001 Rind and Rieger supplementary material Rind and Rieger supplementary material

## Supplementary material

The supplementary material for this article can be found at http://doi.org/10.1017/S003329172610381X.
