# Supplementary material for: Associations between five forms of child maltreatment and depression: a multilevel meta-analytic comparison
Source: Psychol Med. 2026 Apr 6;56:e89. doi: 10.1017/S003329172610381X (PMC13079234; doi:10.1017/S003329172610381X)
Supplement: Rind and Rieger supplementary material [file S003329172610381Xsup001.docx]

| **Table S1** |  |  |  |  |  |
| --- | --- | --- | --- | --- | --- |
| Correlations of effect sizes in child maltreatment pairs across samples based on complete-abuse samples | | | | | |
| Risk (ln OR) |  | EA | EN | PA | PN |
| Emotional neglect (EN) | *r* | .67** |  |  |  |
|  | *k* | 58 |  |  |  |
| Physical abuse (PA) | *r* | .53** | .42** |  |  |
|  | *k* | 81 | 58 |  |  |
| Physical neglect (PN) | *r* | .35* | .56** | .34* |  |
|  | *k* | 55 | 55 | 55 |  |
| Sexual abuse (SA) | *r* | .59** | .49** | .42** | .33* |
|  | *k* | 81 | 58 | 81 | 55 |
| Severity (Fisher *z*) |  | EA | EN | PA | PN |
| Emotional neglect (EN) | *r* | .42** |  |  |  |
|  | *k* | 64 |  |  |  |
| Physical abuse (PA) | *r* | .42** | .47** |  |  |
|  | *k* | 87 | 64 |  |  |
| Physical neglect (PN) | *r* | .31* | .62** | .65** |  |
|  | *k* | 61 | 60 | 61 |  |
| Sexual abuse (SA) | *r* | .50** | .23 | .61** | .49** |
|  | *k* | 87 | 64 | 87 | 61 |
| EA = emotional abuse; *r* = Pearson correlation; *k* = number of samples. Complete-abuse samples are those in which, at the minimum, EA, PA, and SA were all assessed. Risk correlations based on log odds (ln OR); severity correlations based on Fisher *z*s | | | | | |
| ** *p* < .01; * *p* < .05 |  |  |  |  |  |

| **Table S2** | |  |  |  |
| --- | --- | --- | --- | --- |
| Moderator analyses for risk effect sizes (OR) via multilevel meta-analyses of child maltreatment forms in relation to depression | | | | |
|  | *F* | *df*₁ | *df*₂ | *p* |
| Bivariate |  |  |  |  |
| Year of publish | 0.20 | 1 | 561 | 0.651 |
| CM measure | 1.23 | 5 | 553 | 0.293 |
| Sample type | 6.26 | 3 | 556 | **< .001** |
| Sample size (*N*) | 10.71 | 1 | 561 | **0.001** |
| Percent female | 3.37 | 1 | 528 | **0.067** |
| Country, region | 0.63 | 4 | 558 | 0.639 |
| English speaking | 0.81 | 1 | 556 | 0.368 |
| Multivariate*^a^* | 5.40 | 5 | 524 | **< .001** |
| Sample type | 5.14 | 3 | 524 | **0.002** |
| Sample size (*N*) | 5.05 | 1 | 524 | **0.025** |
| Percent female | 2.78 | 1 | 524 | 0.096 |
| *a* Significant (*p* < .05) or marginally significant (*p* < .10) bivariate moderators entered into multivariate meta-regression | | | | |
|  |  |  |  |  |
| Levels of moderators in multivariate meta-regression | | | | |
|  | OR | LL | UL | post hoc*^a^* |
| Sample type |  |  |  |  |
| Case-control | 3.07 | 2.70 | 3.49 | a |
| Clinical | 2.05 | 1.73 | 2.43 | b |
| Convenience | 2.47 | 2.21 | 2.75 | ab |
| Representative | 2.42 | 2.13 | 2.76 | ab |
| Sample size (*N*) |  |  |  |  |
| Mean - 1SD | 2.61 | 2.39 | 2.85 | a |
| Mean | 2.48 | 2.31 | 2.65 | ab |
| Mean + 1SD | 2.35 | 2.17 | 2.53 | b |
| Percentage female |  |  |  |  |
| Mean - 1SD | 2.36 | 2.15 | 2.58 | a |
| Mean | 2.48 | 2.31 | 2.65 | a |
| Mean + 1SD | 2.60 | 2.39 | 2.83 | a |
| *a* Levels without common letters (in last column) were significantly different (for sample types, Bonferroni-adjusted alpha level = .0083). For sample size, estimates of +1SD and -1SD were significantly different from each other but not from the mean; for percentage female, this contrast (+1SD vs. -1SD) was marginally significant | | | | |
|  |  |  |  |  |
| Simple / Nested Summary*^a^* | |  |  |  |
| Nested variable | *σ*² | Levels | *R*^2^ reduction |  |
| Study |  |  |  |  |
| Without moderators | 0.200 | 226 |  |  |
| With moderators | 0.153 | 208 | 23.50% |  |
| *a* *σ*² (estimated between-study variance) is the same as *τ*², the residual heterogeneity unexplained  *Note*. Included moderators = sample type, sample size, percentage female | | | |  |

| **Table S3** | |  |  |  |
| --- | --- | --- | --- | --- |
| Moderator analyses for severity effect sizes (*z*r) via multilevel meta-analyses of child maltreatment forms in relation to depression | | | | |
|  | F | df₁ | df₂ | p |
| Bivariate |  |  |  |  |
| Year of publish | 0.27 | 1 | 499 | 0.604 |
| CM measure*^a^* | 1.47 | 2 | 498 | 0.232 |
| Sample type | 4.06 | 3 | 496 | **0.007** |
| Sample size (*N*) | 0.20 | 1 | 499 | 0.659 |
| Percent female | 0.89 | 4 | 496 | 0.469 |
| Country, region | 0.23 | 1 | 495 | 0.631 |
| English speaking | 0.53 | 1 | 499 | 0.466 |
| *a* CM measure for severity studies: 1 = CTQ; 0 = other | | | |  |
| *Note*. Multivariate meta-regression not performed because only one variable was a significant (*p* < .05) or marginally significant (*p* < .10) bivariate moderator | | | | |
|  |  |  |  |  |
| Levels of significant moderator (sample type) | | | | |
|  | r | LL | UL | post hoc*^a^* |
| Sample type |  |  |  |  |
| Case-control | 0.20 | 0.10 | 0.29 | ab |
| Clinical | 0.23 | 0.19 | 0.28 | ab |
| Convenience | 0.28 | 0.26 | 0.30 | a |
| Representative | 0.14 | 0.05 | 0.23 | b |
| *a* Sample types without common letters (in last column) were significantly different at Bonferroni-adjusted alpha level of .0083 | | | | |
|  |  |  |  |  |
| Simple / Nested Summary^a^ | |  |  |  |
| Nested variable | σ² | Levels | R^2^ reduction |  |
| Study |  |  |  |  |
| Without moderators | 0.0135 | 162 |  |  |
| With moderators | 0.0125 | 161 | 6.80% |  |
| *a* *σ*² (estimated between-study variance) is the same as *τ*², the residual heterogeneity unexplained | | | |  |
| *Note*. Included moderator = sample type | | | |  |

| **References (risk studies)** |
| --- |
| Afifi, M. (2006). Depression in adolescents: Gender differences in Oman and Egypt. *Eastern Mediterranean Health Journal, 12*(1-2), 61–71. |
| Afifi, M., Al Riyami, A., Morsi, M., & Al Kharusil, H. (2006). Depressive symptoms among high school adolescents in Oman. *Eastern Mediterranean Health Journal, 12*(Suppl. 2), S126–S137. |
| [Afifi, T. O., Brownridge, D. A., Cox, B. J., & Sareen, J. (2006). Physical punishment, childhood abuse and psychiatric disorders. *Child Abuse & Neglect, 30*(10), 1093–1103. https://doi.org/10.1016/j.chiabu.2006.04.006](https://doi.org/10.1016/j.chiabu.2006.04.006) |
| [Afifi, T. O., Mota, N. P., Dasiewicz, P., MacMillan, H. L., & Sareen, J. (2012). Physical punishment and mental disorders: Results from a nationally representative US sample. *Pediatrics, 130*(2), 184–192. https://doi.org/10.1542/peds.2011-2947](https://doi.org/10.1542/peds.2011-2947) |
| Agbaje, O. S., Nnaji, C. P., Nwagu, E. N., Iweama, C. N., Umoke, P. C. I., Ozoemena, L. E., & Abba, C. C. (2021). Adverse childhood experiences and psychological distress among higher education students in Southeast Nigeria: An institutional-based cross-sectional study. *Archives of Public Health, 79*(1), 62. https://doi.org/10.1186/s13690-021-00587-3 |
| Ahmadkhaniha, H.R. (2007). The frequency of sexual abuse and depression in a sample of street children of one of deprived districts of Tehran. *Journal of Child Sexual Abuse.* 16, 37-61. |
| Allen, D. M., & Tarnowski, K. J. (1989). Depressive characteristics of physically abused children. *Journal of Abnormal Child Psychology, 17*(1), 1-11. |
| Allers, C.T., & Benjack, K.J. (1991). Connections between childhood abuse and HIV infection. *Journal of Counseling & Development, 70*(2), 309-13. |
| Almeida, O.P., Alfonso, H., Pirkis, J., Kerse, N., Sim, M., Flicker, L., et al. (2011) A practical approach to assess depression risk and to guide risk reduction strategies in later life. *International Psychogeriatrics*, *23*(2), 28091. |
| Anda, R.F., Whitﬁeld, C.L., Felitti, V.J., Chapman, D., Edwards, V.J., Dube, S.R., et al. (2002). Adverse childhood experiences, alcoholic parents, and later risk of alcoholism and depression. *Psychiatric Services, 53*, 1001–9. |
| Angst, J., Gamma, A., Rössler, W., Ajdacic, V., & Klein, D.N. (2011). Childhood adversity and chronicity of mood disorders. *European Archives of Psychiatry and Clinical Neuroscience,* *261*(1), 21-7. |
| Appel, K., Schwahn, C., Mahler, J., Schulz, A., Spitzer, C., Fenske, K. et al. Moderation of adult depression by a polymorphism in the FKBP5 gene and childhood physical abuse in the general population. *Neuropsychopharmacology* 2011; *36*(10):1982-91. |
| Armour, C., Mullerova, J., Fletcher, S., Lagdon, S., Burns, C. R., Robinson, M., ... Robinson, J. (2016). Assessing childhood maltreatment and mental health correlates of disordered eating profiles in a nationally representative sample of English females. *Social Psychiatry and Psychiatric Epidemiology, 51*(3), 383–393. |
| [As-Sanie, S., Clevenger, L. A., Geisser, M. E., Williams, D. A., & Roth, R. S. (2014). History of abuse and its relationship to pain experience and depression in women with chronic pelvic pain. *American Journal of Obstetrics & Gynecology, 210*(4), 317 e311-317 e318. https://doi.org/10.1016/j.ajog.2013.12.048](https://doi.org/10.1016/j.ajog.2013.12.048) |
| Avanci, J. Q., Assis, S. G., & Oliveira, R. V. (2008). Depressive symptoms during adolescence: a study on psychosocial factors in a sample of teenage students in a city in Rio de Janeiro State, Brazil. *Cadernos de Saude Publica, 24*(10), 2334–2346. |
| Avanci, J., Assis, S., Oliveira, R., & Pires, T. (2012). Childhood depression. Exploring the association between family violence and other psychosocial factors in low-income Brazilian schoolchildren. *Child Adolesc Psychiatry Ment Health, 6*, 1–9. doi: 10.1186/1753-2000-6-26 |
| Bandelow, B., Gutermann, J., Peter, H., & Wedekind, D. (2013). Early traumatic life events, parental attitudes, family history, and birth risk factors in patients with depressive disorder and healthy controls. *International Journal of Psychiatry in Clinical Practice,* *17*(1), 56-63. |
| [Bauriedl-Schmidt, C., Jobst, A., Gander, M., Seidl, E., Sabaß, L., Sarubin, N., ... Buchheim, A. (2017). Attachment representations, patterns of emotion regulation, and social exclusion in patients with chronic and episodic depression and healthy controls. *Journal of Affective Disorders, 210*, 130–138. https://doi.org/10.1016/j.jad.2016.12.030](https://doi.org/10.1016/j.jad.2016.12.030) |
| Benedict, M.I., Paine, L.L., Paine, L.A., Brandt, D., & Stallings, R. (1999). The association of childhood sexual abuse with depressive symptoms during pregnancy and selected pregnancy outcomes. *Child Abuse & Neglect,* *23*(7), 659-70. |
| [Bermingham, R., Carballedo, A., Lisiecka, D., Fagan, A., Morris, D., Fahey, C., ... Frodl, T. (2012). Effect of genetic variant in BICC1 on functional and structural brain changes in depression. *Neuropsychopharmacology, 37*(13), 2855–2862. https://doi.org/10.1038/npp.2012.158](https://doi.org/10.1038/npp.2012.158) |
| Bernet, C. Z., & Stein, M. B. (1999). Relationship of childhood maltreatment to the onset and course of major depression in adulthood. *Depression and Anxiety, 9*(4), 169–174. |
| Bifulco, A., Brown, G.W., & Adler, Z.Z. (1991). Early sexual abuse and clinical depression in adult life. *British Journal of Psychiatry,* *159*, 115-22. |
| Bifulco, A., Moran, P.M., Ball, C., Jacobs, C., Baines, R., Bunn, A., & Cavagin, J. (2002b). Childhood adversity, parental vulnerability and disorder: examining intergenerational transmission of risk. *Journal of Child Psychology and Psychiatry, 43*, 1075–1086. |
| Blanchard, E. B., Keefer, L., Payne, A., Turner, S. M., & Galovski, T. E. (2002). Early abuse, psychiatric diagnoses and irritable bowel syndrome. *Behaviour Research and Therapy, 40*, 289-298. |
| [Boecking, B., & Barnhofer, T. (2014). ‘She called her partner–hence she is needy’: Depressed patients show increased tendencies to make spontaneous trait inferences. *Psychological Medicine, 44*(14), 2995–3006. https://doi.org/10.1017/S0033291714000622](https://doi.org/10.1017/S0033291714000622) |
| [Bonomi, A. E., Cannon, E. A., Anderson, M. L., Rivara, F. P., & Thompson, R. S. (2008). Association between self-reported health and physical and/or sexual abuse experienced before age 18. *Child Abuse & Neglect, 32*(7), 693–701. https://doi.org/10.1016/j.chiabu.2007.10.004](https://doi.org/10.1016/j.chiabu.2007.10.004) |
| Brezo, J., Bureau, A., Merette, C., Jomphe, V., Barker, E.D., Vitaro, F. et al. Differences and similarities in the serotonergic diathesis for suicide attempts and mood disorders: a 22-year longitudinal gene-environment study. *Molecular Psychiatry, 15*, 831–43. |
| Briere, J., & Runtz, M. (1988). Symptomatology associated with childhood sexual victimization in a nonclinical adult sample. *Child Abuse & Neglect, 12*(1), 51-59. |
| Briere, J., Woo, R., McRae, B., Foltz, J., & Sitzman, R. (1997). Lifetime victimization history, demographics, and clinical status in female psychiatric emergency room patients. *The Journal of Nervous and Mental Disease,* *185*(2), 95-101. |
| Brock, K. J., Mintz, L. B., & Good, G. E. (1997). Differences among sexually abused and nonabused women from functional and dysfunctional families. *Journal of Counseling Psychology, 44*(4), 425. |
| [Brown, J., Cohen, P., Johnson, J. G., & Smailes, E. M. (1999). Childhood abuse and neglect: Specificity and effects on adolescent and young adult depression and suicidality. *Journal of the American Academy of Child and Adolescent Psychiatry, 38*(12), 1490–1496. https://doi.org/10.1097/00004583-199912000-00009](https://doi.org/10.1097/00004583-199912000-00009) |
| Bulik, C.M., Sullivan, P.F., & Rorty, M. (1989). Childhood sexual abuse in women with bulimia*. The Journal of Clinical Psychiatry,* *50*(12), 460-4. |
| [Carballedo, A., Morris, D., Zill, P., Fahey, C., Reinhold, E., Meisenzahl, E., ... Frodl, T. (2013). Brain-derived neurotrophic factor Val66Met polymorphism and early life adversity affect hippocampal volume. *American Journal of Medical Genetics Part B: Neuropsychiatric Genetics, 162*(2), 183–190. https://doi.org/10.1002/ajmg.b.32130](https://doi.org/10.1002/ajmg.b.32130) |
| Carey, P.D., Walker, J.L., Rossouw, W., Seedat, S., & Stein, D.J. (2008). Risk indicators and psychopathology in traumatised children and adolescents with a history of sexual abuse. *European Child and Adolescent Psychiatry*, *17*, 93-98. |
| [Chaney, A., Carballedo, A., Amico, F., Fagan, A., Skokauskas, N., Meaney, J., & Frodl, T. (2014). Effect of childhood maltreatment on brain structure in adult patients with major depressive disorder and healthy participants. *Journal of Psychiatry & Neuroscience, 39*(1), 50–59. https://doi.org/10.1503/jpn.120208.](https://doi.org/10.1503/jpn.120208) |
| [Chapman, D. P., Whitfield, C. L., Felitti, V. J., Dube, S. R., Edwards, V. J., & Anda, R. F. (2004). Adverse childhood experiences and the risk of depressive disorders adulthood. *Journal of Affective Disorders, 82*(2), 217–225. https://doi.org/10.1016/j.jad.2003.12.013](https://doi.org/10.1016/j.jad.2003.12.013) |
| Cheasty, M., Clare, A.W., & Collins, C. (1998). Relation between sexual abuse in childhood and adult depression: case-control study. *BMJ*, *316*, 198–201. |
| [Chou, K.-L. (2012). Childhood sexual abuse and psychiatric disorders in middle-aged and older adults: Evidence from the 2007 Adult Psychiatric Morbidity Survey. *Journal of Clinical Psychiatry, 73*(11), e1365–e1371. https://doi.org/10.4088/JCP.12m07946](https://doi.org/10.4088/JCP.12m07946) |
| Chung, E.K., Mathew, L., Elo, I.T., Coyne, J.C., Culhane, J.F. (2008). Depressive symptoms in disadvantaged women receiving prenatal care: the inﬂuence of adverse and positive childhood experiences. *Ambulatory Pediatrics, 8*, 109–16. |
| [Cisler, J. M., James, G. A., Tripathi, S., Mletzko, T., Heim, C., Hu, X. P., Mayber, H. S., Nemeroff, C. B., & Kilts, C. D. (2013). Differential functional connectivity within an emotion regulation neural network among individuals resilient and susceptible to the depressogenic effects of early life stress. *Psychological Medicine, 43*(3), 507–518. https://doi.org/10.1017/S0033291712001390](https://doi.org/10.1017/S0033291712001390) |
| Cohen, Y., Spirito, A., Sterling, C., et al. (1996). Physical and sexual abuse and their relation to psychiatric disorder and suicidal behavior among adolescents who are psychiatrically hospitalized. *Journal of Child Psychology and Psychiatry, 37*, 989-993. |
| Cohen, P., Brown, J., & Smaile, E. (2001). Child abuse and neglect and the development of mental disorders in the general population. *Developmental Psychopathology, 13*(4), 981–999. |
| [Coles, J., Lee, A., Taft, A., Mazza, D., & Loxton, D. (2015). Childhood sexual abuse and its association with adult physical and mental health: Results from a national cohort of young Australian women. *Journal of Interpersonal Violence, 30*(11), 1929–1944. https://doi.org/10.1177/0886260514555270](https://doi.org/10.1177/0886260514555270) |
| [Comijs, H. C., Van Exel, E., Van der Mast, R. C., Paauw, A., Voshaar, R. O., & Stek, M. L. (2013). Childhood abuse in late-life depression. *Journal of Affective Disorders, 147*(1–3), 241–246. https://doi.org/10.1016/j.jad.2012.11.010](https://doi.org/10.1016/j.jad.2012.11.010) |
| Cong, E., Li, Y., Shao, C., Chen, J., Wu, W., Shang, X., et al. (2012). Childhood sexual abuse and the risk for recurrent major depression in Chinese women. *Psychological Medicine*, *42*(2), 409-17. |
| Cyranowski, J.M., Schott, L.L., Kravitz, H.M., Brown, C., Thurston, R.C., Joffe, H., et al. (2012). Psychosocial features associated with lifetime comorbidity of major depression and anxiety disorders among a community sample of mid-life women: The Swan Mental Health Study. *Depression and Anxiety*, *29*(12), 1050-7. |
| Danielson, C. K., de Arellano, M. A., Kilpatrick, D. G., Saunders, B. E., & Resnick, H. S. (2005). Child maltreatment in depressed adolescents: Differences in symptomatology based on history of abuse. *Child Maltreat.,10*, 37–48. doi: 10.1177/1077559504271630 |
| [Dannehl, K., Rief, W., & Euteneuer, F. (2017). Childhood adversity and cognitive functioning in patients with major depression. *Child Abuse & Neglect, 70*, 247–254. https://doi.org/10.1016/j.chiabu.2017.06.013](https://doi.org/10.1016/j.chiabu.2017.06.013) |
| Daviss, W.B., Diler, R.S., Birmaher, B. (2009). Associations of lifetime depression with trauma exposure, other environmental adversities, and impairment in adolescents with ADHD. *Journal of Abnormal Child Psychology, 37*, 857-871. |
| Dennis, M.F., Flood, A.M., Reynolds, V., Araujo, G., Clancy, C.P., Barefoot, J.C., et al. (2009). Evaluation of lifetime trauma exposure and physical health in women with posttraumatic stress disorder or major depressive disorder. *Violence Against Women*, *15*(5), 618-27. |
| Deyessa, N., Berhane, Y., Alem, A., Ellsberg, M., Emmelin, M., Hogberg, U., et al. (2009). Intimate partner violence and depression among women in rural Ethiopia: A cross-sectional study. *Clinical Practice in Epidemiology and Mental Health, 5*. |
| Dhamayanti, M., Noviandhari, A., Masdiani, N., Pandia, V., & Sekarwana, N. (2020). The association of depression with child abuse among Indonesian adolescents. *BMC Pediatr., 20*, 1–6. doi: 10.1186/s12887-020-02218-2 |
| Dinwiddie, S., Heath, A. C., Dunne, M. P., Bucholz, K. K., Madden, P. A., Slutske, W. S., ... Martin, N. G. (2000). Early sexual abuse and lifetime psychopathology: A twin-control study. *Psychological Medicine, 30*(1), 41–52. |
| [Douglas, K. M., & Porter, R. (2012). The effect of childhood trauma on pharmacological treatment response in depressed inpatients. *Psychiatry Research, 200*(2-3), 1058–1061. https://doi.org/10.1016/j.psychres.2012.06.015.](https://doi.org/10.1016/j.psychres.2012.06.015) |
| [Du, L., Wang, J., Meng, B., Yong, N., Yang, X., Huang, Q., ... Li, Y. (2016). Early life stress affects limited regional brain activity in depression. *Scientific Reports, 6*, 1–8. https://doi.org/10.1038/srep25338](https://doi.org/10.1038/srep25338) |
| [Dunn, E. C., Gilman, S. E., Willett, J. B., Slopen, N. B., & Molnar, B. E. (2012). The impact of exposure to interpersonal violence on gender differences in adolescent-onset major depression: Results from the National Comorbidity Survey Replication (NCS-R). *Depression and Anxiety, 29*(5), 392–399. https://doi.org/10.1002/da.21916](https://doi.org/10.1002/da.21916) |
| [Dunn, E. C., McLaughlin, K. A., Slopen, N., Rosand, J., & Smoller, J. W. (2013). Developmental timing of child maltreatment and symptoms of depression and suicidal ideation in young adulthood: Results from the National Longitudinal Study of Adolescent Health. *Depression and Anxiety, 30*(10), 955–964. https://doi.org/10.1002/da.22102.](https://doi.org/10.1002/da.22102) |
| Duran, B., Malcoe, L.H., Sanders, M., Waitzkin, H., Skipper, B., & Yager, J. (2004). Child maltreatment prevalence and mental disorders outcomes among American Indian women in primary care. *Child Abuse & Neglect,* *28*(2), 131-45. |
| Ege, M.A., Messias, E., Thapa, P., &Krain, L.P. (2015). Adverse childhood experiences and geriatric depression: Results from the 2010 BRFSS. *The American Journal of Geriatric Psychiatry, 23(1)*, 110-114. |
| [Elm, J. H. L. (2020). Adverse childhood experiences and internalizing symptoms among American Indian adults with type 2 diabetes. *Journal of Racial and Ethnic Health Disparities, 7*(5), 958–966. https://doi.org/10.1007/s40615-020-00720-y](https://doi.org/10.1007/s40615-020-00720-y) |
| Ernst, C., Angst, J., & Földényi, M. (1993). The Zurich study, XVII: Sexual abuse in childhood: Frequency and relevance for adult morbidity data of a longitudinal epidemiological study. *European Archives of Psychiatry and Clinical Neuroscience,* *242*(5), 293-300. |
| [Fergusson, D. M., Boden, J. M., & Horwood, L. J. (2008). Exposure to childhood sexual and physical abuse and adjustment in early adulthood. *Child Abuse & Neglect, 32*(6), 607–619. https://doi.org/10.1016/j.chiabu.2006.12.018](https://doi.org/10.1016/j.chiabu.2006.12.018) |
| [Fergusson, D. M., Horwood, L. J., & Lynskey, M. T. (1996). Childhood sexual abuse and psychiatric disorder in young adulthood: II. Psychiatric outcomes of childhood sexual abuse. *Journal of the American Academy of Child and Adolescent Psychiatry, 35*(10), 1365–1374. https://doi.org/10.1097/00004583-199610000-00024](https://doi.org/10.1097/00004583-199610000-00024) |
| Fergusson, D.M., McLeod, G.F.H., & Horwood, L.J. (2013). Childhood sexual abuse and adult developmental outcomes: Findings from a 30-year longitudinal study in New Zealand. *Child Abuse & Neglect*, *37*(9), 664-7459. |
| [Fernando, S. C., Beblo, T., Schlosser, N., Terfehr, K., Otte, C., Löwe, B., ... Wingenfeld, K. (2014). The impact of self-reported childhood trauma on emotion regulation in borderline personality disorder and major depression. *Journal of Trauma & Dissociation, 15*(4), 384–401. https://doi.org/10.1080/15299732.2013.863262](https://doi.org/10.1080/15299732.2013.863262) |
| Figueroa, E.F., Silk, K.R., Huth, A., & Lohr, N.E. (1997). History of childhood sexual abuse and general psychopathology. *Comprehensive Psychiatry,* *38*(1), 23-30. |
| Fisher, H.L., Cohen-Woods, S., Hosang, G.M., Korszum, A., Owen, M., Craddock, N., et al. (2013). Interaction between specific forms of childhood maltreatment and the serotonin transporter gene (5-HTT) in recurrent depressive disorder. *Journal of Affective Disorders,* *145*(1), 136-41. |
| Fisher, H.L., Craig, T.K., Fearon, P., Morgan, K., Dazzan, P., Lappin, J., et al. (2011). Reliability and comparability of psychosis patients’ retrospective reports of childhood abuse. *Schizophrenia Bulletin*, *37*(3), 546-53. |
| Flisher, A.J., Psych, F.C., Kramer, R.A., et al. (1997). Psychosocial characteristics of physically abused children and adolescents. *Journal of the American Academy of Child and Adolescent Psychiatry, 36*, 123-131. |
| Friedman, S., Smith, L., Fogel, D., Paradis, C., Viswanathan, R., Ackerman, R., et al. (2002). The incidence and influence of early traumatic life events in patients with panic disorder: A comparison with other psychiatric outpatients. *Journal of Anxiety Disorders, 16*(3), 259-72. |
| [Frodl, T., Janowitz, D., Schmaal, L., Tozzi, L., Dobrowolny, H., Stein, D. J., ... Block, A. (2017). Childhood adversity impacts on brain subcortical structures relevant to depression. *Journal of Psychiatric Research, 86*, 58–65. https://doi.org/10.1016/j.jpsychires.2016.11.010](https://doi.org/10.1016/j.jpsychires.2016.11.010) |
| Gallo, E. A. G., De Mola, C. L., Wehrmeister, F., Goncalves, H., Kieling, C., & Murray, J. ̧(2017). Childhood maltreatment preceding depressive disorder at age 18 years: a prospective Brazilian birth cohort study. *J Affect Disord., 217*, 218–224. doi: 10.1016/j.jad.2017.03.065 |
| Garabedian, M.J., Lain, K.Y., Hansen, W.F., Garcia, L.S., Williams, C.M., & Crofford, L.J. (2011). Violence against women and postpartum depression. *Journal of Women’s Health,* *20*(3), 447-53. |
| Gibb, B. E., Chelminski, I., & Zimmerman, M. (2007). Childhood emotional, physical, and sexual abuse, and diagnoses of depressive and anxiety disorders in adult psychiatric outpatients. *Depression & Anxiety, 24*(4), 256-263. |
| Gonzalez, A., Boyle, M.H., Kyu, H.H., Georgiades, K., Duncan, L., & MacMillan, H.L. (2012). Childhood and family influences on depression, chronic physical conditions, and their comorbidity: findings from the Ontario Child Health Study. *Journal of Psychiatric Research,* *46*(11), 1475-82. |
| [Grant, M. M., White, D., Hadley, J., Hutcheson, N., Shelton, R., Sreenivasan, K., & Deshpande, G. (2014). Early life trauma and directional brain connectivity within major depression. *Human Brain Mapping, 35*(9), 4815–4826. https://doi.org/10.1002/hbm.22514](https://doi.org/10.1002/hbm.22514) |
| [Grassi-Oliveira, R., de Azevedo Gomes, C. F., & Stein, L. M. (2011). False recognition in women with a history of childhood emotional neglect and diagnose of recurrent major depression. *Consciousness and Cognition, 20*(4), 1127–1134. https://doi.org/10.1016/j.concog.2011.03.005](https://doi.org/10.1016/j.concog.2011.03.005) |
| Greger, K. H., Myhre, A. K., Lydersen, S., & Jozeﬁak, T. (2015). Previous maltreatment and present mental health in a high-risk adolescent population. *Child Abuse & Neglect, 45*, 122-134. |
| [Grosse, L., Ambrée, O., Jörgens, S., Jawahar, M. C., Singhal, G., Stacey, D., Arolt, V., & Baune, B. T. (2016). Cytokine levels in major depression are related to childhood trauma but not to recent stressors. *Psychoneuroendocrinology, 73*, 24–31. https://doi.org/10.1016/j.psyneuen.2016.07.205](https://doi.org/10.1016/j.psyneuen.2016.07.205) |
| [Güleç, M. Y., Altintas, M., Inanç, L., Bezgin, Ç. H., Koca, E. K., & Güleç, H. (2013). Effects of childhood trauma on somatization in major depressive disorder: The role of alexithymia. *Journal of Affective Disorders, 146*(1), 137–141. https://doi.org/10.1016/j.jad.2012.06.033](https://doi.org/10.1016/j.jad.2012.06.033) |
| Haj-Yahia, M. M., & Tamish, S. (2001). The rates of child sexual abuse and its psychological consequences as revealed by a study among Palestinian university students. *Child Abuse & Neglect, 25*(10), 1303-1327. |
| Hanson, R.F., Borntrager, C., Self-Brown, S., et al. (2008). Relations among gender, violence exposure, and mental health: the National Survey of Adolescents. *American Journal of Orthopsychiatry, 78*, 313-321. |
| Harrop-Griffiths, J., Katon, W., Walker, E., Holm, L., Russo, J., & Hickok, L. (1988). The association between chronic pelvic pain, psychiatric diagnoses, and childhood sexual abuse. *Obstetrics and Gynecology, 71*(4), 589-594. |
| [Hauer, B. J., Wessel, I., Geraerts, E., Merckelbach, H., & Dalgleish, T. (2008). Autobiographical memory specificity after manipulating retrieval cues in adults reporting childhood sexual abuse. *Journal of Abnormal Psychology, 117*(2), 444-453. https://doi.org/10.1037/0021-843X.117.2.444](https://doi.org/10.1037/0021-843X.117.2.444) |
| He, J., Zhong, X., Gao, Y., Xiong, G., & Yao, S. (2019). Psychometric properties of the Chinese version of the childhood trauma questionnaire-short form (CTQ-SF) among undergraduates and depressive patients. *Child Abuse & Neglect, 91*, 102–108. https://doi.org./10.1016/j.chiabu.2019.03.009. |
| Henny, K.D., Kidder, D.P., Stall, R., & Wolitski, R.J. (2007). Physical and sexual abuse among homeless and unstably housed adults living with HIV: Prevalence and associated risks. *AIDS and Behavior*, *11*(6), 84253. |
| Hill, J., Davis, R., Byatt, M., Burnside, E., Rollinson, L., & Fear, S. (2000). Childhood sexual abuse and affective symptoms in women: A general population study. *Psychological Medicine,* *30*(6), 1283-91. |
| Hill, J., Pickles, A., Burnside, E., Byatt, M., Rollinson, L., Davis, R., & Harvey, K., (2001). Child sexual abuse, poor parental care and adult depression: evidence for different mechanisms. *The British Journal of Psychiatry, 179*, 104–109. |
| Horesh, N., Sever, J., & Apter, A. (2003). A comparison of life events between suicidal adolescents with major depression and borderline personality disorder. *Comprehensive Psychiatry, 44*, 277-283. |
| [Hovens, J. G., Giltay, E. J., Spinhoven, P., van Hemert, A. M., & Penninx, B. W. (2015). Impact of childhood life events and childhood trauma on the onset and recurrence of depressive and anxiety disorders. *The Journal of Clinical Psychiatry, 76*(7), 931–938. https://doi.org/10.4088/JCP.14m09135](https://doi.org/10.4088/JCP.14m09135) |
| Hovens, J.G., Giltay, E.J., Wiersma, J.E., Spinhoven, P., Penninx, B.W., & Zitman, F.G. (2012). Impact of childhood life events and trauma on the course of depressive and anxiety disorders. *Acta Psychiatrica Scandinavica,* *126*(3), 198-207. |
| Hughes, H. M. (1988). Psychological and behavioral correlates of family violence in child witnesses and victims. *American Journal of Orthopsychiatry, 58*(1), 77-90. |
| [Huu, T. N., Anh, L. V., Peltzer, K., Pengpid, S., Low, W. Y., & Win, H. H. (2017). Childhood emotional, physical, and sexual abuse and associations with mental health and health-risk behaviors among university students in the Association of Southeast Asian Nations (ASEAN). *Child Studies in Asia-Pacific Contexts, 7*(1), 15–26. https://doi.org/10.5723/csac.2017.7.1.015](https://doi.org/10.5723/csac.2017.7.1.015) |
| Jaffee, S.R., Mofﬁtt, T.E., Caspi, A., Fombonne, E., Poulton, R., & Martin, J. (2002). Differences in early childhood risk factors for juvenile-onset and adult-onset depression. *Archives of General Psychiatry, 59*, 215-222. |
| [Jaschek, G., Carter-Pokras, O., He, X., Lee, S., & Canino, G. (2016). Association of child maltreatment and depressive symptoms among Puerto Rican youth. *Child Abuse & Neglect, 58*, 63-71. https://doi.org/10.1016/j.chiabu.2016.06.016](https://doi.org/10.1016/j.chiabu.2016.06.016) |
| [Jewkes, R. K., Dunkle, K., Nduna, M., Jama, P. N., & Puren, A. (2010). Associations between childhood adversity and depression, substance abuse incident infections in rural South African youth. *Child Abuse & Neglect, 34*(11), 833–841. https://doi.org/10.1016/j.chiabu.2010.05.002](https://doi.org/10.1016/j.chiabu.2010.05.002) |
| [Jobst, A., Sabass, L., Palagyi, A., Bauriedl-Schmidt, C., Mauer, M. C., Sarubin, N., ... Padberg, F. (2015). Effects of social exclusion on emotions and oxytocin and cortisol levels in patients with chronic depression. *Journal of Psychiatric Research, 60*, 170–177. https://doi.org/10.1016/j.jpsychires.2014.11.001](https://doi.org/10.1016/j.jpsychires.2014.11.001) |
| Johnson, A.L., Gibb, B.E., & McGeary, J. (2010). Reports of childhood physical abuse, 5-HTTLPR genotype, and women’s attentional biases for angry faces. *Cognitive Therapy and Research,* *34*(4), 380-7. |
| [Jonas, S., Bebbington, P., McManus, S., Meltzer, H., Jenkins, R., Kuipers, E., ... Brugha, T. (2011). Sexual abuse and psychiatric disorder in England: 2007 Adult Psychiatric Morbidity Survey. *Psychological Medicine, 41*(4), 709–719. https://doi.org/10.1017/S003329171000111X](https://doi.org/10.1017/S003329171000111X) |
| Kaplan, S.J., Pelcovitz, D., Salzinger, S., et al. (1998). Adolescent physical abuse: risk for adolescent psychiatric disorders. *American Journal of Psychiatry, 155*, 954-959. |
| Kaufman, J. (1991). Depressive disorders in maltreated children. *Journal of the American Academy of Child and Adolescent Psychiatry, 30*, 257-265. |
| Kazdin, A. E., Moser, J., Colbus, D., & Bell, R. (1985). Depressive symptoms among physically abused and psychiatrically disturbed children. *Journal of Abnormal Psychology, 94*(3), 298-307. |
| Kendler, K. S., Bulik, C. M., Silberg, J., Hettema, J. M., Myers, J., & Prescott, C. A. (2000). Childhood sexual abuse and adult psychiatric and substance women: An epidemiological and cotwin control analysis. *Archives of General Psychiatry, 57*(10), 953–959. |
| Kilic, F., Coskun, M., Bozkurt, H., Kaya, I., & Zoroglu, S. (2017). Self-injury and suicide attempt in relation with trauma and dissociation among adolescents with dissociative and nondissociative disorders. *Psychiatry Investigation, 14*, 172-178. |
| Kilpatrick, D. G., Ruggiero, K. J., Acierno, R., Saunders, B. E., Resnick, H. S., & Best, C. L. (2003). Violence and risk of PTSD, major depression, dependence, and comorbidity: Results from the National Survey of Adolescents. *Journal of Consulting and Clinical Psychology, 71*(4), 692–700. |
| [Kim, H. S., & Kim, H. S. (2005). Incestuous experience among Korean adolescents: Prevalence, family problems, perceived family dynamics, and characteristics. *Public Health Nursing, 22*(6), 472–482. https://doi.org/10.1111/j.0737-1209.2005.220604.x](https://doi.org/10.1111/j.0737-1209.2005.220604.x) |
| [Kim, Y. H. (2017). Associations of adverse childhood experiences with depression and alcohol abuse among Korean college students. *Child Abuse & Neglect, 67*, 338–348. https://doi.org/10.1016/j.chiabu.2017.03.009](https://doi.org/10.1016/j.chiabu.2017.03.009) |
| Klein, D. N., Glenn, C. R., Kosty, D. B., Seeley, J. R., Rohde, P., & Lewinsohn, P. M. (2013). Predictors of first lifetime onset of major depressive adulthood. *Journal of Abnormal Psychology, 122*(1), 1–6. https://doi.org/10.1037/a0029567. |
| Klein, D. N., & Kotov, R. (2016). Course of depression in a 10-year prospective study: Evidence for qualitatively distinct subgroups, *Journal of* *Abnormal Psychology, 125*(3), 337. |
| Kolko, D.J., Moser, J.T., & Weldy, S.R. (1988). Behavioral/emotional indicators of sexual abuse in child psychiatric inpatients: a controlled comparison with physical abuse. *Child Abuse & Neglect, 12*, 529-541. |
| Kosseva, M., Schild, S., Wilhelm-Schwenk, R., Biewer, W., & Hauser, W. (2010). Komorbide depressive Störungen als Mediator der Assoziation von Misshandlungen in Kindheit/Jugend und Fibromyalgiesyndrom [Comorbid depression mediates the association of childhood/adolescent maltreatment and fibromyalgia syndrome. A study with patients from different clinical settings]. *Schmerz, 24*(5), 474-84. |
| Kounou, K.B., Bui, E., Dassa, K.S., Hinton, D., Fischer, L., Djassoa, G., et al. Childhood trauma, personality disorders symptoms and current major depressive disorder in Togo. *Social Psychiatry and Psychiatric Epidemiology,* *48*(7), 1095-103. |
| [Kuhlman, K. R., Maercker, A., Bachem, R., Simmen, K., & Burri, A. (2013). Developmental and contextual factors in the role of severe childhood trauma in geriatric depression: The sample case of former indentured child laborers. *Child Abuse & Neglect, 37*(11), 969–978. https://doi.org/10.1016/j.chiabu.2013.04.013](https://doi.org/10.1016/j.chiabu.2013.04.013) |
| Kuyken, W., Howell, R., & Dalgleish, T. (2016). Overgeneral autobiographical memory in depressed adolescents with, versus without, a reported history of trauma. *J Abnormal Psychol., 115*, 387. doi: 10.1037/0021-843X.115.3.387 |
| Lang, C. Y., & Huang, Q. M. (2006). Er tong qi nue dai yu cheng nian hou yi yu zheng de yan jiu [A study of childhood abuse and post-adult depression]. *Journal of Qilu Nursing*, *12*(13), 1227–1228. https://doi.org/10.3969/j.issn.1006–7256.2006.13.022. |
| Lenze, S.N., Xiong, C., & Sheline, Y.I. (2008). Childhood adversity predicts earlier onset of major depression but not reduced hippocampal volume. *Psychiatry Research, 162*, 39–49. |
| Lewis, R.J., Griffin, J.L., Winstead, B.A., Morrow, J.A., & Schubert, C.P. (2003). Psychological characteristics of women who do or do not report a history of sexual abuse. *Journal of Prevention & Intervention in the Community,* *26*(1), 4965. |
| [Li, Y., & Lu, J. (2020). Study of the effect of childhood adversity on depression among Chinese older adults. *Population Journal, 42*, 56-69. https://doi.org/10.36316/gcatr.01.0007](https://doi.org/10.36316/gcatr.01.0007) |
| Libby, A.M., Orton, H.D., Novins, D.K., Beals, J., & Manson, S.M. (2005). Childhood physical and sexual abuse and subsequent depressive and anxiety disorders for two American Indian tribes. *Psychological Medicine,* *35*(3), 329-40. |
| Ling, Y., Yang, J., Zhong, B., Zhang, C., & Yao, S. (2009). Effects of childhood traumatic experience and self-esteem on adolescents’ depression. *Chin J Clin Psychol., 17*, 54–56. doi: 10.16128/j.cnki.1005-3611.2009.01.036 |
| Liu, X., & Zhang, Y. B. (2021). Relationships between depressive symptoms and adverse childhood expericences of rural middle school students. *Chin J Child Health Care., 29*, 951–954 + 959. doi: 10.11852/zgetbjzz2021-001 |
| Lizardi, H., Klein, D. N., Ouimette, P. C., Riso, L. P., Anderson, R. L., & Donaldson, S. K. (1995). Reports of the childhood home environment in early-onset dysthymia and episodic major depression. *Journal of Abnormal Psychology, 104*(1), 132. |
| [Lu, S., Gao, W., Huang, M., Li, L., & Xu, Y. (2016). In search of the HPA axis activity in unipolar depression patients with childhood trauma: Combined cortisol awakening response and dexamethasone suppression test. *Journal of Psychiatric Research, 78*, 24–30. https://doi.org/10.1016/j.jpsychires.2016.03.009](https://doi.org/10.1016/j.jpsychires.2016.03.009) |
| Lu, X., Guo, H., Sun, J., Dong, Q., Zhao, F., Liao, X., Zhang, L., Zhang, Y., Li, W., Li, Z., Liu, T., He, Y., Xia, M., & Li, L. (2018). A shared effect of paroxetine treatment on gray matter volume in depressive patients with and without childhood maltreatment: A voxel‐based morphometry study. *CNS Neuroscience & Therapeutics, 24*(11), 1073–1083. https://doi.org/10.1111/cns.13055. |
| MacGiollabhui, N., Hamilton, J.L., Nielsen, J., et al. (2018). Negative cognitive style interacts with negative life events to predict ﬁrst onset of a major depressive episode in adolescence via hopelessness. *Journal of Abnormal Psychology., 127*, 1-11. |
| [Maciejewski, P. K., & Mazure, C. M. (2006). Fear of Criticism and Rejection Mediates an Association Between Childhood Emotional Abuse and Adult Onset of Major Depression. *Cognitive Therapy and Research, 30*(1), 105-122. https://doi.org/10.1007/s10608-006-9012-2](https://doi.org/10.1007/s10608-006-9012-2) |
| MacMillan, H.L., Fleming, J.E., Streiner, D.L., Lin, E., Boyle, M.H., Jamieson, E., et al. (2001). Childhood abuse and lifetime psychopathology in a community sample. *American Journal of Psychiatry, 158*(11), 1878-83. |
| [Mall, S., Mortier, P., Taljaard, L., Roos, J., Stein, D. J., & Lochner, C. (2018). The relationship between childhood adversity, recent stressors, and depression in college students attending a South African university. *BMC Psychiatry, 18*(1), 63. https://doi.org/10.1186/s12888-017-1583-9](https://doi.org/10.1186/s12888-017-1583-9) |
| [Malykhin, N. V., Carter, R., Seres, P., & Coupland, N. J. (2010). Structural changes in the hippocampus in major depressive disorder: Contributions of disease and treatment. *Journal of Psychiatry & Neuroscience, 35*(5), 337–343. https://doi.org/10.1503/jpn.100002](https://doi.org/10.1503/jpn.100002) |
| Mannarino, A. P., Cohen, J. A., Smith, J. A., & Moore-Motily, S. (1991). Six-and twelve-month follow-up of sexually abused girls. *Journal of Interpersonal Violence, 6*(4), 494-511. |
| [Mansbach-Kleinfeld, I., Ifrah, A., Apter, A., & Farbstein, I. (2015). Child sexual abuse as reported by Israeli adolescents: Social and health related correlates. *Child Abuse & Neglect, 40*, 68–80. https://doi.org/10.1016/j.chiabu.2014.11.014](https://doi.org/10.1016/j.chiabu.2014.11.014) |
| [McCutcheon, V. V., Heath, A. C., Nelson, E. C., Bucholz, K. K., Madden, P. A., & Martin, N. G. (2009). Accumulation of trauma over time and risk for depression in a twin sample. *Psychological Medicine, 39*(3), 431-441. https://doi.org/10.1017/S0033291708003759](https://doi.org/10.1017/S0033291708003759) |
| Mchichi Alami, K., & Kadri, N. (2004). Moroccan women with a history of child sexual abuse and its long-term repercussions: A population-based epidemiological study. *Archives of Women’s Mental Health, 7*(4), 237-42. |
| McLeer, S.V., Callaghan, M., Henry, D., & Wallen, J. (1994). Psychiatric disorders in sexually abused children. *Journal of the American Academy of Child and Adolescent Psychiatry, 33*, 313-319. |
| [Mikaeili, N., Barahmand, U., & Abdi, R. (2013). The prevalence of different kinds of child abuse and the characteristics that differentiate abused from nonabused male adolescents. *Journal of Interpersonal Violence, 28*(5), 975-996. https://doi.org/10.1177/0886260512459377](https://doi.org/10.1177/0886260512459377) |
| Miller, J.M., Kinnally, E.L., Ogden, R.T., Oquendo, M.A., Mann, J.J., & Parsey, R.V. (2009). Reported childhood abuse is associated with low serotonin transporter binding in vivo in major depressive disorder. *Synapse, 63*, 565–73. |
| Molnar, B. E., Buka, S. L., & Kessler, R. C. (2001). Child sexual abuse and subsequent psychopathology: Results from the National Comorbidity *Journal of Public Health, 91*(5), 753–760. |
| Morais, H.B., Alexander, A.A., Fix, R.L., & Burkhart, B.R. ((2018). Childhood sexual abuse in adolescents adjudicated for sexual offenses: mental health consequences and sexual offending behaviors. *Sexual Abuse, 30*, 23-42. |
| Mullen, P. E., Martin, J. L., Anderson, J. C., Romans, S. E., & Herbison, G. P. (1993). Childhood sexual abuse and mental health in adult life. *The British Journal of Psychiatry, 163*, 721–732. |
| Mullen, P. E., Martin, J. L., Anderson, J. C., Romans, S. E., & Herbison, G. P. (1996). The long-term impact of the physical, emotional, and sexual community study. *Child Abuse & Neglect, 20*(1), 7–21. |
| Münzer, A., Fegert, J. M., & Goldbeck, L. (2016). Psychological symptoms of sexually victimized children and adolescents compared with other maltreatment subtypes. *J Child Sexual Abuse, 25*, 326–346. doi: 10.1080/10538712.2016.1137667 |
| [Murphy, M. L., Carballedo, A., Fagan, A. J., Morris, D., Fahey, C., Meaney, J., & Frodl, T. (2012). Neurotrophic tyrosine kinase polymorphism impacts white matter connections in patients with major depressive disorder. *Biological Psychiatry, 72*(8), 663–670. https://doi.org/10.1016/j.biopsych.2012.04.015](https://doi.org/10.1016/j.biopsych.2012.04.015) |
| [Nasreen, H. E., Alam, M. A., & Edhborg, M. (2016). Prevalence and associated factors of depressive symptoms among disadvantaged adolescents: population-based study in Bangladesh. *Journal of Child and Adolescent Psychiatric Nursing, 29*(3), 135–144. https://doi.org/10.1111/jcap.12150](https://doi.org/10.1111/jcap.12150) |
| Nduna, M., Jewkes, R.K., Dunkle, K.L., Shai, N.P.J., & Colman, I. (2013). Prevalence and factors associated with depressive symptoms among young women and men in the Eastern Cape Province, South Africa. *Journal of Child & Adolescent Mental Health,* *25*(1), 43-54. |
| Nelson, E. C., Heath, A. C., Madden, P. A., Cooper, M. L., Dinwiddie, S. H., Bucholz, K. K., ... Martin, N. G. (2002). Association between self-reported abuse and adverse psychosocial outcomes: Results from a twin study. *Archives of General Psychiatry, 59*(2), 139–145. |
| [Ng, R. M., Bhugra, D., Mcmanus, F., & Fennell, M. (2011). Filial piety as a protective factor for depression in survivors of childhood abuse. *International Review of Psychiatry, 23*(1), 100–112. https://doi.org/10.3109/09540261.2010.544645](https://doi.org/10.3109/09540261.2010.544645) |
| Nicolaidis, C., Curry, M., McFarland, B.H., & Gerrity, M.S. (2004). Violence, mental health, and physical symptoms in an academic internal medicine practice. *Journal of General Internal Medicine,* *19*(8), 819-27. |
| [Novelo, M., Von Gunten, A., Jardim, G. B. G., Spanemberg, L., de Lima Argimon, I. I. II., & Nogueira, E. L. (2018). Effects of childhood multiple maltreatment experiences on depression of socioeconomic disadvantaged elderly in Brazil. *Child Abuse & Neglect, 79*, 350-357. https://doi.org/10.1016/j.chiabu.2018.02.013](https://doi.org/10.1016/j.chiabu.2018.02.013) |
| Olsson G. (1999). Violence in the lives of suicidal adolescents: a comparison between three matched groups of suicide attempting, depressed, and nondepressed high school students. *Int J Adolesc Med Health, 11*, 369-379. |
| [Opel, N., Redlich, R., Zwanzger, P., Grotegerd, D., Arolt, V., Heindel, W., ... Dannlowski, U. (2014). Hippocampal atrophy in major depression: a function of childhood maltreatment rather than diagnosis? *Neuropsychopharmacology, 39*(12), 2723–2731. https://doi.org/10.1038/npp.2014.145](https://doi.org/10.1038/npp.2014.145) |
| [Opel, N., Zwanzger, P., Redlich, R., Grotegerd, D., Dohm, K., Arolt, V., ... Dannlowski, U. (2016). Differing brain structural correlates of familial and environmental risk for major depressive disorder revealed by a combined VBM/pattern recognition approach. *Psychological Medicine, 46*(2), 277–290. https://doi.org/10.1017/S0033291715001683](https://doi.org/10.1017/S0033291715001683) |
| Pantle, M.L., & Oegema, S.L. (1990). Sexual abuse and depression in an adolescent female inpatient population. *Journal of Psychology and Christianity, 9*, 55-63. |
| Paquette, G., Tourigny, M., Baril, K., Joly, J., & Seguin, M. (2017). Childhood maltreatment and mental health problems in adulthood: A national study of women in Quebec. *Sante Mentale au Quebec, 42*(1), 43–63. |
| Pham, T. S., Qi, H., Chen, D., Chen, H., & Fan, F. (2021). Prevalences of and correlations between childhood trauma and depressive symptoms, anxiety symptoms, and suicidal behavior among institutionalized adolescents in Vietnam. *Child Abuse Negl., 115*, 105022. doi: 10.1016/j.chiabu.2021.105022 |
| [Pickles, A., Aglan, A., Collishaw, S., Messer, J., Rutter, M., & Maughan, B. (2010). Predictors of suicidality across the life span: The Isle of wight study. *Psychological Medicine, 40*(9), 1453–1466. https://doi.org/10.1017/s0033291709991905](https://doi.org/10.1017/s0033291709991905) |
| Plaza, A., Garcia-Esteve, L., Torres, A., Ascaso, C., Gelabert, E., Luisa Imaz, M., et al. (2012). Childhood physical abuse as a common risk factor for depression and thyroid dysfunction in the earlier postpartum. *Psychiatry Research, 200*(2-3), 329-35. |
| Qu, G. B. (2022). *Study on the effect of positive and negative childhood experiences on anxiety and depression of primary and middle school students and the mediating effect of inflammatory burden*. Anhui Medical University, Hefei. |
| [Raheel, H. (2015). Depression and associated factors among adolescent females in Riyadh, Kingdom of Saudi Arabia, a cross-sectional study. *International Journal of Preventative Medicine, 6*(1), 90. https://doi.org/10.4103/2008-7802.165156](https://doi.org/10.4103/2008-7802.165156) |
| Ramos, B.M., Carlson, B.E., & McNutt, L. (2004). Lifetime abuse, mental health, and African American women. *Journal of Family Violence*, *19*(3), 153-64. |
| [Remigio-Baker, R. A., Hayes, D. K., & Reyes-Salvail, F. (2014). Adverse childhood events and current depressive symptoms among women in Hawaii: 2010 BRFSS, Hawaii. *Maternal and Child Health Journal, 18*(10), 2300–2308. https://doi.org/10.1007/s10995-013-1374-y](Remigio-Baker,%20R.%20A.,%20Hayes,%20D.%20K.,%20&%20Reyes-Salvail,%20F.%20(2014).%20Adverse%20childhood%20events%20and%20current%20depressive%20symptoms%20among%20women%20in%20Hawaii:%202010%20BRFSS,%20Hawaii.%20Maternal%20and%20Child%20Health%20Journal,%2018(10),%202300–2308.%20https://doi.org/10.1007/s10995-013-1374-y) |
| [Ritchie, K., Jaussent, I., Stewart, R., Dupuy, A. M., Courtet, P., Anceliln, M. L., & Malafosse, A. (2009). Association of adverse childhood environment and 5-HTTLPR genotype with late-life depression. *Journal of Clinical Psychiatry, 70*(9), 1281–1288. https://doi.org/10.4088/JCP.08m04510](https://doi.org/10.4088/JCP.08m04510) |
| Rivera-Rivera, L., Rivera-Hernandez, P., Perez-Amezcua, B., Leyva-Lopez, A., & Castro, F. (2015). [Individual and family factors associated with depressive symptomatology in adolescents from public schools of Mexico]. *Salud Publica de Mexico, 57*(3), 219–226. |
| Rizzo, C.J., Esposito-Smythers, C., Spirito, A., & Thompson, A. (2010). Psychiatric and cognitive functioning in adolescent inpatients with histories of dating violence victimization. *Journal of Aggression, Maltreatment & Trauma, 19*, 565-583. |
| [Rohde, P., Ichikawa, L., Simon, G. E., Ludman, E. J., Linde, J. A., Jeffery, R. W., ... Operskalski, B. H. (2008). Associations of child sexual and physical abuse with obesity and depression in middle-aged women. *Child Abuse & Neglect, 32*(9), 878–887. https://doi.org/10.1016/j.chiabu.2007.11.004](https://doi.org/10.1016/j.chiabu.2007.11.004) |
| Rorty, M., Yager, J., & Rossotto, E. (1994). Childhood sexual, physical, and psychological abuse and their relationship to comorbid psychopathology in bulimia nervosa. *International Journal of Eating Disorders*, *16*(4), 31734. |
| [Roustit, C., Renahy, E., Guernec, G., Lesieur, S., Parizot, I., & Chauvin, P. (2009). Exposure to interparental violence and psychosocial maladjustment in the adult life course: Advocacy for early prevention. *Journal of Epidemiology and Community Health, 63*(7), 563–568. https://doi.org/10.1136/jech.2008.077750](https://doi.org/10.1136/jech.2008.077750) |
| [Rubino, I. A., Nanni, R. C., Pozzi, D. M., & Siracusano, A. (2009). Early adverse experiences in schizophrenia and unipolar depression. *The Journal of Nervous and Mental Disease, 197*(1), 65–68. https://doi.org/10.1097/NMD.0b013e3181925342](https://doi.org/10.1097/NMD.0b013e3181925342) |
| Sadowski, H., Trowell, J., Kolvin, I., Weeramanthri, T., Berelowitz, M., & Gilbert, L. H. (2003). Sexually abused girls: patterns of psychopathology and exploration of risk factors. *Eur Child Adolesc Psychiatry, 12*, 221–230. doi: 10.1007/s00787-003-0334-2 |
| [Satinsky, E. N., Kakuhikire, B., Baguma, C., Rasmussen, J. D., Ashaba, S., Cooper-Vince, C. E., Perkins, J. M., Kiconco, A., Namara, E. B., Bangsberg, D. R., & Tsai, A. C. (2021). Adverse childhood experiences, adult depression, and suicidal ideation in rural Uganda: A cross-sectional, population-based study. *PLoS Medicine, 18*(5), e1003642. https://doi.org/10.1371/journal.pmed.1003642](https://doi.org/10.1371/journal.pmed.1003642) |
| Scott, K. D. (1992). Childhood sexual abuse: Impact on a community’s mental health status. *Child Abuse & Neglect, 16*(2), 285–295. |
| Saunders, B.E., Villeponteaux, L.A., Lipovsky, J.A., Kilpatrick, D.G., & Veronen, L.J. (1992). Child sexual assault as a risk factor for mental disorders among women: A community survey. *Journal of Interpersonal Violence,* *7*(2), 189-204. |
| Sedney, M.A., & Brooks, B. (1984). Factors associated with a history of childhood sexual experience in a nonclinical female population. *Journal of the American Academy of Child Psychiatry,* *23*(2), 215-8. |
| Shah, S. M., Nowshad, G., Dhaheri, F. A., Al-Shamsi, M. H., Al-Ketbi, A. M., Galadari. A., et al. (2021). Child maltreatment and neglect in the United Arab Emirates and relationship with low self-esteem and symptoms of depression. *Int Rev Psychiatry, 33*, 326–36. doi: 10.1080/09540261.2021.1895086 |
| Silverman, A.B., Reinherz, H.Z., & Giaconia, R.M. (1996). The long-term sequelae of child and adolescent abuse: A longitudinal community study. *Child Abuse & Neglect*, *20*(8), 709-23. |
| [Skeen, S., Macedo, A., Tomlinson, M., Hensels, I. S., & Sherr, L. (2016). Exposure to violence and psychological well-being over time in children affected by HIV/AIDS in South Africa and Malawi. *AIDS Care, 28* Suppl 1, 16-25. https://doi.org/10.1080/09540121.2016.1146219](https://doi.org/10.1080/09540121.2016.1146219) |
| [Sonnby, K., Aslund, C., Leppert, J., & Nilsson, K. W. (2011). Symptoms of ADHD and depression in a large adolescent population: Co-occurring symptoms and associations to experiences of sexual abuse. *Nordic Journal of Psychiatry, 65*(5), 315–322. https://doi.org/10.3109/08039488.2010.545894](https://doi.org/10.3109/08039488.2010.545894) |
| [Springer, K. W., Sheridan, J., Kuo, D., & Carnes, M. (2007). Long-term physical and mental health consequences of childhood physical abuse: Results from a large population-based sample of men and women. *Child Abuse & Neglect, 31*(5), 517–530. https://doi.org/10.1016/j.chiabu.2007.01.003](https://doi.org/10.1016/j.chiabu.2007.01.003) |
| Stuart, G. W., Laraia, M. T., Ballenger, J. C., & Lydiard, R. B. (1990). Early family experiences of women with bulimia and depression. *Archives of Psychiatric Nursing, 4*(1), 43–52. |
| Subica, A.M. (2013). Psychiatric and physical sequelae of childhood physical and sexual abuse and forced sexual trauma among individuals with serious mental illness. *Journal of Traumatic Stress,* *26*(5), 58896. |
| [Subramaniam, M., Abdin, E., Seow, E., Vaingankar, J. A., Shafie, S., Shahwan, S., Lim, M., Fung, D., James, L., Verma, S., & Chong, S. A. (2020). Prevalence, socio-demographic correlates and associations of adverse childhood experiences with mental illnesses: Results from the Singapore mental health study. *Child Abuse & Neglect, 103*, 104447. https://doi.org/10.1016/j.chiabu.2020.104447](https://doi.org/10.1016/j.chiabu.2020.104447) |
| [Sugaya, L., Hasin, D. S., Olfson, M., Lin, K. H., Grant, B. F., & Blanco, C. (2012). Child physical abuse and adult mental health: A national study. *Journal of Traumatic Stress, 25*(4), 384–392. https://doi.org/10.1002/jts.21719](https://doi.org/10.1002/jts.21719) |
| [Suzuki, A., Poon, L., Papadopoulos, A. S., Kumari, V., & Cleare, A. J. (2014). Long term effects of childhood trauma on cortisol stress reactivity in adulthood and relationship to the occurrence of depression. *Psychoneuroendocrinology, 50*, 289–299. https://doi.org/10.1016/j.psyneuen.2014.09.007](https://doi.org/10.1016/j.psyneuen.2014.09.007) |
| [Taillieu, T. L., Brownridge, D. A., Sareen, J., & Afifi, T. O. (2016). Childhood emotional maltreatment and mental disorders: Results from a nationally representative adult sample from the United States. *Child Abuse & Neglect, 59*, 1–12. https://doi.org/10.1016/j.chiabu.2016.07.005](https://doi.org/10.1016/j.chiabu.2016.07.005) |
| [Tatham, E. L., Ramasubbu, R., Gaxiola-Valdez, I., Cortese, F., Clark, D., Goodyear, B., Foster, J., & Hall, G. B. (2016). White matter integrity in major depressive disorder: Implications of childhood trauma, 5-HTTLPR and BDNF polymorphisms. *Psychiatry Research: Neuroimaging, 253*, 15–25. https://doi.org/10.1016/j.pscychresns.2016.04.014](https://doi.org/10.1016/j.pscychresns.2016.04.014) |
| Tietjen, G.E., Brandes, J.L., Peterlin, B., Eloff, A., Dafer, R.M., Stein, M.R., et al. (2010). Childhood maltreatment and migraine (Part I). Prevalence and adult revictimization: A multicenter headache clinic survey. *Headache,* *50*(1), 20-31. |
| [Treadway, M. T., Grant, M. M., Ding, Z., Hollon, S. D., Gore, J. C., & Shelton, R. C. (2009). Early adverse events, HPA activity and rostral anterior cingulate volume in MDD. *PloS One, 4*(3), e4887. https://doi.org/10.1371/journal.pone.0004887](https://doi.org/10.1371/journal.pone.0004887) |
| [Turner, S., Taillieu, T., Cheung, K., & Afifi, T. O. (2017). The relationship between childhood sexual abuse and mental health outcomes among males: Results from a nationally representative United States sample. *Child Abuse & Neglect, 66*, 64–72. https://doi.org/10.1016/j.chiabu.2017.01.018](https://doi.org/10.1016/j.chiabu.2017.01.018) |
| [Ugwu, I. D., Amico, F., Carballedo, A., Fagan, A. J., & Frodl, T. (2015). Childhood adversity, depression, age and gender effects on white matter microstructure: a DTI study. *Brain Structure and Function, 220*(4), 1997–2009. https://doi.org/10.1007/s00429-014-0769-x](https://doi.org/10.1007/s00429-014-0769-x) |
| [Van Assche, L., de Ven, L. V., Vandenbulcke, M., & Luyten, P. (2020). Ghosts from the past? The association between childhood interpersonal trauma, attachment and anxiety and depression in late life. *Aging & Mental Health, 24*(6), 898-905. https://doi.org/10.1080/13607863.2019.1571017](https://doi.org/10.1080/13607863.2019.1571017) |
| Wahab, S., Tan, S.M.K., Marimuthu, S., Razali, R., & Muhamad, N.A. (2013). Young female survivors of sexual abuse in Malaysia and depression: what factors are associated with better outcome? *Asia-Paciﬁc Psychiatry, 5*, 95-102. |
| Wainwright, N. W., & Surtees, P. G. (2002). Childhood adversity, gender and depression over the life-course. *Journal of Affective Disorders, 72*(1), 33–44. |
| Walker, E.A., Katon, W.J., Hansom, J., Harrop-Griffiths, J., Holm, L., Jones, M.L., et al. (1992). Medical and psychiatric symptoms in women with childhood sexual abuse. *Psychosomatic Medicine,* *54*(6), 658-64. |
| Walker, E., Katon, W., Harrop-Griffiths, J., Holm, L., Russo, J., & Hickok, L. R. (1988). Relationship of chronic pelvic pain to psychiatric diagnoses and childhood sexual abuse. *American Journal of Psychiatry, 145*(1), 75-80. |
| Walton, G., Co, S.J., Milloy, M.J., Qi, J., Kerr, T., & Wood, E. (2011). High prevalence of childhood emotional, physical and sexual trauma among a Canadian cohort of HIV-seropositive illicit drug users. *AIDS Care,* *23*(6), 714-21. |
| Watson, S., Owen, B. M., Gallagher, P., Hearn, A. J., Young, A. H., & Ferrier, I. N. (2007). Family history, early adversity and the hypothalamic-pituitary-adrenal (HPA) axis: Mediation of the vulnerability to mood disorders. *Neuropsychiatric Disease and Treatment, 3*(5), 647–653. |
| Webster, J.J., & Palmer, R.L. (2000). The childhood and family background of women with clinical eating disorders: a comparison with women with major depression and women without psychiatric disorder. *Psychological Medicine, 30*, 53–60. |
| Weeramanthri, T., Berelowitz, M., Sadowski, H., Trowell, J., & Kolvin, I. (2003). Sexually abused girls: patterns of psychopathology and exploration of risk factors. *European Child and Adolescent Psychiatry, 12*, 221-230. |
| [Wessel, I., Meeren, M., Peeters, F., & Arntz Merckelbach, A. H. (2001). Correlates of autobiographical memory specificity: The role of depression, anxiety and childhood trauma. *Behaviour Research and Therapy, 39*(4), 409–421. https://doi.org/10.1016/S0005-7967(00)00011-5](https://doi.org/10.1016/S0005-7967(00)00011-5) |
| [Widom, C. S., DuMont, K., & Czaja, S. J. (2007). A prospective investigation of major depressive disorder and comorbidity in abused and neglected children grown up. *Archives of General Psychiatry, 64*(1), 49–56. https://doi.org/10.1001/archpsyc.64.1.49](https://doi.org/10.1001/archpsyc.64.1.49) |
| Wilsnack, S.C., Vogeltanz, N.D., Klassen, A.D., & Harris, T.R. (1997). Childhood sexual abuse and women's substance abuse: National survey findings. *Journal of Studies on Alcohol and Drugs,* *58*(3), 264-71. |
| Wilson, S., Vaidyanathan, U., Miller, M.B., Mcgue, M., & Iacono, W.G. (2014). Premorbid risk factors for major depressive disorder: are they associated with early onset and recurrent course? *Developmental Psychopathology, 26*, 1477-1493. |
| Windle, M., Windle, R.C., Scheidt, D.M., & Miller, G.B. (1995). Physical and sexual abuse and associated mental disorders among alcoholic inpatients. *American Journal of Psychiatry*, *152*(9), 1322-8. |
| [Wingenfeld, K., Kuehl, L. K., Boeker, A., Schultebraucks, K., Schulz, A., Stenzel, J., Spitzer, C., & Otte, C. (2017). Are adverse childhood experiences and depression associated with impaired glucose tolerance in females? An experimental study. *Journal of Psychiatric Research, 95*, 60–67. https://doi.org/10.1016/j.jpsychires.2017.07.028](https://doi.org/10.1016/j.jpsychires.2017.07.028) |
| [Wingenfeld, K., Kuffel, A., Uhlmann, C., Terfehr, K., Schreiner, J., Kuehl, L. K., ... Spitzer, C. (2013). Effects of noradrenergic stimulation on memory in patients with major depressive disorder. *Stress, 16*(2), 191–201. https://doi.org/10.3109/10253890.2012.708951](https://doi.org/10.3109/10253890.2012.708951) |
| Wise, L.A., Zierler, S., Krieger, N., & Harlow, B.L. (2001). Adult onset of major depressive disorder in relation to early life violent victimisation: A case-control study. *Lancet*, *358*(9285), 881-7. |
| Xiao, Y., Ran, H., Fang, D., Che, Y., Peng, J., Sun, H., et al. (2022). Childhood maltreatment and depressive disorders in Chinese children and adolescents: A population-based case-control study. *Asian J Psychiatry, 78*, 103312. doi: 10.1016/j.ajp.2022.103312 |
| Xiong, N., Liu, Q., Lv, X., Leonhart, R., Tian, H., Wei, J., Zhang, K., Zhu, G., Chen, Q., Wang, G., Wang, X., Zhang, N., Huang, Y., Si, T., & Yu, X. (2020). Demographic and psychosocial variables could predict the occurrence of major depressive disorder, but not the severity of depression in patients with first-episode major depressive disorder in china. *Journal of Affective Disorders, 274*, 103–111. https://doi.org/10.1016/j.jad.2020.05.065. |
| [Yang, S., Cheng, Y., Mo, Y., Bai, Y., Shen, Z., Liu, F., ... Sun, X. (2017). Childhood maltreatment is associated with gray matter volume abnormalities in patients with first-episode depression. *Psychiatry Research: Neuroimaging, 268*, 27–34. https://doi.org/10.1016/j.pscychresns.2017.07.005](https://doi.org/10.1016/j.pscychresns.2017.07.005) |
| [Yen, C. F., Yang, M. S., Chen, C. C., Yang, M. J., Su, Y. C., Wang, M. H., ... Lan, C. M. (2008). Effects of childhood physical abuse on depression, problem drinking and perceived poor health status in adolescents living in rural Taiwan. *Psychiatry and Clinical Neurosciences, 62*(5), 575–583. https://doi.org/10.1111/j.1440-1819.2008.01836.x](https://doi.org/10.1111/j.1440-1819.2008.01836.x) |
| Yin, H., Galfalvy, H., Zhang, B., Tang, W., Xin, Q., Li, E., Xue, X., Li, Q., Ye, J., Yan, N., & Mann, J. J. (2020). Interactions of the GABRG2 polymorphisms and childhood trauma on suicide attempt and related traits in depressed patients. *Journal of Affective Disorders, 266*, 447–455. https://doi.org/10.1016/j.jad.2020.01.126. |
| Young, E.A., Abelson, J.L., Curtis, G.C., & Nesse, R.M. (1997). Childhood adversity and vulnerability to mood and anxiety disorders. *Depression and Anxiety, 5*,66–72. |
| Yu, T., Hu, J., Zhang, W., Zhang, L., & Zhao, J. (2022). Psychological maltreatment and depression symptoms among Chinese adolescents: A multiple mediation model. *Child Abuse Negl., 134*, 105940. doi: 10.1016/j.chiabu.2022.105940 |
| Zavaschi, M.L., Graeff, M.E., Menegassi, M.T., Mardini, V., Pires, D.W., Carvalho, R.H., et al. (2006). Adult mood disorders and childhood psychological trauma. *Revista Brasileira de Psiquiatria, 28*, 184–90. |
| [Zelikovsky, N., & Lynn, S. J. (2002). Childhood Psychological and Physical Abuse: Psychopathology, Dissociation, and Axis I Diagnosis. *Journal of Trauma & Dissociation, 3*(3), 27-58. https://doi.org/10.1300/J229v03n03_03](https://doi.org/10.1300/J229v03n03_03) |
| Zhao, H., Wei, T., Li, X., & Ba, T. (2018). Early life adversity induced third ventricular enlargement in young adult male patients suffered from major depressive disorder: A study of brain morphology. *Folia Morphologica, 77*(3), 428–433. https://doi.org/10.5603/FM.a2017.0113. |
| Zheng (2016). Study in Guangzhou, China from guangzhou zun Lao recreation association; listed in Wang et al. (2023) |
| Zuravin SJ, Fontanella C. (1999). The relationship between child sexual abuse and major depression among low-income women: A function of growing up experiences? *Child Maltreatment*, *4*(1), 3-12. |

| **References (severity studies)** |
| --- |
| [Aguilera, M., Arias, B., Wichers, M., Barrantes-Vidal, N., Moya, J., Villa, H., ... Fañanás, L. (2009). Early adversity and 5-HTT/BDNF genes: New evidence of gene-environment interactions on depressive symptoms in a general population. *Psychological Medicine, 39*(9), 1425–1432. https://doi.org/10.1017/S0033291709005248](https://doi.org/10.1017/S0033291709005248) |
| [Akbaba Turkoglu, S., Essizoglu, A., Kosger, F., & Aksaray, G. (2015). Relationship between dysfunctional attitudes and childhood traumas in women with depression. The International *Journal of Social Psychiatry, 61*(8), 796–801. https://doi.org/10.1177/0020764015585328](https://doi.org/10.1177/0020764015585328) |
| Allen, J. G., Coyne, L., & Huntoon, J. (1998). Trauma pervasively elevates brief symptom inventory profiles in inpatient women. *Psychological Reports, 83*, 499–513. https://doi.org/10.2466/pr0.1998.83.2.499 |
| Allen, B. (2008). An analysis of the impact of diverse forms of childhood psychological maltreatment on emotional adjustment in early adulthood. *Child Maltreatment*, *13*(3), 307-12. |
| [Arata, C. M., Langhinrichsen-Rohling, J., Bowers, D., & O’Farrill-Swails, L. (2005). Single versus multi-type maltreatment: An examination of the long-term effects child abuse. *Journal of Aggression, 11*(4), 29–52. https://doi.org/10.1300/J146v11n04_02.](https://doi.org/10.1300/J146v11n04_02) |
| Arslan, G. (2015). Relationship between childhood psychological maltreatment, resilience, depression, and negative self-concept. *Yeni Symposium, 53*(4), 3–10 |
| [Auslander, W., Sterzing, P., Threlfall, J., Gerke, D., & Edmond, T. (2016). Childhood abuse and aggression in adolescent girls involved in child welfare: The role of depression and posttraumatic stress. *Journal of Child & Adolescent Trauma, 9*(4), 359–368. https://doi.org/10.1007/s40653-016-0090-3](https://doi.org/10.1007/s40653-016-0090-3) |
| Bagley, C., Wood, M., & Young, L. (1994). Victim to abuser: Mental health and behavioral sequels of child sexual abuse in a community survey of young adult males. *Child Abuse & Neglect,* *18*(8), 683-97. |
| [Bailer, J., Witthöft, M., Wagner, H., Mier, D., Diener, C., & Rist, F. (2014). Childhood maltreatment is associated with depression but not with hypochondriasis in later life. *Journal of Psychosomatic Research, 77*(2), 104–108. https://doi.org/10.1016/j.jpsychores.2014.06.004](https://doi.org/10.1016/j.jpsychores.2014.06.004) |
| [Balsam, K. F., Lehavot, K., Beadnell, B., & Circo, E. (2010). Childhood abuse and mental health indicators among ethnically diverse lesbian, gay, and bisexual adults. *Journal of Consulting and Clinical Psychology, 78*(4), 459–468. https://doi.org/10.1037/a0018661](https://doi.org/10.1037/a0018661) |
| [Banducci, A. N., Hoffman, E., Lejuez, C. W., & Koenen, K. C. (2014). The relationship between child abuse and negative outcomes among substance users: Psychopathology, health, and comorbidities. *Addictive Behaviors, 39*(10), 1522–1527. https://doi.org/10.1016/j.addbeh.2014.05.023](https://doi.org/10.1016/j.addbeh.2014.05.023) |
| [Basu, A., Levendosky, A. A., & Lonstein, J. S. (2013). Trauma sequelae and cortisol levels in women exposed to intimate partner violence. *Psychodynamic Psychiatry, 41*(2), 247–275. https://doi.org/10.1521/pdps.2013.41.2.247](https://doi.org/10.1521/pdps.2013.41.2.247) |
| Berenbaum, H., Valera, E.M., & Kerns, J.G. (2003). Psychological trauma and schizotypal symptoms. *Schizophrenia Bulletin,* *29*(1), 143-52. |
| [Blain, L. M., Muench, F., Morgenstern, J., & Parsons, J. T. (2012). Exploring the role of child sexual abuse and posttraumatic stress disorder symptoms in gay and bisexual men reporting compulsive sexual behavior. *Child Abuse & Neglect, 36*(5), 413–422. https://doi.org/10.1016/chiabu.2012.03.003](https://doi.org/10.1016/chiabu.2012.03.003) |
| Bohn, D., Bernardy, K., Wolfe, F., & Häuser, W. (2013). The association among childhood maltreatment, somatic symptom intensity, depression, and somatoform dissociative symptoms in patients with fibromyalgia syndrome: A single-center cohort study. *Journal of Trauma & Dissociation*, *14*(3), 342-58. |
| Brodsky, B.S., Cloitre, M., & Dulit, R.A. (1995). Relationship of dissociation to self-mutilation and childhood abuse in borderline personality disorder. *American Journal of Psychiatry,* *152*(12), 1788-92. |
| [Brown, S., Fite, P. J., Stone, K., & Bortolato, M. (2016). Accounting for the associations between child maltreatment and internalizing problems: The role of alexithymia. *Child Abuse & Neglect, 52*, 20–28. https://doi.org/10.1016/j.chiabu.2015.12.008](https://doi.org/10.1016/j.chiabu.2015.12.008) |
| [Burns, E. E. (2012). Childhood emotional maltreatment, depression, and eating disorder symptomatology [Doctoral dissertation, University of Georgia, Athens, USA]. Retrieved from https://athenaeum.libs.uga.edu/handle/10724/28212](https://athenaeum.libs.uga.edu/handle/10724/28212) |
| [Caldwell, J. G., Shaver, P. R., Li, C. S., & Minzenberg, M. J. (2011). Childhood maltreatment, adult attachment, and depression as predictors of parental self-efficacy in at-risk mothers. *Journal of Aggression, Maltreatment & Trauma, 20*(6), 595–616. https://doi.org/10.1080/10926771.2011.595763](https://doi.org/10.1080/10926771.2011.595763) |
| Calvete, E. (2014). Emotional abuse as a predictor of early maladaptive schemas in adolescents: Contributions to the development of depressive and social anxiety symptoms. *Child Abuse & Neglect, 38*(4), 735-746. |
| Cao, H., Liang, Y., Li, X., Zhu, L., Wu, L., Liu, H., Jiang, L., Zhou, N., & Zhang, J. (2020). Childhood maltreatment and affective symptoms and severity of drug addiction among Chinese male drug users: Variable-centered and person-centered approaches. *Journal of Aggression, Maltreatment & Trauma.* https://doi.org/10.1080/10926771.2020.1796874 |
| Carlson, E.B., Dalenberg, C.J., Armstrong, .JG., Daniels, J.W., Loewenstein, R.J., & Roth, D. (2001). Multivariate prediction of posttraumatic symptoms in psychiatric inpatients. *Journal of Traumatic Stress,* *14*(3), 549-67. |
| [Chen, L., Xu, L., You, W., Zhang, X., & Ling, N. (2017). Prevalence and associated factors of alexithymia among adult prisoners in China: A cross-sectional study. *BMC Psychiatry, 17*(1), 287. https://doi.org/10.1186/s12888-017-1443-7](https://doi.org/10.1186/s12888-017-1443-7) |
| [Choi, K. W., Sikkema, K. J., Velloza, J., Marais, A., Jose, C., Stein, D. J., Watt, M. H., & Joska, J. A. (2015). Maladaptive coping mediates the influence of childhood trauma on depression and PTSD among pregnant women in South Africa. *Archives of Women's Mental Health, 18*(5), 731–738. https://doi.org/10.1007/s00737015-0501-8](https://doi.org/10.1007/s00737015-0501-8) |
| Coates, A. A., & Messman-Moore, T. L. (2014). A structural model of mechanisms predicting depressive symptoms in women following childhood psychological maltreatment. *Child Abuse & Neglect, 38*(1), 103-113. |
| [Cohen, J. R., Menon, S. V., Shorey, R. C., Le, V. D., & Temple, J. R. (2017). The distal consequences of physical and emotional neglect in emerging adults: A person-centered, multi-wave, longitudinal study. *Child Abuse & Neglect, 63*, 151–161. https://doi.org/10.1016/j.chiabu.2016.11.030](https://doi.org/10.1016/j.chiabu.2016.11.030) |
| Courtney, E. A., Johnson, J. G., & Alloy, L. B. (2008). Associations of childhood maltreatment with hopelessness and depression among adolescent primary care patients. *International Journal of Cognitive Therapy, 1*(1), 4-17. |
| [Crow, T., Cross, D., Powers, A., & Bradley, B. (2014). Emotion dysregulation as a mediator between childhood emotional abuse and current depression in a low-income African-American sample. *Child Abuse & Neglect, 38*(10), 1590–1598. https://doi.org/10.1016/j.chiabu.2014.05.015](https://doi.org/10.1016/j.chiabu.2014.05.015) |
| Cui, L., Luo, X., & Xiao, J. (2011). The influence of childhood trauma on trait-depression and trait-anxiety: The mediation-specificity of schemas. *Acta Psychologica Sinica, 43*(10), 1163–1174. |
| [Dackis, M. N., Rogosch, F. A., Oshri, A., & Cicchetti, D. (2012). The role of limbic system irritability in linking history of childhood maltreatment and psychiatric outcomes in low-income, high-risk women: moderation by FK506 binding protein 5 haplotype. *Development and Psychopathology, 24*(4), 1237–1252. https://doi.org/10.1017/S0954579412000673](https://doi.org/10.1017/S0954579412000673) |
| [Day, D. M., Hart, T. A., Wanklyn, S. G., McCay, E., Macpherson, A., & Burnier, N. (2013). Potential mediators between child abuse and both violence and victimization in juvenile offenders. *Psychological Services, 10*(1), 1–11. https://doi.org/10.1037/a0028057](https://doi.org/10.1037/a0028057) |
| Dunkley, D.M., Masheb, R.M., & Grilo, C.M. (2010). Childhood maltreatment, depressive symptoms, and body dissatisfaction in patients with binge eating disorder: The mediating role of self-criticism. *International Journal of Eating Disorders,* *43*(3), 274-81. |
| [Dunlop, B. W., Hill, E., Johnson, B. N., Klein, D. N., Gelenberg, A. J., Rothbaum, B. O., ... Kocsis, J. H. (2015). Mediators of sexual functioning and marital quality in chronically depressed adults with and without a history of childhood sexual abuse. *The Journal of Sexual Medicine, 12*(3), 813–823. https://doi.org/10.1111/jsm.12727](https://doi.org/10.1111/jsm.12727) |
| [Ensink, K., Begin, M., Normandin, L., & Fonagy, P. (2016). Maternal and child reflective functioning in the context of child sexual abuse: pathways to depression and externalizing difficulties. European *Journal of Psychotraumatology, 7*, 30611. https://doi.org/10.3402/ejpt.v7.30611](https://doi.org/10.3402/ejpt.v7.30611) |
| [Gerke, C. K., Mazzeo, S. E., & Kliewer, W. (2006). The role of depression and dissociation in the relationship between childhood trauma and bulimic symptoms among ethnically diverse female undergraduates. *Child Abuse & Neglect, 30*(10), 1161–1172. https://doi.org/10.1016/j.chiabu.2006.03.010](https://doi.org/10.1016/j.chiabu.2006.03.010) |
| [Gibb, B. E., & Abela, J. R. (2008). Emotional abuse, verbal victimization, and the development of children’s negative inferential styles and depressive symptoms. *Cognitive Therapy and Research, 32*(2), 161–176. https://doi.org/10.1007/s10608-006-9106-x.](https://doi.org/10.1007/s10608-006-9106-x) |
| Gibb, B. E., Alloy, L. B., Abramson, L. Y., & Marx, B. P. (2003). Childhood maltreatment and maltreatment-specific inferences: A test of Rose and Abramson's (1992) extension of the hopelessness theory. *Cognition & Emotion, 17*(6), 917-931. |
| Gibb, B.E., Benas, J.S., Crossett, S.E., & Uhrlass, D.J. (2007). Emotional maltreatment and verbal victimization in childhood: Relation to adults' depressive cognitions and symptoms. *Journal of Emotional Abuse,* *7*(2), 59-73. |
| Goldsmith, R.E., & Freyd, J.J. (2005). Awareness for emotional abuse. *Journal of Emotional Abuse,* *5*(1), 95-12379. |
| [Goldstein, A. L., Faulkner, B., & Wekerle, C. (2013). The relationship among internal resilience, smoking, alcohol use, and depression symptoms in emerging adults transitioning out of child welfare. *Child Abuse & Neglect, 37*(1), 22–32. https://doi.org/10.1016/j.chiabu.2012.08.007](https://doi.org/10.1016/j.chiabu.2012.08.007) |
| [Goldstein, A. L., Vilhena-Churchill, N., Stewart, S. H., & Wekerle, C. (2012). Coping motives as moderators of the relationship between emotional distress and alcohol problems in a sample of adolescents involved with child welfare. *Advances in Mental Health, 11*(1), 67–75. https://doi.org/10.5172/jamh.2012.2750](https://doi.org/10.5172/jamh.2012.2750) |
| Gong, J., & Chan, R. C. K. (2018). Early maladaptive schemas as mediators between childhood maltreatment and later psychological distress among Chinese college students. *Psychiatry Research,* *259*, 493–500. https://doi.org/10.1016/j.psychres.2017.11.019. |
| Gong, J., Liu, J., Zhang, Y., & Luo, X. (2016). Incidence of neurological soft signs and the analysis of its relevant factors in college students with childhood abuse. *Chinese Mental Health Journal, 30*(6), 459–463. https://doi.org/10.3969/j.issn.1000–6729.2016.06.011. |
| [Gratz, K. L., Latzman, R. D., Tull, M. T., Reynolds, E. K., & Lejuez, C. W. (2011). Exploring the association between emotional abuse and childhood borderline personality features: The moderating role of personality traits. *Behavior Therapy, 42*(3), 493–508. https://doi.org/10.1016/j.beth.2010.11.003](https://doi.org/10.1016/j.beth.2010.11.003) |
| Groleau, P., Steiger, H., Bruce, K., Israel, M., Sycz, L., Ouellette, A.S., et al. (2012). Childhood emotional abuse and eating symptoms in bulimic disorders: An examination of possible mediating variables. *International Journal of Eating Disorders,* *45*(3), 326-32. |
| [Haapasalo, J., & Virtanen, T. (1999). Paths Between Childhood Emotional and Other Maltreatment and Psychiatric Problems in Criminal Offenders. *Journal of Emotional Abuse, 1*(4), 15-35. https://doi.org/10.1300/J135v01n04_0258](https://doi.org/10.1300/J135v01n04_0258) |
| Haj-Yahia, M.M., & de Zoysa, P. (2008). Rates and psychological effects of exposure to family violence among Sri Lankan university students. *Child Abuse & Neglect,* *32*(10), 994-1002. |
| [Hamilton, J. L., Potter, C. M., Olino, T. M., Abramson, L. Y., Heimberg, R. G., & Alloy, L. B. (2016). The temporal sequence of social anxiety and depressive symptoms following interpersonal stressors during adolescence. *Journal of Abnormal Child Psychology, 44*(3), 495–509. https://doi.org/10.1007/s10802-015-0049-0](https://doi.org/10.1007/s10802-015-0049-0) |
| Hankin, B.L. (2005). Childhood maltreatment and psychopathology: Prospective tests of attachment, cognitive vulnerability, and stress as mediating processes. *Cognitive Therapy and Research,* *29*(6), 645-71. |
| [Harding, H. G., Burns, E. E., & Jackson, J. L. (2012). Identification of child sexual abuse survivor subgroups based on early maladaptive schemas: Implications for understanding differences in posttraumatic stress disorder symptom severity. *Cognitive Therapy and Research, 36*(5), 560–575. https://doi.org/10.1007/s10608011-9385-8](https://doi.org/10.1007/s10608011-9385-8) |
| Heckman, C.J., & Westefeld, J.S. (2006). The relationship between traumatization and pain: What is the role of emotion? *Journal of Family Violence,* *21*(1), 63-73. |
| Hobfoll, S.E., Bansal, A., Schurg, R., Young, S., Pierce, C.A., Hobfoll, I., et al. (2002). The impact of perceived child physical and sexual abuse history on Native American women's psychological well-being and AIDS risk. *Journal of Consulting and Clinical Psychology,* *70*(1), 252-7. |
| [Hopwood, C. J., Ansell, E. B., Fehon, D. C., & Grilo, C. M. (2011). The mediational significance of negative/depressive affect in the relationship of childhood maltreatment and eating disorder features in adolescent psychiatric inpatients. *Eating and Weight Disorders, 16*(1), e9–e16. https://doi.org/10.1007/BF03327515](https://doi.org/10.1007/BF03327515) |
| [Huang, C. C., Chen, Y., & Cheung, S. (2021). Early childhood exposure to intimate partner violence and teen depression symptoms in the U.S. *Health & Social Care in the Community, 29*(5), e47-e55. https://doi.org/10.1111/hsc.13240](https://doi.org/10.1111/hsc.13240) |
| [Hund, A. R., & Espelage, D. L. (2005). Childhood sexual abuse, disordered eating, alexithymia, and general distress: A mediation model. *Journal of Counseling Psychology, 52*(4), 559–573. https://doi.org/10.1037/0022-0167.52.4.559 .](https://doi.org/10.1037/0022-0167.52.4.559) |
| [Hund, A. R., & Espelage, D. L. (2006). Childhood emotional abuse and disordered eating among undergraduate females: Mediating influence of alexithymia and distress. *Child Abuse & Neglect, 30*(4), 393–407. https://doi.org/10.1016/j.chiabu.2005.11.003 .](https://doi.org/10.1016/j.chiabu.2005.11.003) |
| [Inanici, S. Y., Inanici, M. A., & Yoldemir, A. T. (2017). The relationship between subjective experience of childhood abuse and neglect and depressive symptoms during pregnancy. *Journal of Forensic and Legal Medicine, 49*, 76–80. https://doi.org/10.1016/j.jflm.2017.05.016](https://doi.org/10.1016/j.jflm.2017.05.016) |
| Jager-Hyman, S. (2012). *Nonsuicidal self-injury in a college sample: Risk factors, pathways, and diagnostic correlates* [Doctoral dissertation, Temple University]. |
| [James, C. A., Schwartz, D. R., Roberts, K. E., Hart, T. A., Loutfy, M. R., Myers, T., & Calzavara, L. (2012). Childhood emotional abuse and psychological distress in gay and bisexual men. *Journal of Aggression, Maltreatment & Trauma, 21*(8), 851–869. https://doi.org/10.1080/10926771.2012.719590](https://doi.org/10.1080/10926771.2012.719590) |
| [Jessar, A. J., Hamilton, J. L., Flynn, M., Abramson, L. Y., & Alloy, L. B. (2017). Emotional clarity as a mechanism linking emotional neglect and depressive symptoms during early adolescence. *The Journal of Early Adolescence, 37*(3), 414–432. https://doi.org/10.1177/0272431615609157](https://doi.org/10.1177/0272431615609157) |
| [Jin, X., Doukas, A., Beiting, M., & Viksman, A. (2014). Factors contributing to intimate partner violence among men in Kerala, India. *Journal of Family Violence, 29*(6), 643–652. https://doi.org/10.1007/s10896-014-9623-4](https://doi.org/10.1007/s10896-014-9623-4) |
| Kang, C., Shi, J., Gong, Y., Wei, J., Zhang, M., Ding, H., Wang, K., Yu, Y., Wang, S., & Han, J. (2020). Interaction between FKBP5 polymorphisms and childhood trauma on depressive symptoms in Chinese adolescents: The moderating role of resilience. *Journal of Affective Disorders, 266*, 143–150. https://doi.org/10.1016/j.jad.2020.01.051. |
| [Kecojevic, A., Wong, C. F., Corliss, H. L., & Lankenau, S. E. (2015). Risk factors for high levels of prescription drug misuse and illicit drug use among substance-using young men who have sex with men (YMSM). *Drug and Alcohol Dependence, 150*, 156–163. https://doi.org/10.1016/j.drugalcdep.2015.02.031](https://doi.org/10.1016/j.drugalcdep.2015.02.031) |
| [Kilimnik, C. D., & Meston, C. M. (2016). Role of body esteem in the sexual excitation and inhibition responses of women with and without a history of childhood sexual abuse. *The Journal of Sexual Medicine, 13*(11), 1718–1728. https://doi.org/10.1016/j.jsxm.2016.09.004](https://doi.org/10.1016/j.jsxm.2016.09.004) |
| [Kim, J. S., Jin, M. J., Jung, W., Hahn, S. W., & Lee, S.-H. (2017). Rumination as a mediator between childhood trauma and adulthood depression/anxiety in nonclinical participants. *Frontiers in Psychology, 8*, 1–11. https://doi.org/10.3389/fpsyg.2017.01597](https://doi.org/10.3389/fpsyg.2017.01597) |
| [Klein, D. N., Arnow, B. A., Barkin, J. L., Dowling, F., Kocsis, J. H., Leon, A. C., Manber, R., Rothbaum, B. O., Trivedi, M. H., & Wisniewski, S. R. (2009). Early adversity in chronic depression: Clinical correlates and response to pharmacotherapy. *Depression and Anxiety, 26*(8), 701–710. https://doi.org/10.1002/da.20577](https://doi.org/10.1002/da.20577) |
| [Klein, H., Elifson, K. W., & Sterk, C. E. (2008). Depression and HIV risk behavior practices among At Risk women. *Women and Health, 48*(2), 167–188. https://doi.org/10.1080/03630240802313605](https://doi.org/10.1080/03630240802313605) |
| Kohrt, H. E., Kohrt, B. A., Waldman, I., Saltzman, K., & Carrion, V. G. (2004). An ecological–transactional model of significant risk factors for child psychopathology in outer Mongolia. *Child Psychiatry and Human Development, 35*(2), 163-181. |
| Kong, S., & Bernstein, K. (2009). Childhood trauma as a predictor of eating psychopathology and its mediating variables in patients with eating disorders. *Journal of Clinical Nursing,* *18*(13), 1897-907. |
| Kraaij, V., & de Wilde, E.J. (2001). Negative life events and depressive symptoms in the elderly: A life span perspective. *Aging & Mental Health,* *5*(1), 84-91. |
| [Krastins, A., Francis, A. J. P., Field, A. M., & Carr, S. N. (2014). Childhood predictors of adulthood antisocial personality disorder symptomatology. *Australian Psychologist, 49*(3), 142–150. https://doi.org/10.1111/ap.12048](https://doi.org/10.1111/ap.12048) |
| Kuo, J.R., Goldin, P.R., Werner, K., Heimberg, R.G., & Gross, J.J. (2011). Childhood trauma and current psychological functioning in adults with social anxiety disorder. *Journal of Anxiety Disorders,* *25*(4), 467-73. |
| [Lang, A. J., Stein, M. B., Kennedy, C. M., & Foy, D. W. (2004). Adult psychopathology and intimate partner violence among survivors of childhood maltreatment. *Journal of Interpersonal Violence, 19*(10), 1102–1118. https://doi.org/10.1177/0886260504269090](https://doi.org/10.1177/0886260504269090) |
| [Lang, A. J., Rodgers, C. S., & Lebeck, M. M. (2006). Associations between maternal childhood maltreatment and psychopathology and aggression during pregnancy and postpartum. *Child Abuse & Neglect, 30*(1), 17–25. https://doi.org/10.1016/j.chiabu.2005.07.006](https://doi.org/10.1016/j.chiabu.2005.07.006) |
| Lang, A. J., Gartstein, M. A., Rodgers, C. S., & Lebeck, M. M. (2010). The impact of maternal childhood abuse on parenting and infant temperament. *Journal of Child and Adolescent Psychiatric Nursing, 23*(2), 100–110 10.1111/j.1744-6171.2010.00229.x |
| [Leenarts, L. E. W. W., Hoeve, M., Van de Ven, P. M., Lodewijks, H. P. B. B., & Doreleijers, T. A. H. H. (2013). Childhood maltreatment and motivation for treatment in girls in compulsory residential care. *Children and Youth Services Review, 35*(7), 1041–1047. https://doi.org/10.1016/j.childyouth.2013.04.001](https://doi.org/10.1016/j.childyouth.2013.04.001) |
| [Lewis, C. S., Griffing, S., Chu, M., Jospitre, T., Sage, R. E., Madry, L., & Primm, B. J. (2006). Coping and violence exposure as predictors of psychological functioning in domestic violence survivors. *Violence Against Women, 12*(4), 340–354. https://doi.org/10.1177/1077801206287285](https://doi.org/10.1177/1077801206287285) |
| Li, S., Zhao, F., & Yu, G. (2020). Childhood emotional abuse and depression among adolescents: Roles of deviant peer affiliation and gender. *Journal of International Violence*. https://doi.org/10.1177/0886260520918586. |
| [Liu, R. T., Choi, J. Y., Boland, E. M., Mastin, B. M., & Alloy, L. B. (2013). Childhood abuse and stress generation: The mediational effect of depressogenic cognitive styles. *Psychiatry Research, 206*(2-3), 217–222. https://doi.org/10.1016/j.psychres.2012.12.001](https://doi.org/10.1016/j.psychres.2012.12.001) |
| Liu, R.T., Jager-Hyman, S., Wagner, C.A., Alloy, L.B., & Gibb, B.E. (2012). Number of childhood abuse perpetrators and the occurrence of depressive episodes in adulthood. *Child Abuse & Neglect, 3*6(4), 323-32. |
| Liu, J., Gong, J., Nie, G., He, Y., Xiao, B., Shen, Y., & Luo, X. (2017). The mediating effects of childhood neglect on the association between schizotypal and autistic personality traits and depression in a non-clinical sample. *BMC Psychiatry, 17*, Article 352. https://doi.org/10.1186/s12888–017–1510–0. |
| [Locke, T. F., Newcomb, M. D., Duclos, A., & Goodyear, R. K. (2007). Psychosocial predictors and correlates of dysphoria in adolescent and young adult Latinas. *Journal of Community Psychology, 35*(2), 135–149. https://doi.org/10.1002/jcop.20139](https://doi.org/10.1002/jcop.20139) |
| [Lopez, V., Kopak, A., Robillard, A., Gillmore, M. R., Holliday, R. C., & Braithwaite, R. L. (2011). Pathways to sexual risk taking among female adolescent detainees. *Journal of Youth and Adolescence, 40*(8), 945–957. https://doi.org/10.1007/s10964-010-9623-5.](https://doi.org/10.1007/s10964-010-9623-5) |
| Lutenbacher, M. (2000). Perceptions of health status and the relationship with abuse history and mental health in low-income single mothers. *Journal of Family Nursing,* *6*(4),320-40. |
| [Luterek, J. A., Harb, G. C., Heimberg, R. G., & Marx, B. P. (2004). Interpersonal rejection sensitivity in childhood sexual abuse survivors: mediator of depressive symptoms and anger suppression. *Journal of Interpersonal Violence, 19*(1), 90-107. https://doi.org/10.1177/0886260503259052](https://doi.org/10.1177/0886260503259052) |
| [MacDonald, K., Thomas, M. L., MacDonald, T. M., & Sciolla, A. F. (2014). A perfect childhood? Clinical correlates of minimization and denial on the Childhood Trauma Questionnaire. *Journal of Interpersonal Violence, 30*(6), https://doi.org/10.1177/08862605145397610886260514539761](https://doi.org/10.1177/08862605145397610886260514539761) |
| Marquee-Flentje, S. L. (2015). *Psychological adjustment of adult female survivors of CSA as a function of developmental level, self-directed disclosure and parental support* (Publication No. 10123642) [Doctoral dissertation]. ProQuest Dissertations & Theses Global. |
| Marse, K. (2002). *Depression and perception of maternal rejection in latency age, African American children of alcoholic mothers.* Seton Hall University Dissertations and Theses (ETDS). 362. |
| [Martsolf, D. S. (2004). Childhood maltreatment and mental and physical health in Haitian adults. *Journal of Nursing Scholarship, 36*(4), 293–299. https://doi.org/10.1111/j.1547-5069.2004.04054.x](https://doi.org/10.1111/j.1547-5069.2004.04054.x) |
| [Massing-Schaffer, M., Liu, R. T., Kraines, M. A., Choi, J. Y., & Alloy, L. B. (2015). Elucidating the relation between childhood emotional abuse and depressive symptoms in adulthood: The mediating role of maladaptive interpersonal processes. *Personality and Individual Differences, 74*, 106–111. https://doi.org/10.1016/j.paid.2014.09.045](https://doi.org/10.1016/j.paid.2014.09.045) |
| [Mazzeo, S. E., Mitchell, K. S., & Williams, L. J. (2008). Anxiety, alexithymia, and depression as mediators of the association between childhood abuse and eating disordered behavior in African American and European American women. *Psychology of Women Quarterly, 32*(3), 267–280. https://doi.org/10.1111/j.1471-6402.2008.00435.x](https://doi.org/10.1111/j.1471-6402.2008.00435.x) |
| [McGinn, L. K., Cukor, D., & Sanderson, W. C. (2005). The relationship between parenting style, cognitive style, and anxiety and depression: Does increased early adversity influence symptom severity through the mediating role of cognitive style? *Cognitive Therapy and Research, 29*(2), 219–242. https://doi.org/10.1007/s10608-005-3166-1](https://doi.org/10.1007/s10608-005-3166-1) |
| [Mehta, S., Rice, D., Chan, A., Shapiro, A. P., Sequeira, K., & Teasell, R. W. (2017). Impact of Abuse on Adjustment and Chronic Pain Disability: A Structural Equation Model. *The Clinical Journal of Pain, 33*(8), 687-693. https://doi.org/10.1097/AJP.0000000000000469](https://doi.org/10.1097/AJP.0000000000000469) |
| Melmed, L. R. (2012). *Cognitive style as a mediator between parental psychological maltreatment and depression in adolescent boys* [Doctoral dissertation, Columbia University]. |
| Meyerson, L. A., Long, P. J., Miranda Jr, R., & Marx, B. P. (2002). The influence of childhood sexual abuse, physical abuse, family environment, and gender on the psychological adjustment of adolescents. *Child Abuse & Neglect, 26*(4), 387-405. |
| [Michopoulos, V., Powers, A., Moore, C., Villarreal, S., Ressler, K. J., & Bradley, B. (2015). The mediating role of emotion dysregulation and depression on the relationship between childhood trauma exposure and emotional eating. *Appetite, 91*, 129–136. https://doi.org/10.1016/j.appet.2015.03.036](https://doi.org/10.1016/j.appet.2015.03.036) |
| [Mikaeili, N., Barahmand, U., & Abdi, R. (2013). The prevalence of different kinds of child abuse and the characteristics that differentiate abused from nonabused male adolescents. *Journal of Interpersonal Violence, 28*(5), 975–996. https://doi.org/10.1177/0886260512459377](https://doi.org/10.1177/0886260512459377) |
| [Miller, A. B., Adams, L. M., Esposito-Smythers, C., Thompson, R., & Proctor, L. J. (2014). Parents and friendships: a longitudinal examination of interpersonal mediators of the relationship between child maltreatment and suicidal ideation. *Psychiatry Research, 220*(3), 998-1006. https://doi.org/10.1016/j.psychres.2014.10.009](https://doi.org/10.1016/j.psychres.2014.10.009) |
| [Miller, A. B., Jenness, J. L., Oppenheimer, C. W., Gottleib, A. L. B., Young, J. F., & Hankin, B. L. (2017). Childhood emotional maltreatment as a robust predictor of suicidal ideation: A 3-year multi-wave, prospective investigation. *Journal of Abnormal Child Psychology, 45*(1), 105–116. https://doi.org/10.1007/s10802-0160150-z](https://doi.org/10.1007/s10802-0160150-z) |
| Min, A., Park, S.-C., Jang, E. Y., Park, Y. C., & Choi, J. (2015). Variables linking school bullying and suicidal ideation in middle school students in South Korea. *African Journal of Psychiatry, 18*(3). |
| [Minnich, A. M., Gordon, K. H., Kwan, M. Y., & Troop-Gordon, W. (2017). Examining the mediating role of alexithymia in the association between childhood neglect and disordered eating behaviors in men and women. *Psychology of Men & Masculinity, 18*(4), 414–421. https://doi.org/10.1037/men0000060](https://doi.org/10.1037/men0000060) |
| [Miron, L. R., & Orcutt, H. K. (2014). Pathways from childhood abuse to prospective revictimization: depression, sex to reduce negative affect, and forecasted sexual behavior. *Child Abuse & Neglect, 38*(11), 1848-1859. https://doi.org/10.1016/j.chiabu.2014.10.004](https://doi.org/10.1016/j.chiabu.2014.10.004) |
| [Miron, L. R., Seligowski, A. V., Boykin, D. M., & Orcutt, H. K. (2016). The Potential Indirect Effect of Childhood Abuse on Posttrauma Pathology Through Self-Compassion and Fear of Self-Compassion. *Mindfulness, 7*(3), 596-605. https://doi.org/10.1007/s12671-016-0493-0](https://doi.org/10.1007/s12671-016-0493-0) |
| [Mitchell, K. S., & Mazzeo, S. E. (2005). Mediators of the association between abuse and disordered eating in undergraduate men. *Eating Behaviors, 6*(4), 318–327. https://doi.org/10.1016/j.eatbeh.2005.03.004](https://doi.org/10.1016/j.eatbeh.2005.03.004) |
| Monteiro, S., Matos, A. P., & Oliveira, S. (2015). The moderating effect of gender: Traumatic experiences and depression in adolescence. *Procedia-Social Behav Sci., 165*, 251–259. doi: 10.1016/j.sbspro.2014.12.629 |
| Morokoff, P. J., Redding, C. A., Harlow, L. L., Cho, S., Rossi, J. S., Meier, K. S., . . . Brown-Peterside, P. (2009). Associations of Sexual Victimization, Depression, and Sexual Assertiveness with Unprotected Sex: A Test of the Multifaceted Model of HIV Risk Across Gender. *Journal of Applied Biobehavioral Research, 14*(1), 30-54. |
| Oliver, L.E., & Whiffen, V.E. (2003). Perceptions of parents and partners and men's depressive symptoms. *Journal of Social and Personal Relationships,* *20*(5), 621-35. |
| [O’Mahen, H. A., Karl, A., Moberly, N., & Fedock, G. (2015). The association between childhood maltreatment and emotion regulation: Two different mechanisms contributing to depression? *Journal of Affective Disorders, 174*, 287–295. https://doi.org/10.1016/j.jad.2014.11.028](https://doi.org/10.1016/j.jad.2014.11.028) |
| [Palosaari, E., Punamaki, R. L., Qouta, S., & Diab, M. (2013). Intergenerational effects of war trauma among Palestinian families mediated via psychological maltreatment. *Child Abuse & Neglect, 37*(11), 955-968. https://doi.org/10.1016/j.chiabu.2013.04.006](https://doi.org/10.1016/j.chiabu.2013.04.006) |
| [Park, A., & Kim, Y. (2018). The longitudinal influence of child maltreatment on child obesity in South Korea: The mediating effects of low self-esteem and depressive symptoms. *Children and Youth Services Review, 87*, 34-40. https://doi.org/10.1016/j.childyouth.2018.02.012](https://doi.org/10.1016/j.childyouth.2018.02.012) |
| [Pieritz, K., Rief, W., & Euteneuer, F. (2015). Childhood adversities and laboratory pain perception. *Neuropsychiatric Disease and Treatment, 11*, 2109–2116. https://doi.org/10.2147/NDT.S87703](https://doi.org/10.2147/NDT.S87703) |
| [Powers, A., Ressler, K. J., & Bradley, R. G. (2009). The protective role of friendship on the effects of childhood abuse and depression. *Depression and Anxiety, 26*(1), 46–53. https://doi.org/10.1002/da.20534](https://doi.org/10.1002/da.20534) |
| Quevedo, K. M. (2008). *Developmental risk factors and patterns of continuity leading to adult depression.* (Ph.D.). University of Minnesota, UMI. (3424810) |
| [Rabinovitch, S. M., Kerr, D. C., Leve, L. D., & Chamberlain, P. (2015). Suicidal Behavior Outcomes of Childhood Sexual Abuse: Longitudinal Study of Adjudicated Girls. *Suicide Life Threat Behav, 45*(4), 431-447. https://doi.org/10.1111/sltb.12141](https://doi.org/10.1111/sltb.12141) |
| [Raes, F., & Hermans, D. (2008). On the mediating role of subtypes of rumination in the relationship between childhood emotional abuse and depressed mood: Brooding versus reflection. *Depression and Anxiety, 25*(12), 1067–1070. https://doi.org/10.1002/da.20447](https://doi.org/10.1002/da.20447) |
| Randolph, M.E., & Reddy, D.M. (2006). Sexual functioning in women with chronic pelvic pain: The impact of depression, support, and abuse. *The Journal of Sex Research,* *43*(1), 38-45. |
| [Rezaei, M., & Ghazanfari, F. (2016). The role of childhood trauma, early maladaptive schemas, emotional schemas and experimental avoidance on depression: A structural equation modeling. *Psychiatry Research, 246*, 407–414. https://doi.org/10.1016/j.psychres.2016.10.037](https://doi.org/10.1016/j.psychres.2016.10.037) |
| [Rich, C. L., Gidycz, C. A., Warkentin, J. B., Loh, C., & Weiland, P. (2005). Child and adolescent abuse and subsequent victimization: a prospective study. *Child Abuse & Neglect, 29*(12), 1373-1394. https://doi.0rg/10.1016/j.chiabu.2005.07.003](https://doi.0rg/10.1016/j.chiabu.2005.07.003) |
| [Riggs, S. A., & Kaminski, P. (2010). Childhood emotional abuse, adult attachment, and depression as predictors of relational adjustment and psychological aggression. *Journal of Aggression, Maltreatment & Trauma, 19*(1), 75–104. https://doi.org/10.1080/10926770903475976](https://doi.org/10.1080/10926770903475976) |
| Roatta, V. G. (2000). *Mediators of childhood sexual abuse and adult risk for HIV among African American drug using women* (Publication No. 9985083) [Doctoral dissertation, Carlos Albizu University]. ProQuest Dissertations and Theses Global. |
| Roosa, M.W., Reinholtz, C., & Angelini, P.J. (1999). The relation of child sexual abuse and depression in young women: Comparisons across four ethnic groups. *Journal of Abnormal Child Psychology,* *27*(1), 65-76. |
| [Salah, B. L. (2015). *Does perceived social support influence the relationship between childhood maltreatment and depressive symptoms?* [Master's thesis, University of Leiden]. Retrieved from https://openaccess.leidenuniv.nl/bitstream/handle/1887/36541/Ben%20Salah,%20L.-s0512060-MA%20Thesis%20CP2015.pdf?sequence=1](https://openaccess.leidenuniv.nl/bitstream/handle/1887/36541/Ben%20Salah,%20L.-s0512060-MA%20Thesis%20CP2015.pdf?sequence=1) |
| [Schulz, A., Schmidt, C. O., Appel, K., Mahler, J., Spitzer, C., Wingenfeld, K., ... Grabe, H. J. (2014). Psychometric functioning, socio-demographic variability of childhood maltreatment in the general population and its effects of depression. *International Journal of Methods in Psychiatric Research, 23*(3), 387–400. https://doi.org/10.1002/mpr.1447](https://doi.org/10.1002/mpr.1447) |
| [Schumm, J. A., Stines, L. R., Hobfoll, S. E., & Jackson, A. P. (2005). The double-barreled burden of child abuse and current stressful circumstances on adult women: The kindling effect of early traumatic experience. *Journal of Traumatic Stress, 18*(5), 467–476. https://doi.org/10.1002/jts.20054](https://doi.org/10.1002/jts.20054) |
| Seok, J.H., Lee, K.U., Kim, W., Lee, S.H., Kang, E.H., Ham, B.J., et al. (2012). Impact of early-life stress and resilience on patients with major depressive disorder. *Yonsei Medical Journal,* *53*(6), 1093-8. |
| [Shahar, B., Doron, G., & Szepsenwol, O. (2015). Childhood maltreatment, shame-proneness and self-criticism in social anxiety disorder: A sequential mediational model. *Clinical Psychology & Psychotherapy, 22*(6), 570–579. https://doi.org/10.1002/cpp.1918](https://doi.org/10.1002/cpp.1918) |
| Shao, N., Gong, Y., Wang, X., Wei, J., Shi, J., Ding, H., Zhang, M., Kang, C., Wang, S., Chen, L., Yu, Y., & Han, J. (2020). Effects of polygenic risk score, childhood trauma and resilience on depressive symptoms in Chinese adolescents in a three-year cohort study. *Journal of Affective Disorders*, *282*, 627–636. https://doi.org/10.1016/j.jad.2020.12.114. |
| Shapero, B. G., Hamilton, J. L., Liu, R. T., Abramson, L. Y., & Alloy, L. B. (2013). Internalizing symptoms and rumination: The prospective prediction of familial and peer emotional victimization experiences during adolescence. *Journal of Adolescence, 36*(6), 1067–1076 |
| [Shi, L. (2013). Childhood abuse and neglect in an outpatient clinical sample: Prevalence and Impact. *The American Journal of Family Therapy, 41*(3), 198–211. https://doi.org/10.1080/01926187.2012.677662](https://doi.org/10.1080/01926187.2012.677662) |
| [Song, J.-M., Min, J.-A., Huh, H.-J., & Chae, J.-H. (2016). Types of childhood trauma and spirituality in adult patients with depressive disorders. *Comprehensive Psychiatry, 69*, 11–19. https://doi.org/10.1016/j.comppsych.2016.04.003](https://doi.org/10.1016/j.comppsych.2016.04.003) |
| Song, X., Wang, S., Wang, R., Xu, H., Jiang, Z., Li, S., Zhang, S., & Wan, Y. (2020). Mediating effects of specific types of coping styles on the relationship between childhood maltreatment and depressive symptoms among Chinese undergraduates: The role of sex. *International Journal of Environmental Research and Public Health, 17*(9), Article 3120. https://doi.org/10.3390/ijerph17093120. |
| [Spertus, I. L., Yehuda, R., Wong, C. M., Halligan, S., & Seremetis, S. V. (2003). Childhood emotional abuse and neglect as predictors of psychological and physical symptoms in women presenting to a primary care practice. *Child Abuse & Neglect, 27*(11), 1247–1258. https://doi.org/10.1016/j.chiabu.2003.05.001](https://doi.org/10.1016/j.chiabu.2003.05.001) |
| [Spinhoven, P., Penninx, B. B. W., Hickendorff, M., van Hemert, A. M., Bernstein, D. P., & Elzinga, B. M. (2014). Childhood Trauma Questionnaire: Factor structure, measurement invariance, and validity across emotional disorders. *Psychological Assessment, 26*(3), 717. https://doi.org/10.1037/pas0000002](https://doi.org/10.1037/pas0000002) |
| [Stange, J. P., Hamilton, J. L., Abramson, L. Y., & Alloy, L. B. (2014). A vulnerability-stress examination of response styles theory in adolescence: Stressors, sex differences, and symptom specificity. *Journal of Clinical Child & Adolescent Psychology, 43*(5), 813–827. https://doi.org/10.1080/15374416.2013.812037](https://doi.org/10.1080/15374416.2013.812037) |
| Steffey, S. K. (2012). *Childhood Issues & Adulthood Tissues: Examining Relationships between Childhood Abuse and Adult Depression.* The Honors Conference Anthology of the Northeast State Community College Honors Program, presented April 20 (p. 42). |
| [Stewart, J. G., Kim, J. C., Esposito, E. C., Gold, J., Nock, M. K., & Auerbach, R. P. (2015). Predicting suicide attempts in depressed adolescents: Clarifying the role of disinhibition and childhood sexual abuse. *Journal of Affective Disorders, 187*, 27–34. https://doi.org/10.1016/j.jad.2015.08.034](https://doi.org/10.1016/j.jad.2015.08.034) |
| [Tanaka, M., Wekerle, C., Schmuck, M. L., Paglia-Boak, A., & The MAP Research Team (2011). The linkages among childhood maltreatment, adolescent mental health, and self-compassion in child welfare adolescents. *Child Abuse & Neglect, 35*(10), 887–898. https://doi.org/10.1016/j.chiabu.2011.07.003](https://doi.org/10.1016/j.chiabu.2011.07.003) |
| Tang, W., Zhao, J., Lu, Y., Zha, Y., Liu, H., Sun, Y., Zhang, J., Yang, Y., & Xu, J. (2018). Suicidality, posttraumatic stress, and depressive reactions after earthquake and maltreatment: A cross-sectional survey of a random sample of 6132 Chinese children and adolescents. *Journal of Affective Disorders, 232*, 363–369. https://doi.org/10.1016/j.jad.2018.02.081. |
| [Thomas, R., DiLillo, D., Walsh, K., & Polusny, M. A. (2011). Pathways from child sexual abuse to adult depression: The role of parental socialization of emotions and alexithymia. *Psychology of Violence, 1*(2), 121-135. https://doi.org/10.1037/a0022469](https://doi.org/10.1037/a0022469) |
| [Tlapek, S. M., Auslander, W., Edmond, T., Gerke, D., Schrag, R. V., & Threlfall, J. (2017). The moderating role of resiliency on the negative effects of childhood abuse for adolescent girls involved in child welfare. *Children and Youth Services Review, 73*, 437–444. https://doi.org/10.1016/j.childyouth.2016.11.026](https://doi.org/10.1016/j.childyouth.2016.11.026) |
| [Turner, H. A., & Muller, P. A. (2004). Long-Term Effects of Child Corporal Punishment on Depressive Symptoms in Young Adults. *Journal of Family Issues, 25*(6), 761-782. https://doi.org/10.1177/0192513x03258313](https://doi.org/10.1177/0192513x03258313) |
| [Ullman, S. E., Peter-Hagene, L. C., & Relyea, M. (2014). Coping, emotion regulation, and selfblame as mediators of sexual abuse and psychological symptoms in adult sexual assault. *Journal of Child Sexual Abuse, 23*(1), 74-93. https://doi.org/10.1080/10538712.2014.864747](https://doi.org/10.1080/10538712.2014.864747) |
| [Van Vugt, E., Lanctôt, N., Paquette, G., Collin-Vézina, D., & Lemieux, A. (2014). Girls in residential care: From child maltreatment to trauma-related symptoms in emerging adulthood. *Child Abuse & Neglect, 38*(1), 114–122. https://doi.org/10.1016/j.chiabu.2013.10.015](https://doi.org/10.1016/j.chiabu.2013.10.015) |
| Virkler, P. M. (2005). *The relationship between childhood sexual abuse and measures of depression, anxiety and revictimization in females aged 55 to 85*. [Doctoral dissertation, University of North Carolina – Charlotte]. |
| [Voth Schrag, R. J., Edmond, T., Tlapek, S. M., & Auslander, W. (2017). Exposure to economically abusive tactics among adolescent girls in the child welfare system. *Child and Adolescent Social Work Journal, 34*(2), 127–136. https://doi.org/10.1007/s10560-016-0450-8](https://doi.org/10.1007/s10560-016-0450-8) |
| Wang, J. & Liu, A. (2015). Mediating role of regulatory emotional self-efficacy between childhood abuse and depression in college students. *Chinese Mental Health Journal, 29*, 305-310. https://doi.org/10.3969/j.issn.1000-6729.2015.04.013 |
| [Wanklyn, S. G., Day, D. M., Hart, T. A., & Girard, T. A. (2012). Cumulative childhood maltreatment and depression among incarcerated youth: impulsivity and hopelessness as potential intervening variables. *Child Maltreatment, 17*(4), 306–317. https://doi.org/10.1177/1077559512466956](https://doi.org/10.1177/1077559512466956) |
| Weissmann, Wind, T., & Silvern, L.E. (1992). Type and extent of child abuse as predictors of adult functioning. *Journal of Family Violence,* *7*(4), 261-281. |
| Whiffen, V.E., Judd, M.E., & Aube, J.A. (1999). Intimate relationships moderate the association between childhood sexual abuse and depression. *Journal of Interpersonal Violence,* *14*(9), 940-54. |
| Whiffen, V. E., Thompson, J. M., & Aube, J. A. (2000). Mediators of the link between childhood sexual abuse and adult depressive symptoms. *Journal of interpersonal violence, 15*(10), 1100-1120. |
| [Woods, S. J., Kozachik, S. L., & Hall, R. J. (2010). Subjective sleep quality in women experiencing intimate partner violence: Contributions of situational, psychological, and physiological factors. *Journal of Traumatic Stress, 23*(1), 141–150. https://doi.org/10.1002/jts.20495](https://doi.org/10.1002/jts.20495) |
| [Wu, Q., Chi, P., Lin, X., & Du, H. (2018). Child maltreatment and adult depressive symptoms: Roles of self-compassion and gratitude. *Child Abuse & Neglect, 80*, 62–69. https://doi.org/10.1016/j.chiabu.2018.03.013](https://doi.org/10.1016/j.chiabu.2018.03.013) |
| [Wuest, J., Ford-Gilboe, M., Merritt-Gray, M., Wilk, P., Campbell, J. C., Lent, B., ... Smye, V. (2010). Pathways of chronic pain in survivors of intimate partner violence. *Journal of Women’s Health, 19*(9), 1665–1674. https://doi.org/10.1089/jwh.2009.1856](https://doi.org/10.1089/jwh.2009.1856) |
| [Zalewski, M., Cyranowski, J. M., Cheng, Y., & Swartz, H. A. (2013). Role of maternal childhood trauma on parenting among depressed mothers of psychiatrically ill children. *Depression and Anxiety, 30*(9), 792–799. https://doi.org/10.1002/da.22116.](https://doi.org/10.1002/da.22116) |
| Zhang, G., Liu D., Li P., Long, Y., Chen, X., Mao, K., Zhu, H., Ma, C., Wang, Q., Gao, Y., Lin, J., & Tan, H. (2020). Association between childhood abuse and postpartum depression. (2020). *Shandong Archives of Psychiatry*, *33*(1). https://doi.org/10.3969/j.issn.2095–9346.2020.01.006. |
| Zhang, Y. H., Li, J. Y., Yin, X. Q., & Wang, J. L. (2022). Relative weights analysis on the impacts of childhood maltreatment forms on adolescent anxiety and depression. *Chin J Sch Health., 43*, 407–410. doi: 10.16835/j.cnki.1000-9817.2022.03.021 |
| Zhou, J., Feng, L., Hu, C., Pao, C., Xiao, L., & Wang, G. (2019). Associations among depressive symptoms, childhood abuse, neuroticism, social support, and coping style in the population covering general adults, depressed patients, bipolar disorder patients, and high-risk population for depression. *Frontiers in Psychology, 10*, Article 1321. https://doi.org/10.3389/fpsyg.2019.01321. |

| **References (meta-analyses used for data extraction, from Table 1)** |
| --- |
| Gardner, M. J., Thomas, H. J., & Erskine, H. E. (2019). The association between five forms of child maltreatment and depressive and anxiety disorders: A systematic review and meta-analysis. *Child Abuse & Neglect, 96*, 104082. |
| Humphreys, K. L., LeMoult, J., Wear, J. G., Piersiak, H. A., Lee, A., & Gotlib, I. H. (2020). Child maltreatment and depression: A meta-analysis of studies using the Childhood Trauma Questionnaire. *Child Abuse & Neglect, 102*, 104361. |
| [Infurna, M. R., Reichl, C., Parzer, P., Schimmenti, A., Bifulco, A., & Kaess, M. (2016). Associations between depression and specific childhood experiences of abuse and neglect: A meta-analysis. *Journal of Affective Disorders, 190*, 47–55. https://doi.org/10.1016/j.jad.2015.09.006](https://doi.org/10.1016/j.jad.2015.09.006) |
| Lai, C.-L. J., Fan, Y., Man, H. Y., & Huang, Y. (2023). Childhood adversity and depression in Chinese populations: A multilevel meta-analysis of studies using the Childhood Trauma Questionnaire (CTQ). *Asian Journal of Psychiatry, 84*, 103582, doi.org/10.1016/j.ajp.2023.103582. |
| LeMoult, J., Humphreys, K. L., Tracy, A., Hoffmeister, J.-A., Ip, E., & Gotlib, I. H. (2020). Meta-analysis: Exposure to early life stress and risk for depression in childhood and adolescence. *Journal of the American Academy of Child & Adolescent Psychiatry, 59*(7), 842–855. |
| Li, M., Gao, T., & Meng, X. (2023). The Timing effect of childhood maltreatment in depression: A Systematic review and meta-analysis. *Trauma, Violence, & Abuse, 24(4)*, 2560-2580. |
| [Mandelli, L., Petrelli, C., & Serretti, A. (2015). The role of specific early trauma in adult depression: A meta-analysis of published literature. Childhood trauma and adult depression. *European Psychiatry, 30*(6), 665–680. https://doi.org/10.1016/j.eurpsy.2015.04.007](https://doi.org/10.1016/j.eurpsy.2015.04.007) |
| [Nelson, J., Klumparendt, A., Doebler, P., & Ehring, T. (2017). Childhood maltreatment and characteristics of adult depression: Meta-analysis. *The British Journal of Psychiatry, 210*(2), 96–104. https://doi.org/10.1192/bjp.bp.115.180752.](https://doi.org/10.1192/bjp.bp.115.180752) |
| Tan, M., & Mao, P. (2023). Type and dose-response effect of adverse childhood experiences in predicting depression: A Systematic review and meta-analysis. *Child Abuse & Neglect, 139*, 106091. |
| Wang, Y., Chen, X., Zhou, K., & Zhang, H. (2023). A Meta-analysis of the effects of childhood maltreatment on elderly depression. *Trauma, Violence, & Abuse, 24*(3), 1593-1607 |
| Yu, Z., Zhao, A., & Liu, A. (2017). Childhood maltreatment and depression: A Meta-analysis. *Acta Psychologica Sinica, 49*(1), 40-49. |
| Yu Z, Cao Y, Shang T, & Li P. (2024). Depression in youths with early life adversity: A systematic review and meta-analysis. *Frontiers in Psychiatry,* *15*:1378807. https://doi:10.3389/fpsyt.2024.1378807 |
|  |
| **References (other meta-analyses in Table 1)** |
| Kuzminskaite, E. et al. (2022). Treatment efficacy and effectiveness in adults with major depressive disorder and childhood trauma history: A systematic review and meta-analysis. *The Lancet Psychiatry, 9*, 860 – 873. |
| Souama, C., Lamers, F., Milaneschi, Y., Vinkers, C., Defina, S. et al. (2023). Depression, cardiometabolic disease, and their co-occurrence after childhood maltreatment: An individual participant data meta-analysis including over 200,000 participants. *BMC Medicine, 21*:93. |

**Code for data from source studies (data provided afterwards)**

| **label** | **meaning** |
| --- | --- |
| ln OR | log of odds ratio |
| se(ln OR) | standard error of log of odds ratio |
| ln zr | log of Fisher z |
| se(ln zr) | standard error of log of Fisher z |
| CM | child maltreatment subtype |
| EA | emotional abuse |
| EN | emotional neglect |
| PA | physical abuse |
| PN | physical neglect |
| SA | sexual abuse |
| Complete abuse |  |
| 1 | study assessed all 3 abuses (EA, PA, SA) |
| 0 | study assessed only 1 or 2 of the abuses |
| CM measure (for OR) | |
| 1 | ACE/ACE-IQ |
| 2 | CECA |
| 3 | CTQ |
| 4 | CTS |
| 5 | self-report |
| 6 | other |
| CM measure (for zr) |  |
| 0 | other |
| 1 | CTQ |
| Sample type |  |
| 1 | case-control |
| 2 | clinical |
| 3 | convenience |
| 4 | representative |
| Country code |  |
| 1 | English-speaking country (Australia, Canada, New Zealand, UK, USA) |
| 2 | European |
| 3 | Asian |
| 4 | New World |
| 5 | Middle East, Africa |
| Effective N | actual number of participants used in given analysis |
| % female | percentage of participants in sample who were female |
| English speaking |  |
| 1 | participants spoke English; instrument in English |
| 0 | not above |

**Risk data**

| **study** | **year** | **ln OR** | **se(ln OR)** | **CM** | **complete abuse** | **CM meas** | **sample** | **country** | **effective N** | **% fem** | **Eng speak** |
| --- | --- | --- | --- | --- | --- | --- | --- | --- | --- | --- | --- |
| Afifi 2006(1) | 2006 | 0.94 | 0.17 | PA | 0 | 5 | 4 | 5 | 2077 | 0.51 | 0 |
| Afifi 2006(2) | 2006 | 0.36 | 0.09 | PA | 0 | 5 | 4 | 5 | 5409 | 0.49 | 0 |
| Afifi 2006(3) | 2006 | 0.20 | 0.10 | PA | 0 | 4 | 4 | 1 | 5838 | 0.51 | 1 |
| Afifi 2012 | 2012 | 0.34 | 0.16 | PA | 0 | 1 | 4 | 1 | 20607 | . | 1 |
| Agbaje 2021 | 2021 | 0.60 | 0.31 | EA | 1 | 1 | 3 | 5 | 203 | 0.59 | 1 |
| Agbaje 2021 | 2021 | -0.39 | 0.62 | EN | 1 | 1 | 3 | 5 | 203 | 0.59 | 1 |
| Agbaje 2021 | 2021 | 0.36 | 0.33 | PA | 1 | 1 | 3 | 5 | 203 | 0.59 | 1 |
| Agbaje 2021 | 2021 | 1.05 | 0.31 | PN | 1 | 1 | 3 | 5 | 203 | 0.59 | 1 |
| Agbaje 2021 | 2021 | 1.45 | 0.30 | SA | 1 | 1 | 3 | 5 | 203 | 0.59 | 1 |
| Ahmadkhaniha 2007 | 2007 | 1.16 | 0.58 | SA | 0 | 6 | 3 | 5 | 87 | 0.36 | 0 |
| Allen 1989 | 1989 | 1.47 | 0.72 | PA | 0 | 6 | 1 | 1 | 36 | 0.28 | 1 |
| Allers 1991 | 1991 | 0.69 | 0.76 | PA | 0 | 6 | 3 | 1 | 52 | 0.13 | 1 |
| Allers 1991 | 1991 | 1.92 | 0.71 | SA | 0 | 6 | 3 | 1 | 52 | 0.13 | 1 |
| Almeida 2011 | 2011 | 1.15 | 0.10 | PA | 0 | 5 | 3 | 1 | 20677 | 0.58 | 1 |
| Almeida 2011 | 2011 | 0.91 | 0.10 | SA | 0 | 5 | 3 | 1 | 20677 | 0.58 | 1 |
| Anda 2002 | 2002 | 1.21 | 0.07 | EA | 1 | 4 | 3 | 1 | 9346 | 0.54 | 1 |
| Anda 2002 | 2002 | 0.72 | 0.05 | PA | 1 | 4 | 3 | 1 | 9346 | 0.54 | 1 |
| Anda 2002 | 2002 | 0.69 | 0.06 | SA | 1 | 4 | 3 | 1 | 9346 | 0.54 | 1 |
| Angst 2011 | 2011 | 0.96 | 0.40 | SA | 0 | 5 | 4 | 2 | 591 | 0.51 | 0 |
| Appel 2011 | 2011 | 1.55 | 0.24 | EA | 1 | 3 | 4 | 2 | 2157 | 0.53 | 0 |
| Appel 2011 | 2011 | 0.76 | 0.15 | EN | 1 | 3 | 4 | 2 | 2157 | 0.53 | 0 |
| Appel 2011 | 2011 | 0.66 | 0.23 | PA | 1 | 3 | 4 | 2 | 2157 | 0.53 | 0 |
| Appel 2011 | 2011 | 0.32 | 0.15 | PN | 1 | 3 | 4 | 2 | 2157 | 0.53 | 0 |
| Appel 2011 | 2011 | 0.92 | 0.25 | SA | 1 | 3 | 4 | 2 | 2157 | 0.53 | 0 |
| Armour 2016 | 2016 | 0.97 | 0.31 | PA | 0 | 6 | 4 | 1 | 3845 | 1.00 | 1 |
| As-sanie 2014 | 2014 | 0.63 | 0.24 | PA | 0 | 6 | 2 | 1 | 273 | 1.00 | 1 |
| As-sanie 2014 | 2014 | 0.83 | 0.25 | SA | 0 | 6 | 2 | 1 | 273 | 1.00 | 1 |
| Avanci 2008 | 2008 | 1.97 | 0.31 | EA | 1 | 6 | 4 | 4 | 1923 | 0.56 | 0 |
| Avanci 2008 | 2008 | 0.53 | 0.26 | SA | 1 | 6 | 4 | 4 | 1923 | 0.56 | 0 |
| Avanci 2008 | 2008 | 0.69 | 0.21 | PA | 1 | 6 | 4 | 4 | 1923 | 0.56 | 0 |
| Avanci 2012 | 2012 | 1.56 | 0.44 | EA | 0 | 6 | 4 | 4 | 464 | 0.48 | 0 |
| Avanci 2012 | 2012 | 1.16 | 0.55 | PA | 0 | 6 | 4 | 4 | 464 | 0.48 | 0 |
| Bandelow 2013 | 2013 | 0.92 | 0.48 | PA | 0 | 5 | 1 | 2 | 189 | 0.52 | 0 |
| Bandelow 2013 | 2013 | -0.39 | 0.63 | SA | 0 | 5 | 1 | 2 | 189 | 0.52 | 0 |
| Bauriedl-Schmidt 2017 | 2017 | 2.21 | 0.45 | EA | 1 | 3 | 1 | 2 | 81 | 0.48 | 0 |
| Bauriedl-Schmidt 2017 | 2017 | 1.82 | 0.44 | EN | 1 | 3 | 1 | 2 | 81 | 0.48 | 0 |
| Bauriedl-Schmidt 2017 | 2017 | 1.15 | 0.43 | PA | 1 | 3 | 1 | 2 | 81 | 0.48 | 0 |
| Bauriedl-Schmidt 2017 | 2017 | 1.20 | 0.43 | PN | 1 | 3 | 1 | 2 | 81 | 0.48 | 0 |
| Bauriedl-Schmidt 2017 | 2017 | 0.99 | 0.43 | SA | 1 | 3 | 1 | 2 | 81 | 0.48 | 0 |
| Benedict 1999 | 1999 | 2.23 | 0.47 | SA | 0 | 6 | 3 | 1 | 357 | 1.00 | 1 |
| Bermingham 2012 | 2012 | 1.69 | 0.41 | EA | 1 | 3 | 1 | 2 | 88 | 0.63 | 1 |
| Bermingham 2012 | 2012 | 1.96 | 0.41 | EN | 1 | 3 | 1 | 2 | 88 | 0.63 | 1 |
| Bermingham 2012 | 2012 | 1.25 | 0.40 | PA | 1 | 3 | 1 | 2 | 88 | 0.63 | 1 |
| Bermingham 2012 | 2012 | 1.45 | 0.40 | PN | 1 | 3 | 1 | 2 | 88 | 0.63 | 1 |
| Bermingham 2012 | 2012 | 1.15 | 0.40 | SA | 1 | 3 | 1 | 2 | 88 | 0.63 | 1 |
| Bernet 1999 | 1999 | 1.57 | 0.41 | EA | 1 | 3 | 1 | 1 | 88 | 0.50 | 1 |
| Bernet 1999 | 1999 | 1.07 | 0.40 | EN | 1 | 3 | 1 | 1 | 88 | 0.50 | 1 |
| Bernet 1999 | 1999 | 1.20 | 0.40 | PA | 1 | 3 | 1 | 1 | 88 | 0.50 | 1 |
| Bernet 1999 | 1999 | 0.88 | 0.39 | PN | 1 | 3 | 1 | 1 | 88 | 0.50 | 1 |
| Bernet 1999 | 1999 | 0.75 | 0.39 | SA | 1 | 3 | 1 | 1 | 88 | 0.50 | 1 |
| Bifulco 1991 | 1991 | 1.64 | 0.44 | SA | 0 | 5 | 3 | 1 | 286 | 1.00 | 1 |
| Bifulco 2002 | 2002 | 1.73 | 0.53 | EA | 1 | 2 | 3 | 1 | 277 | 0.50 | 1 |
| Bifulco 2002 | 2002 | 1.02 | 0.30 | PA | 1 | 2 | 3 | 1 | 277 | 0.50 | 1 |
| Bifulco 2002 | 2002 | 0.69 | 0.30 | SA | 1 | 2 | 3 | 1 | 277 | 0.50 | 1 |
| Blanchard 2014 | 2014 | 0.46 | 0.53 | PA | 0 | 6 | 2 | 1 | 71 | 0.80 | 1 |
| Blanchard 2014 | 2014 | 0.79 | 0.58 | SA | 0 | 6 | 2 | 1 | 71 | 0.80 | 1 |
| Boecking 2014 | 2014 | 0.79 | 0.58 | EA | 1 | 3 | 1 | 1 | 40 | 0.60 | 1 |
| Boecking 2014 | 2014 | 1.33 | 0.59 | EN | 1 | 3 | 1 | 1 | 40 | 0.60 | 1 |
| Boecking 2014 | 2014 | 0.55 | 0.58 | PA | 1 | 3 | 1 | 1 | 40 | 0.60 | 1 |
| Boecking 2014 | 2014 | 0.90 | 0.58 | PN | 1 | 3 | 1 | 1 | 40 | 0.60 | 1 |
| Boecking 2014 | 2014 | 0.03 | 0.57 | SA | 1 | 3 | 1 | 1 | 40 | 0.60 | 1 |
| Bonomi 2008 | 2008 | 0.83 | 0.11 | PA | 0 | 6 | 3 | 1 | 3568 | 1.00 | 1 |
| Bonomi 2008 | 2008 | 0.46 | 0.09 | SA | 0 | 6 | 3 | 1 | 3568 | 1.00 | 1 |
| Brezo 2010 | 2010 | 0.77 | 0.21 | PA | 0 | 1 | 4 | 1 | 1121 | . | 0 |
| Brezo 2010 | 2010 | 1.13 | 0.21 | SA | 0 | 1 | 4 | 1 | 1121 | . | 0 |
| Briere 1988 | 1988 | 0.70 | 0.35 | SA | 0 | 6 | 2 | 1 | 93 | 1.00 | 1 |
| Briere 1997 | 1997 | 0.59 | 0.45 | PA | 0 | 6 | 3 | 1 | 278 | 1.00 | 1 |
| Briere 1997 | 1997 | 1.38 | 0.48 | SA | 0 | 6 | 3 | 1 | 278 | 1.00 | 1 |
| Brock 1997 | 1997 | 1.18 | 0.46 | SA | 0 | 6 | 3 | 1 | 80 | 1.00 | 1 |
| Brown 1999 | 1999 | 0.86 | 0.50 | PA | 0 | 5 | 3 | 1 | 639 | 0.48 | 1 |
| Brown 1999 | 1999 | 1.15 | 0.57 | SA | 0 | 5 | 3 | 1 | 639 | 0.48 | 1 |
| Bulik 1989 | 1989 | 3.22 | 1.14 | SA | 0 | 6 | 2 | 1 | 35 | 1.00 | 1 |
| Carbelledo 2013 | 2013 | 1.15 | 0.32 | EA | 1 | 3 | 1 | 2 | 133 | 0.62 | 1 |
| Carbelledo 2013 | 2013 | 1.40 | 0.33 | EN | 1 | 3 | 1 | 2 | 133 | 0.62 | 1 |
| Carbelledo 2013 | 2013 | 1.02 | 0.32 | PA | 1 | 3 | 1 | 2 | 133 | 0.62 | 1 |
| Carbelledo 2013 | 2013 | 1.39 | 0.33 | PN | 1 | 3 | 1 | 2 | 133 | 0.62 | 1 |
| Carbelledo 2013 | 2013 | 0.72 | 0.32 | SA | 1 | 3 | 1 | 2 | 133 | 0.62 | 1 |
| Carey 2008 | 2008 | -0.11 | 0.41 | SA | 0 | 3 | 2 | 2 | 94 | 0.63 | 1 |
| Chaney 2014 | 2014 | 2.11 | 0.43 | EA | 1 | 3 | 1 | 2 | 83 | 0.59 | 1 |
| Chaney 2014 | 2014 | 2.40 | 0.44 | EN | 1 | 3 | 1 | 2 | 83 | 0.59 | 1 |
| Chaney 2014 | 2014 | 1.42 | 0.42 | PA | 1 | 3 | 1 | 2 | 83 | 0.59 | 1 |
| Chaney 2014 | 2014 | 1.79 | 0.42 | PN | 1 | 3 | 1 | 2 | 83 | 0.59 | 1 |
| Chaney 2014 | 2014 | 1.62 | 0.42 | SA | 1 | 3 | 1 | 2 | 83 | 0.59 | 1 |
| Chapman 2004f | 2004 | 0.99 | 0.08 | EA | 1 | 4 | 3 | 1 | 5108 | 1.00 | 1 |
| Chapman 2004f | 2004 | 0.83 | 0.08 | PA | 1 | 4 | 3 | 1 | 5108 | 1.00 | 1 |
| Chapman 2004f | 2004 | 0.69 | 0.08 | SA | 1 | 4 | 3 | 1 | 5108 | 1.00 | 1 |
| Chapman 2004m | 2004 | 0.92 | 0.13 | EA | 1 | 4 | 3 | 1 | 4352 | 0.00 | 1 |
| Chapman 2004m | 2004 | 0.47 | 0.14 | PA | 1 | 4 | 3 | 1 | 4352 | 0.00 | 1 |
| Chapman 2004m | 2004 | 0.47 | 0.14 | SA | 1 | 4 | 3 | 1 | 4352 | 0.00 | 1 |
| Cheasty 1998 | 1998 | 0.69 | 0.29 | SA | 0 | 5 | 3 | 2 | 237 | 1.00 | 1 |
| Chou 2012 | 2012 | 0.63 | 0.42 | SA | 0 | 5 | 4 | 1 | 3493 | . | 1 |
| Chung 2008 | 2008 | 0.47 | 0.14 | EA | 1 | 5 | 3 | 1 | 1476 | 1.00 | 1 |
| Chung 2008 | 2008 | 0.57 | 0.13 | PA | 1 | 5 | 3 | 1 | 1476 | 1.00 | 1 |
| Chung 2008 | 2008 | 0.89 | 0.17 | SA | 1 | 5 | 3 | 1 | 1476 | 1.00 | 1 |
| Cisler 2013 | 2013 | 3.23 | 0.69 | EA | 1 | 3 | 3 | 1 | 38 | 1.00 | 1 |
| Cisler 2013 | 2013 | 3.49 | 0.71 | EN | 1 | 3 | 3 | 1 | 38 | 1.00 | 1 |
| Cisler 2013 | 2013 | 2.15 | 0.64 | PA | 1 | 3 | 3 | 1 | 38 | 1.00 | 1 |
| Cisler 2013 | 2013 | 2.53 | 0.66 | PN | 1 | 3 | 3 | 1 | 38 | 1.00 | 1 |
| Cisler 2013 | 2013 | 2.68 | 0.66 | SA | 1 | 3 | 3 | 1 | 38 | 1.00 | 1 |
| Cohen 1996 | 1996 | 0.34 | 0.46 | PA | 0 | 6 | 2 | 1 | 105 | 0.70 | 1 |
| Cohen 1996 | 1996 | -0.33 | 0.46 | SA | 0 | 6 | 2 | 1 | 105 | 0.70 | 1 |
| Cohen 2001 | 2001 | 0.68 | 0.65 | PA | 0 | 5 | . | 1 | 610 | . | 1 |
| Cohen 2001 | 2001 | 1.57 | 0.56 | SA | 0 | 5 | . | 1 | 610 | . | 1 |
| Coles 2015 | 2015 | 0.36 | 0.09 | SA | 0 | 5 | 4 | 1 | 7700 | 1.00 | 1 |
| Comijs 2013 | 2013 | 1.71 | 0.38 | EA | 1 | 6 | 1 | 2 | 510 | 0.65 | 0 |
| Comijs 2013 | 2013 | 2.27 | 0.36 | EN | 1 | 6 | 1 | 2 | 510 | 0.65 | 0 |
| Comijs 2013 | 2013 | 2.51 | 0.73 | PA | 1 | 6 | 1 | 2 | 510 | 0.65 | 0 |
| Comijs 2013 | 2013 | 1.65 | 0.41 | SA | 1 | 6 | 1 | 2 | 510 | 0.65 | 0 |
| Cong 2012 | 2012 | 1.37 | 0.14 | SA | 0 | 6 | 1 | 3 | 4527 | 1.00 | 0 |
| Cyranowski 2012 | 2012 | 1.52 | 0.31 | EA | 1 | 3 | 4 | 1 | 443 | 1.00 | 1 |
| Cyranowski 2012 | 2012 | 1.40 | 0.47 | EN | 1 | 3 | 4 | 1 | 443 | 1.00 | 1 |
| Cyranowski 2012 | 2012 | 1.27 | 0.34 | PA | 1 | 3 | 4 | 1 | 443 | 1.00 | 1 |
| Cyranowski 2012 | 2012 | 0.48 | 0.32 | PN | 1 | 3 | 4 | 1 | 443 | 1.00 | 1 |
| Cyranowski 2012 | 2012 | 1.42 | 0.35 | SA | 1 | 3 | 4 | 1 | 443 | 1.00 | 1 |
| Danielson 2005 | 2005 | 0.45 | 0.18 | PA | 0 | 6 | 3 | 1 | 548 | 0.64 | 1 |
| Dannehl 2017 | 2017 | 0.83 | 0.35 | EA | 1 | 3 | 1 | 2 | 131 | 0.64 | 0 |
| Dannehl 2017 | 2017 | 2.31 | 0.37 | EN | 1 | 3 | 1 | 2 | 131 | 0.64 | 0 |
| Dannehl 2017 | 2017 | 0.57 | 0.35 | PA | 1 | 3 | 1 | 2 | 131 | 0.64 | 0 |
| Dannehl 2017 | 2017 | 1.01 | 0.35 | PN | 1 | 3 | 1 | 2 | 131 | 0.64 | 0 |
| Dannehl 2017 | 2017 | 0.17 | 0.34 | SA | 1 | 3 | 1 | 2 | 131 | 0.64 | 0 |
| Daviss 2009 | 2009 | 1.42 | 0.49 | PA | 0 | 6 | 2 | 1 | 104 | 0.37 | 1 |
| Daviss 2009 | 2009 | 1.04 | 0.82 | SA | 0 | 6 | 2 | 1 | 104 | 0.37 | 1 |
| Dennis 2009 | 2009 | -0.08 | 0.54 | SA | 0 | 6 | 3 | 1 | 148 | 1.00 | 1 |
| Deyessa 2009 | 2009 | 0.84 | 0.29 | SA | 0 | 5 | 4 | 5 | 1994 | 1.00 | 0 |
| Dhamauanti 2020 | 2020 | 1.19 | 0.41 | EA | 0 | 6 | 3 | 3 | 786 | 0.44 | 0 |
| Dinwiddie 2000f | 2000 | 0.79 | 0.14 | SA | 0 | 6 | 3 | 1 | 3868 | 1.00 | 1 |
| Dinwiddie 2000m | 2000 | 1.37 | 0.29 | SA | 0 | 6 | 3 | 1 | 2078 | 0.00 | 1 |
| Douglas 2012 | 2012 | 1.99 | 0.38 | EA | 1 | 3 | 1 | 1 | 105 | 0.63 | 1 |
| Douglas 2012 | 2012 | 2.15 | 0.38 | EN | 1 | 3 | 1 | 1 | 105 | 0.63 | 1 |
| Douglas 2012 | 2012 | 1.24 | 0.36 | PA | 1 | 3 | 1 | 1 | 105 | 0.63 | 1 |
| Douglas 2012 | 2012 | 1.25 | 0.36 | PN | 1 | 3 | 1 | 1 | 105 | 0.63 | 1 |
| Douglas 2012 | 2012 | 1.39 | 0.37 | SA | 1 | 3 | 1 | 1 | 105 | 0.63 | 1 |
| Du 2016 | 2016 | 2.36 | 0.69 | EA | 1 | 3 | 1 | 3 | 36 | 0.58 | 0 |
| Du 2016 | 2016 | 1.60 | 0.65 | EN | 1 | 3 | 1 | 3 | 36 | 0.58 | 0 |
| Du 2016 | 2016 | 1.91 | 0.66 | PA | 1 | 3 | 1 | 3 | 36 | 0.58 | 0 |
| Du 2016 | 2016 | 1.92 | 0.66 | PN | 1 | 3 | 1 | 3 | 36 | 0.58 | 0 |
| Du 2016 | 2016 | 0.90 | 0.63 | SA | 1 | 3 | 1 | 3 | 36 | 0.58 | 0 |
| Dunn 2012 | 2012 | 0.78 | 0.21 | PA | 0 | 6 | 4 | 1 | 5498 | 0.58 | 1 |
| Dunn 2012 | 2012 | 1.14 | 0.15 | SA | 0 | 6 | 4 | 1 | 5498 | 0.58 | 1 |
| Dunn 2013 | 2013 | 0.39 | 0.10 | PA | 0 | 6 | 4 | 1 | 14322 | 0.53 | 1 |
| Dunn 2013 | 2013 | 0.67 | 0.20 | SA | 0 | 6 | 4 | 1 | 14322 | 0.53 | 1 |
| Duran 2004 | 2004 | 1.33 | 0.36 | PA | 0 | 3 | 3 | 1 | 234 | 1.00 | 1 |
| Duran 2004 | 2004 | 1.11 | 0.35 | SA | 0 | 3 | 3 | 1 | 234 | 1.00 | 1 |
| Ege 2015 | 2015 | 0.74 | 0.10 | EA | 1 | 1 | 4 | 1 | 8051 | 0.53 | 1 |
| Ege 2015 | 2015 | 1.22 | 0.10 | PA | 1 | 1 | 4 | 1 | 8051 | 0.53 | 1 |
| Ege 2015 | 2015 | 1.31 | 0.08 | SA | 1 | 1 | 4 | 1 | 8051 | 0.53 | 1 |
| Elm 2020 | 2020 | 0.80 | 0.39 | EA | 1 | 1 | 3 | 1 | 192 | 0.56 | 1 |
| Elm 2020 | 2020 | 1.40 | 0.48 | PA | 1 | 1 | 3 | 1 | 192 | 0.56 | 1 |
| Elm 2020 | 2020 | 1.11 | 0.41 | SA | 1 | 1 | 3 | 1 | 192 | 0.56 | 1 |
| Ernst 1993 | 1993 | 0.74 | 0.43 | SA | 0 | 5 | 3 | 2 | 591 | 0.51 | 0 |
| Fergusson 1996 | 1996 | 1.10 | 0.38 | SA | 0 | 5 | 4 | 1 | 1019 | . | 1 |
| Fergusson 2008 | 2008 | 0.73 | 0.10 | PA | 0 | 5 | 4 | 1 | 1025 | . | 1 |
| Fergusson 2008 | 2008 | 1.40 | 0.11 | SA | 0 | 5 | 4 | 1 | 1025 | . | 1 |
| Fergusson 2013 | 2013 | 1.37 | 0.20 | SA | 0 | 5 | 4 | 1 | 984 | 0.52 | 1 |
| Fernando 2014 | 2014 | 1.47 | 0.36 | EA | 1 | 3 | 1 | 2 | 160 | 0.69 | 0 |
| Fernando 2014 | 2014 | 1.53 | 0.36 | EN | 1 | 3 | 1 | 2 | 160 | 0.69 | 0 |
| Fernando 2014 | 2014 | 0.61 | 0.35 | PA | 1 | 3 | 1 | 2 | 160 | 0.69 | 0 |
| Fernando 2014 | 2014 | 0.77 | 0.35 | PN | 1 | 3 | 1 | 2 | 160 | 0.69 | 0 |
| Fernando 2014 | 2014 | 0.43 | 0.35 | SA | 1 | 3 | 1 | 2 | 160 | 0.69 | 0 |
| Figueroa 1997 | 1997 | 0.63 | 0.84 | SA | 0 | 6 | 1 | 1 | 78 | 0.72 | 1 |
| Fisher 2011 | 2011 | -0.15 | 0.38 | PA | 0 | 2 | 2 | 1 | 151 | . | 1 |
| Fisher 2011 | 2011 | 0.33 | 0.41 | SA | 0 | 2 | 2 | 1 | 151 | . | 1 |
| Fisher 2013 | 2013 | 2.06 | 0.30 | EA | 1 | 3 | 1 | 1 | 455 | 0.68 | 1 |
| Fisher 2013 | 2013 | 1.87 | 0.28 | EN | 1 | 3 | 1 | 1 | 455 | 0.68 | 1 |
| Fisher 2013 | 2013 | 1.43 | 0.41 | PA | 1 | 3 | 1 | 1 | 455 | 0.68 | 1 |
| Fisher 2013 | 2013 | 1.59 | 0.32 | PN | 1 | 3 | 1 | 1 | 455 | 0.68 | 1 |
| Fisher 2013 | 2013 | 1.20 | 0.32 | SA | 1 | 3 | 1 | 1 | 455 | 0.68 | 1 |
| Flisher 1997 | 1997 | 1.34 | 0.40 | PA | 0 | 6 | 4 | 1 | 665 | 0.52 | 1 |
| Friedman 2002 | 2002 | 0.92 | 0.27 | PA | 0 | 6 | 2 | 1 | 201 | 0.70 | 1 |
| Friedman 2002 | 2002 | 1.12 | 0.27 | SA | 0 | 6 | 2 | 1 | 201 | 0.70 | 1 |
| Frodl 2017 | 2017 | 1.68 | 0.07 | EA | 1 | 3 | 1 | 2 | 3036 | 0.53 | . |
| Frodl 2017 | 2017 | 1.40 | 0.07 | EN | 1 | 3 | 1 | 2 | 3036 | 0.53 | . |
| Frodl 2017 | 2017 | 0.98 | 0.07 | PA | 1 | 3 | 1 | 2 | 3036 | 0.53 | . |
| Frodl 2017 | 2017 | 0.88 | 0.07 | PN | 1 | 3 | 1 | 2 | 3036 | 0.53 | . |
| Frodl 2017 | 2017 | 0.72 | 0.07 | SA | 1 | 3 | 1 | 2 | 3036 | 0.53 | . |
| Gallo 2017 | 2017 | 0.99 | 0.18 | EA | 0 | 5 | 3 | 4 | 3715 | 0.53 | 0 |
| Garabedian 2011 | 2011 | 0.60 | 0.09 | PA | 0 | 6 | 3 | 1 | 5380 | 1.00 | 1 |
| Garabedian 2011 | 2011 | 0.71 | 0.12 | SA | 0 | 6 | 3 | 1 | 5380 | 1.00 | 1 |
| Gibb 2007 | 2007 | 0.51 | 0.13 | EA | 1 | 3 | 2 | 1 | 857 | 0.60 | 1 |
| Gibb 2007 | 2007 | 0.25 | 0.13 | PA | 1 | 3 | 2 | 1 | 857 | 0.60 | 1 |
| Gibb 2007 | 2007 | 0.18 | 0.12 | SA | 1 | 3 | 2 | 1 | 857 | 0.60 | 1 |
| Gonzalez 2012 | 2012 | 1.21 | 0.27 | PA | 0 | 6 | 4 | 1 | 1475 | . | 1 |
| Gonzalez 2012 | 2012 | 0.69 | 0.42 | SA | 0 | 6 | 4 | 1 | 1475 | . | 1 |
| Grant 2014 | 2014 | 2.42 | 0.64 | EA | 1 | 3 | 1 | 1 | 39 | 0.54 | 1 |
| Grant 2014 | 2014 | 2.64 | 0.65 | EN | 1 | 3 | 1 | 1 | 39 | 0.54 | 1 |
| Grant 2014 | 2014 | 2.51 | 0.65 | PA | 1 | 3 | 1 | 1 | 39 | 0.54 | 1 |
| Grant 2014 | 2014 | 2.14 | 0.63 | PN | 1 | 3 | 1 | 1 | 39 | 0.54 | 1 |
| Grant 2014 | 2014 | 1.47 | 0.60 | SA | 1 | 3 | 1 | 1 | 39 | 0.54 | 1 |
| Grassi-Oliveira 2011 | 2011 | 2.01 | 0.69 | EA | 1 | 3 | 1 | 4 | 42 | 1.00 | 0 |
| Grassi-Oliveira 2011 | 2011 | 3.86 | 0.78 | EN | 1 | 3 | 1 | 4 | 42 | 1.00 | 0 |
| Grassi-Oliveira 2011 | 2011 | 0.96 | 0.67 | PA | 1 | 3 | 1 | 4 | 42 | 1.00 | 0 |
| Grassi-Oliveira 2011 | 2011 | 1.23 | 0.67 | PN | 1 | 3 | 1 | 4 | 42 | 1.00 | 0 |
| Grassi-Oliveira 2011 | 2011 | 1.39 | 0.67 | SA | 1 | 3 | 1 | 4 | 42 | 1.00 | 0 |
| Greger 2015 | 2015 | 0.62 | 0.26 | PA | 0 | 6 | 3 | 2 | 335 | 0.59 | 0 |
| Greger 2015 | 2015 | 1.27 | 0.27 | SA | 0 | 6 | 3 | 2 | 335 | 0.59 | 0 |
| Grosse 2016 | 2016 | 0.86 | 0.19 | EA | 1 | 3 | 1 | 2 | 394 | 0.59 | 0 |
| Grosse 2016 | 2016 | 1.98 | 0.20 | EN | 1 | 3 | 1 | 2 | 394 | 0.59 | 0 |
| Grosse 2016 | 2016 | 0.51 | 0.18 | PA | 1 | 3 | 1 | 2 | 394 | 0.59 | 0 |
| Grosse 2016 | 2016 | 0.60 | 0.18 | PN | 1 | 3 | 1 | 2 | 394 | 0.59 | 0 |
| Grosse 2016 | 2016 | 0.70 | 0.19 | SA | 1 | 3 | 1 | 2 | 394 | 0.59 | 0 |
| Güleç 2013 | 2013 | 1.79 | 0.33 | EA | 1 | 3 | 1 | 5 | 150 | 0.71 | 0 |
| Güleç 2013 | 2013 | 1.63 | 0.33 | EN | 1 | 3 | 1 | 5 | 150 | 0.71 | 0 |
| Güleç 2013 | 2013 | 0.96 | 0.32 | PA | 1 | 3 | 1 | 5 | 150 | 0.71 | 0 |
| Güleç 2013 | 2013 | 0.55 | 0.32 | PN | 1 | 3 | 1 | 5 | 150 | 0.71 | 0 |
| Güleç 2013 | 2013 | 0.85 | 0.32 | SA | 1 | 3 | 1 | 5 | 150 | 0.71 | 0 |
| Haj-yahia 2001 | 2001 | 0.31 | 0.06 | SA | 0 | 6 | 3 | 2 | 652 | 0.60 | 0 |
| Hanson 2008 | 2008 | 0.85 | 0.14 | PA | 0 | 5 | 4 | 1 | 3906 | 0.49 | 1 |
| Hanson 2008 | 2008 | 0.90 | 0.14 | SA | 0 | 5 | 4 | 1 | 3906 | 0.49 | 1 |
| Harrop-griffiths 1988 | 1988 | 1.34 | 0.65 | SA | 0 | 5 | 2 | 1 | 55 | 1.00 | 1 |
| Hauer 2008 | 2008 | 1.39 | 0.37 | SA | 0 | 5 | 3 | 1 | 133 | 0.81 | 1 |
| He 2019 | 2019 | 1.31 | 0.08 | EA | 1 | 3 | 1 | 3 | 3648 | 0.43 | 0 |
| He 2019 | 2019 | 0.25 | 0.06 | EN | 1 | 3 | 1 | 3 | 3648 | 0.43 | 0 |
| He 2019 | 2019 | 1.14 | 0.06 | PA | 1 | 3 | 1 | 3 | 3648 | 0.43 | 0 |
| He 2019 | 2019 | 0.44 | 0.07 | PN | 1 | 3 | 1 | 3 | 3648 | 0.43 | 0 |
| He 2019 | 2019 | 0.11 | 0.06 | SA | 1 | 3 | 1 | 3 | 3648 | 0.43 | 0 |
| Henny 2007 | 2007 | 0.81 | 0.17 | PA | 0 | 5 | 3 | 1 | 644 | 0.30 | 1 |
| Henny 2007 | 2007 | 0.75 | 0.19 | SA | 0 | 5 | 3 | 1 | 644 | 0.30 | 1 |
| Hill 2000 | 2000 | 1.35 | 0.20 | SA | 0 | 2 | 3 | 1 | 198 | 1.00 | 1 |
| Hill 2001 | 2001 | 1.81 | 0.39 | SA | 0 | 6 | 3 | 1 | 862 | 1.00 | 1 |
| Horesh 2003 | 2003 | 0.00 | 0.33 | SA | 0 | 6 | 2 | 2 | 40 | 0.55 | 0 |
| Hovens 2012 | 2012 | 0.85 | 0.20 | EA | 1 | 6 | 2 | 2 | 1209 | 0.66 | 0 |
| Hovens 2012 | 2012 | 0.68 | 0.16 | EN | 1 | 6 | 2 | 2 | 1209 | 0.66 | 0 |
| Hovens 2012 | 2012 | 0.75 | 0.58 | PA | 1 | 6 | 2 | 2 | 1209 | 0.66 | 0 |
| Hovens 2012 | 2012 | 0.14 | 0.19 | SA | 1 | 6 | 2 | 2 | 1209 | 0.66 | 0 |
| Hovens 2015 | 2015 | -0.58 | 0.56 | EA | 1 | 6 | 3 | 2 | 1167 | 0.66 | 0 |
| Hovens 2015 | 2015 | 0.85 | 0.60 | PA | 1 | 6 | 3 | 2 | 1167 | 0.66 | 0 |
| Hovens 2015 | 2015 | 0.56 | 0.36 | SA | 1 | 6 | 3 | 2 | 1167 | 0.66 | 0 |
| Hughes 1988 | 1988 | 0.52 | 0.40 | EA | 0 | 6 | 1 | 1 | 58 | . | 1 |
| Hughes 1988 | 1988 | -0.07 | 0.57 | PA | 0 | 6 | 1 | 1 | 58 | . | 1 |
| Huu 2017 | 2017 | 0.83 | 0.16 | EA | 1 | 1 | 3 | 3 | 3240 | 0.63 | 0 |
| Huu 2017 | 2017 | 0.05 | 0.16 | PA | 1 | 1 | 3 | 3 | 3240 | 0.63 | 0 |
| Huu 2017 | 2017 | 0.48 | 0.13 | SA | 1 | 1 | 3 | 3 | 3240 | 0.63 | 0 |
| Jaffee 2002 | 2002 | 0.39 | 0.38 | SA | 0 | 5 | 4 | 1 | 998 | 0.48 | 1 |
| Jaschek 2016 | 2016 | 1.75 | 0.16 | PA | 0 | 6 | 4 | 1 | 1041 | . | 1 |
| Jaschek 2016 | 2016 | 1.46 | 0.16 | SA | 0 | 6 | 4 | 1 | 1041 | . | 1 |
| Jewkes 2010 | 2010 | -0.15 | 0.29 | PA | 0 | 3 | 3 | 2 | 1415 | 0.51 | 1 |
| Jewkes 2010 | 2010 | 0.77 | 0.24 | SA | 0 | 3 | 3 | 2 | 1415 | 0.51 | 1 |
| Jobst 2015 | 2015 | 2.58 | 0.66 | EA | 1 | 3 | 1 | 2 | 38 | 0.32 | 0 |
| Jobst 2015 | 2015 | 1.94 | 0.63 | EN | 1 | 3 | 1 | 2 | 38 | 0.32 | 0 |
| Jobst 2015 | 2015 | 1.04 | 0.60 | PA | 1 | 3 | 1 | 2 | 38 | 0.32 | 0 |
| Jobst 2015 | 2015 | 0.56 | 0.59 | PN | 1 | 3 | 1 | 2 | 38 | 0.32 | 0 |
| Jobst 2015 | 2015 | 0.64 | 0.59 | SA | 1 | 3 | 1 | 2 | 38 | 0.32 | 0 |
| Johnson 2010 | 2010 | 1.23 | 0.70 | PA | 0 | 3 | 1 | 1 | 86 | 1.00 | 1 |
| Jonas 2011 | 2011 | 0.55 | 0.07 | SA | 0 | 5 | 4 | 1 | 7353 | . | 1 |
| Kaplan 1998 | 1998 | 1.95 | 0.89 | PA | 0 | 6 | 3 | 1 | 198 | 0.50 | 1 |
| Kaufman 1991 | 1991 | 1.23 | 0.56 | EA | 1 | 6 | 3 | 1 | 56 | 0.52 | 1 |
| Kaufman 1991 | 1991 | 1.58 | 0.57 | EN | 1 | 6 | 3 | 1 | 56 | 0.52 | 1 |
| Kaufman 1991 | 1991 | 1.14 | 0.56 | PA | 1 | 6 | 3 | 1 | 56 | 0.52 | 1 |
| Kaufman 1991 | 1991 | 0.34 | 0.55 | PN | 1 | 6 | 3 | 1 | 56 | 0.52 | 1 |
| Kaufman 1991 | 1991 | -0.40 | 0.55 | SA | 1 | 6 | 3 | 1 | 56 | 0.52 | 1 |
| Kazdin 1985 | 1985 | 1.93 | 0.64 | PA | 0 | 5 | 2 | 1 | 79 | . | 1 |
| Kendler 2000 | 2000 | 0.54 | 0.14 | SA | 0 | 5 | 3 | 1 | 1411 | 1.00 | 1 |
| Kiliç 2017 | 2017 | 1.69 | 0.40 | EA | 1 | 3 | 1 | 5 | 207 | 0.56 | 0 |
| Kiliç 2017 | 2017 | 1.41 | 0.40 | EN | 1 | 3 | 1 | 5 | 207 | 0.56 | 0 |
| Kiliç 2017 | 2017 | 0.85 | 0.39 | PA | 1 | 3 | 1 | 5 | 207 | 0.56 | 0 |
| Kiliç 2017 | 2017 | 1.03 | 0.39 | PN | 1 | 3 | 1 | 5 | 207 | 0.56 | 0 |
| Kiliç 2017 | 2017 | 0.13 | 0.38 | SA | 1 | 3 | 1 | 5 | 207 | 0.56 | 0 |
| Kilpatrick 2003 | 2003 | 0.77 | 0.16 | PA | 0 | 5 | 4 | 1 | 3906 | 0.49 | 1 |
| Kilpatrick 2003 | 2003 | -0.01 | 0.21 | SA | 0 | 5 | 4 | 1 | 3906 | 0.49 | 1 |
| Kim 2005 | 2005 | 3.06 | 0.34 | SA | 0 | 5 | 3 | 3 | 1672 | . | 0 |
| Kim 2017 | 2017 | 1.29 | 0.26 | EA | 1 | 1 | 3 | 3 | 939 | 0.50 | 0 |
| Kim 2017 | 2017 | 1.07 | 0.21 | EN | 1 | 1 | 3 | 3 | 939 | 0.50 | 0 |
| Kim 2017 | 2017 | 0.80 | 0.33 | PA | 1 | 1 | 3 | 3 | 939 | 0.50 | 0 |
| Kim 2017 | 2017 | 0.91 | 0.63 | PN | 1 | 1 | 3 | 3 | 939 | 0.50 | 0 |
| Kim 2017 | 2017 | 0.65 | 0.24 | SA | 1 | 1 | 3 | 3 | 939 | 0.50 | 0 |
| Klein 2016 | 2016 | 2.16 | 0.62 | EA | 1 | 6 | 2 | 1 | 142 | 0.71 | 1 |
| Klein 2016 | 2016 | 1.79 | 0.60 | EN | 1 | 6 | 2 | 1 | 142 | 0.71 | 1 |
| Klein 2016 | 2016 | 0.99 | 0.58 | PA | 1 | 6 | 2 | 1 | 142 | 0.71 | 1 |
| Klein 2016 | 2016 | 1.64 | 0.60 | PN | 1 | 6 | 2 | 1 | 142 | 0.71 | 1 |
| Klein 2016 | 2016 | 1.14 | 0.59 | SA | 1 | 6 | 2 | 1 | 142 | 0.71 | 1 |
| Klein 2013 | 2013 | 0.33 | 0.30 | PA | 0 | 3 | 4 | 1 | 502 | 0.50 | 1 |
| Klein 2013 | 2013 | 0.32 | 0.29 | SA | 0 | 3 | 4 | 1 | 502 | 0.50 | 1 |
| Kolko 1988 | 1988 | -0.65 | 0.67 | PA | 0 | 5 | 2 | 1 | 103 | 0.27 | 1 |
| Kolko 1988 | 1988 | 0.17 | 0.66 | SA | 0 | 5 | 2 | 1 | 103 | 0.27 | 1 |
| Kosseva 2010 | 2010 | 0.87 | 0.34 | EA | 1 | 3 | 2 | 2 | 293 | 0.86 | 0 |
| Kosseva 2010 | 2010 | 1.30 | 0.32 | EN | 1 | 3 | 2 | 2 | 293 | 0.86 | 0 |
| Kosseva 2010 | 2010 | 0.70 | 0.38 | PA | 1 | 3 | 2 | 2 | 293 | 0.86 | 0 |
| Kosseva 2010 | 2010 | 1.34 | 0.32 | PN | 1 | 3 | 2 | 2 | 293 | 0.86 | 0 |
| Kosseva 2010 | 2010 | 0.30 | 0.35 | SA | 1 | 3 | 2 | 2 | 293 | 0.86 | 0 |
| Kounou 2013 | 2013 | 1.73 | 0.38 | EA | 1 | 3 | 1 | 3 | 181 | 0.66 | 0 |
| Kounou 2013 | 2013 | 1.03 | 0.31 | EN | 1 | 3 | 1 | 3 | 181 | 0.66 | 0 |
| Kounou 2013 | 2013 | 0.87 | 0.35 | PA | 1 | 3 | 1 | 3 | 181 | 0.66 | 0 |
| Kounou 2013 | 2013 | 1.10 | 0.33 | PN | 1 | 3 | 1 | 3 | 181 | 0.66 | 0 |
| Kounou 2013 | 2013 | 1.30 | 0.32 | SA | 1 | 3 | 1 | 3 | 181 | 0.66 | 0 |
| Kuhlman 2013 | 2013 | 0.66 | 0.02 | EA | 1 | 3 | 3 | 2 | 141 | 0.41 | 0 |
| Kuhlman 2013 | 2013 | 0.08 | 0.01 | EN | 1 | 3 | 3 | 2 | 141 | 0.41 | 0 |
| Kuhlman 2013 | 2013 | 0.49 | 0.02 | PA | 1 | 3 | 3 | 2 | 141 | 0.41 | 0 |
| Kuhlman 2013 | 2013 | 0.30 | 0.02 | PN | 1 | 3 | 3 | 2 | 141 | 0.41 | 0 |
| Kuhlman 2013 | 2013 | 0.23 | 0.02 | SA | 1 | 3 | 3 | 2 | 141 | 0.41 | 0 |
| Kuyken 2006 | 2006 | 3.33 | 1.47 | SA | 0 | 6 | 3 | 1 | 62 | 0.81 | 1 |
| Lang, Huang 2006 | 2006 | 1.06 | 0.30 | EA | 1 | 3 | 2 | 3 | 202 | 0.62 | 0 |
| Lang, Huang 2006 | 2006 | 0.25 | 0.26 | EN | 1 | 3 | 2 | 3 | 202 | 0.62 | 0 |
| Lang, Huang 2006 | 2006 | 0.18 | 0.25 | PA | 1 | 3 | 2 | 3 | 202 | 0.62 | 0 |
| Lang, Huang 2006 | 2006 | 2.09 | 0.41 | SA | 1 | 3 | 2 | 3 | 202 | 0.62 | 0 |
| Lenze 2013 | 2013 | 2.11 | 0.53 | EA | 1 | 2 | 1 | 1 | 55 | 1.00 | 1 |
| Lenze 2013 | 2013 | 1.67 | 0.52 | PA | 1 | 2 | 1 | 1 | 55 | 1.00 | 1 |
| Lenze 2013 | 2013 | 1.26 | 0.51 | SA | 1 | 2 | 1 | 1 | 55 | 1.00 | 1 |
| Lewis 2003 | 2003 | 0.71 | 0.35 | SA | 0 | 6 | 3 | 1 | 255 | 1.00 | 1 |
| Li 2020 | 2020 | 0.17 | 0.05 | PA | 0 | 5 | 4 | 3 | 7283 | . | 0 |
| Libby 2005 north | 2005 | 1.26 | 0.90 | PA | 0 | 5 | 4 | 1 | 1638 | 0.52 | 1 |
| Libby 2005 north | 2005 | 1.28 | 1.32 | SA | 0 | 5 | 4 | 1 | 1638 | 0.52 | 1 |
| Libby 2005 south | 2005 | 1.58 | 1.21 | PA | 0 | 5 | 4 | 1 | 1446 | 0.57 | 1 |
| Libby 2005 south | 2005 | 1.53 | 1.32 | SA | 0 | 5 | 4 | 1 | 1446 | 0.57 | 1 |
| Ling 2009 | 2009 | 1.36 | 0.11 | EA | 0 |  | 3 | 3 | 1219 | 0.49 | 0 |
| Ling 2009 | 2009 | 1.06 | 0.11 | EN | 0 |  | 3 | 3 | 1219 | 0.49 | 0 |
| Ling 2009 | 2009 | 0.82 | 0.11 | PA | 0 |  | 3 | 3 | 1219 | 0.49 | 0 |
| Ling 2009 | 2009 | 0.94 | 0.11 | PN | 0 |  | 3 | 3 | 1219 | 0.49 | 0 |
| Liu 2021 | 2021 | 1.99 | 0.16 | EA | 1 | 3 | 4 | 3 | 715 | 0.52 | 0 |
| Liu 2021 | 2021 | 0.73 | 0.14 | EN | 1 | 3 | 4 | 3 | 715 | 0.52 | 0 |
| Liu 2021 | 2021 | 1.19 | 0.14 | PA | 1 | 3 | 4 | 3 | 715 | 0.52 | 0 |
| Liu 2021 | 2021 | 1.08 | 0.14 | PN | 1 | 3 | 4 | 3 | 715 | 0.52 | 0 |
| Liu 2021 | 2021 | 1.17 | 0.14 | SA | 1 | 3 | 4 | 3 | 715 | 0.52 | 0 |
| Lizardi 1995 | 1995 | 0.83 | 0.52 | PA | 0 | 6 | 1 | 1 | 187 | 0.75 | 1 |
| Lizardi 1995 | 1995 | 1.62 | 0.97 | SA | 0 | 6 | 1 | 1 | 187 | 0.75 | 1 |
| Lu 2016 | 2016 | 0.79 | 0.41 | EA | 1 | 3 | 1 | 3 | 80 | 0.58 | 0 |
| Lu 2016 | 2016 | 0.78 | 0.41 | EN | 1 | 3 | 1 | 3 | 80 | 0.58 | 0 |
| Lu 2016 | 2016 | 0.21 | 0.41 | PA | 1 | 3 | 1 | 3 | 80 | 0.58 | 0 |
| Lu 2016 | 2016 | 1.14 | 0.42 | PN | 1 | 3 | 1 | 3 | 80 | 0.58 | 0 |
| Lu 2016 | 2016 | 0.32 | 0.41 | SA | 1 | 3 | 1 | 3 | 80 | 0.58 | 0 |
| Lu 2018 | 2018 | 0.00 | 0.28 | EA | 1 | 3 | 2 | 3 | 168 |  | 0 |
| Lu 2018 | 2018 | 0.11 | 0.28 | EN | 1 | 3 | 2 | 3 | 168 |  | 0 |
| Lu 2018 | 2018 | 0.25 | 0.29 | PA | 1 | 3 | 2 | 3 | 168 |  | 0 |
| Lu 2018 | 2018 | -0.07 | 0.29 | PN | 1 | 3 | 2 | 3 | 168 |  | 0 |
| Lu 2018 | 2018 | 0.00 | 0.28 | SA | 1 | 3 | 2 | 3 | 168 |  | 0 |
| Mac Giollabhui 2018 | 2018 | 0.59 | 0.08 | EA | 0 | 6 | 3 | 1 | 173 | 0.56 | 1 |
| Maciejewski 2006 | 2006 | 1.34 | 0.67 | EA | 1 | 6 | 1 | 1 | 50 | 0.48 | 1 |
| Maciejewski 2006 | 2006 | 0.76 | 0.62 | PA | 1 | 6 | 1 | 1 | 50 | 0.48 | 1 |
| Maciejewski 2006 | 2006 | 0.51 | 0.61 | SA | 1 | 6 | 1 | 1 | 50 | 0.48 | 1 |
| MacMillan 2001f | 2001 | 1.17 | 0.11 | PA | 0 | 5 | 4 | 1 | 3678 | 1.00 | 1 |
| MacMillan 2001f | 2001 | 1.36 | 0.12 | SA | 0 | 5 | 4 | 1 | 3678 | 1.00 | 1 |
| MacMillan 2001m | 2001 | 0.48 | 0.16 | PA | 0 | 5 | 4 | 1 | 3338 | 0.00 | 1 |
| MacMillan 2001m | 2001 | 0.52 | 0.31 | SA | 0 | 5 | 4 | 1 | 3338 | 0.00 | 1 |
| Mall 2018 | 2018 | 1.16 | 0.23 | EA | 1 | 1 | 3 | 2 | 686 | 0.55 | 1 |
| Mall 2018 | 2018 | 0.59 | 0.26 | PA | 1 | 1 | 3 | 2 | 686 | 0.55 | 1 |
| Mall 2018 | 2018 | 1.10 | 0.43 | SA | 1 | 1 | 3 | 2 | 686 | 0.55 | 1 |
| Malykhin 2010 | 2010 | 2.14 | 0.46 | EA | 1 | 3 | 1 | 1 | 73 | 0.77 | 1 |
| Malykhin 2010 | 2010 | 2.23 | 0.46 | EN | 1 | 3 | 1 | 1 | 73 | 0.77 | 1 |
| Malykhin 2010 | 2010 | 0.88 | 0.43 | PA | 1 | 3 | 1 | 1 | 73 | 0.77 | 1 |
| Malykhin 2010 | 2010 | 2.30 | 0.47 | PN | 1 | 3 | 1 | 1 | 73 | 0.77 | 1 |
| Malykhin 2010 | 2010 | 0.68 | 0.43 | SA | 1 | 3 | 1 | 1 | 73 | 0.77 | 1 |
| Mannarino 1991 | 1991 | 0.48 | 0.28 | SA | 0 | 6 | 1 | 1 | 169 | 1.00 | 1 |
| Mansbach-Kleinfeld 2015 | 2015 | 1.50 | 0.62 | SA | 0 | 5 | 4 | 2 | 906 | 0.50 | 0 |
| Mccutcheon 2009 | 2009 | 0.40 | 0.05 | PA | 0 | 6 | 3 | 1 | 5266 | 0.56 | 1 |
| Mccutcheon 2009 | 2009 | 0.45 | 0.07 | SA | 0 | 6 | 3 | 1 | 5266 | 0.56 | 1 |
| Mchichi 2004 | 2004 | 0.62 | 0.27 | SA | 0 | 5 | 4 | 5 | 728 | 1.00 | 0 |
| McLeer 1994 | 1994 | -0.58 | 0.98 | SA | 0 | 6 | 2 | 1 | 49 | 0.51 | 1 |
| Mikaeili 2013 | 2013 | 0.18 | 0.12 | EA | 0 | 3 | 4 | 5 | 893 | 0.00 | 0 |
| Mikaeili 2013 | 2013 | 0.25 | 0.12 | PA | 0 | 3 | 4 | 5 | 893 | 0.00 | 0 |
| Miller 2009 | 2009 | 1.74 | 0.51 | PA | 0 | 5 | 1 | 1 | 66 | 0.58 | 1 |
| Miller 2009 | 2009 | 2.19 | 0.85 | SA | 0 | 5 | 1 | 1 | 66 | 0.58 | 1 |
| Molnar 2001f | 2001 | 0.59 | 0.13 | SA | 0 | 6 | 4 | 1 | 2921 | 1.00 | 1 |
| Molnar 2001m | 2001 | 0.59 | 0.36 | SA | 0 | 6 | 4 | 1 | 2945 | 0.00 | 1 |
| Morais 2018 | 2018 | 0.52 | 0.19 | SA | 0 | 5 | 3 | 1 | 498 | 0.00 | 1 |
| Mullen 1993 | 1993 | 0.96 | 0.18 | SA | 0 | 6 | 4 | 1 | 492 | 1.00 | 1 |
| Mullen 1996 | 1996 | 1.04 | 0.31 | EA | 1 | 5 | 4 | 1 | 497 | 1.00 | 1 |
| Mullen 1996 | 1996 | 0.94 | 0.36 | PA | 1 | 5 | 4 | 1 | 497 | 1.00 | 1 |
| Mullen 1996 | 1996 | 1.35 | 0.33 | SA | 1 | 5 | 4 | 1 | 497 | 1.00 | 1 |
| Münzer 2016 | 2016 | 2.39 | 0.78 | SA | 0 | 6 | 3 | 2 | 178 | 0.45 | 0 |
| Murphy 2012 | 2012 | 1.67 | 0.40 | EA | 1 | 3 | 1 | 2 | 90 | 0.63 | 1 |
| Murphy 2012 | 2012 | 1.96 | 0.41 | EN | 1 | 3 | 1 | 2 | 90 | 0.63 | 1 |
| Murphy 2012 | 2012 | 1.21 | 0.39 | PA | 1 | 3 | 1 | 2 | 90 | 0.63 | 1 |
| Murphy 2012 | 2012 | 1.48 | 0.40 | PN | 1 | 3 | 1 | 2 | 90 | 0.63 | 1 |
| Murphy 2012 | 2012 | 1.13 | 0.39 | SA | 1 | 3 | 1 | 2 | 90 | 0.63 | 1 |
| Nasreen 2016 | 2016 | 0.10 | 0.26 | PA | 0 | 5 | 4 | 5 | 2440 | 0.50 | 0 |
| Nasreen 2016 | 2016 | 0.77 | 0.34 | SA | 0 | 5 | 4 | 5 | 2440 | 0.50 | 0 |
| Nduna 2013 | 2013 | 0.37 | 0.17 | SA | 0 | 5 | 3 | 2 | 2783 | 0.51 | 1 |
| Nelson 2002 | 2002 | 0.59 | 0.18 | SA | 0 | 5 | 3 | 1 | 3982 | 0.58 | 1 |
| Ng 2011 | 2011 | 1.33 | 0.30 | EA | 1 | 3 | 1 | 3 | 160 | 0.68 | 0 |
| Ng 2011 | 2011 | 0.59 | 0.29 | EN | 1 | 3 | 1 | 3 | 160 | 0.68 | 0 |
| Ng 2011 | 2011 | 0.84 | 0.29 | PA | 1 | 3 | 1 | 3 | 160 | 0.68 | 0 |
| Ng 2011 | 2011 | 1.32 | 0.30 | PN | 1 | 3 | 1 | 3 | 160 | 0.68 | 0 |
| Ng 2011 | 2011 | 0.56 | 0.29 | SA | 1 | 3 | 1 | 3 | 160 | 0.68 | 0 |
| Nicolaidis 2004 | 2004 | 1.40 | 0.35 | PA | 0 | 4 | 3 | 1 | 174 | 1.00 | 1 |
| Novelo 2018 | 2018 | 0.93 | 0.22 | EA | 1 | 3 | 3 | 4 | 449 | 0.64 | 0 |
| Novelo 2018 | 2018 | 1.16 | 0.22 | EN | 1 | 3 | 3 | 4 | 449 | 0.64 | 0 |
| Novelo 2018 | 2018 | 0.62 | 0.21 | PA | 1 | 3 | 3 | 4 | 449 | 0.64 | 0 |
| Novelo 2018 | 2018 | 0.72 | 0.21 | PN | 1 | 3 | 3 | 4 | 449 | 0.64 | 0 |
| Novelo 2018 | 2018 | 0.47 | 0.33 | SA | 1 | 3 | 3 | 4 | 449 | 0.64 | 0 |
| Olsson 1999 | 1999 | 0.54 | 0.75 | PA | 0 | 6 | 1 | 2 | 150 | 0.77 | 0 |
| Opel 2014 | 2014 | 1.50 | 0.29 | EA | 1 | 3 | 1 | 2 | 170 | 0.62 | 0 |
| Opel 2014 | 2014 | 1.56 | 0.29 | EN | 1 | 3 | 1 | 2 | 170 | 0.62 | 0 |
| Opel 2014 | 2014 | 0.79 | 0.28 | PA | 1 | 3 | 1 | 2 | 170 | 0.62 | 0 |
| Opel 2014 | 2014 | 0.61 | 0.28 | PN | 1 | 3 | 1 | 2 | 170 | 0.62 | 0 |
| Opel 2014 | 2014 | 0.92 | 0.28 | SA | 1 | 3 | 1 | 2 | 170 | 0.62 | 0 |
| Opel 2016 | 2016 | 1.41 | 0.49 | EA | 1 | 3 | 1 | 2 | 76 | 0.50 | 0 |
| Opel 2016 | 2016 | 0.97 | 0.48 | EN | 1 | 3 | 1 | 2 | 76 | 0.50 | 0 |
| Opel 2016 | 2016 | 0.81 | 0.48 | PA | 1 | 3 | 1 | 2 | 76 | 0.50 | 0 |
| Opel 2016 | 2016 | 0.84 | 0.48 | PN | 1 | 3 | 1 | 2 | 76 | 0.50 | 0 |
| Opel 2016 | 2016 | 0.52 | 0.47 | SA | 1 | 3 | 1 | 2 | 76 | 0.50 | 0 |
| Pantle 1990 | 1990 | 0.00 | 0.54 | SA | 0 | 5 | 2 | 1 | 111 | 1.00 | 1 |
| Paquette 2017 | 2017 | 0.85 | 0.34 | EA | 1 | 6 | 4 | 1 | 621 | 1.00 | 1 |
| Paquette 2017 | 2017 | -0.63 | 0.43 | PA | 1 | 6 | 4 | 1 | 621 | 1.00 | 1 |
| Paquette 2017 | 2017 | 0.97 | 0.29 | SA | 1 | 6 | 4 | 1 | 621 | 1.00 | 1 |
| Pham 2021 | 2021 | 0.77 | 0.40 | EA | 1 | 3 | 2 | 3 | 273 | 0.59 | 0 |
| Pham 2021 | 2021 | 0.96 | 0.45 | EN | 1 | 3 | 2 | 3 | 273 | 0.59 | 0 |
| Pham 2021 | 2021 | 0.29 | 0.44 | PA | 1 | 3 | 2 | 3 | 273 | 0.59 | 0 |
| Pham 2021 | 2021 | -0.34 | 0.47 | PN | 1 | 3 | 2 | 3 | 273 | 0.59 | 0 |
| Pham 2021 | 2021 | 0.29 | 0.40 | SA | 1 | 3 | 2 | 3 | 273 | 0.59 | 0 |
| Pickles 2010 | 2010 | 0.79 | 0.30 | SA | 0 | 5 | 4 | 1 | 2226 | 0.50 | 1 |
| Plaza 2012 | 2012 | 0.99 | 0.45 | EA | 1 | 6 | 3 | 2 | 303 | 1.00 | 0 |
| Plaza 2012 | 2012 | 1.61 | 0.43 | PA | 1 | 6 | 3 | 2 | 303 | 1.00 | 0 |
| Plaza 2012 | 2012 | 0.95 | 0.43 | SA | 1 | 6 | 3 | 2 | 303 | 1.00 | 0 |
| Qu 2022 | 2022 | 1.87 | 0.18 | EA | 0 | 1 | 3 | 3 | 2105 | 0.45 | 0 |
| Qu 2022 | 2022 | 0.78 | 0.08 | EN | 0 | 1 | 3 | 3 | 2105 | 0.45 | 0 |
| Qu 2022 | 2022 | 1.15 | 0.13 | PA | 0 | 1 | 3 | 3 | 2105 | 0.45 | 0 |
| Qu 2022 | 2022 | 0.72 | 0.07 | PN | 0 | 1 | 3 | 3 | 2105 | 0.45 | 0 |
| Raheel 2015 | 2015 | 1.24 | 0.30 | EA | 0 | 5 | 4 | 5 | 1028 | 1.00 | 0 |
| Raheel 2015 | 2015 | 1.21 | 0.29 | PA | 0 | 5 | 4 | 5 | 1028 | 1.00 | 0 |
| Ramos 2004 | 2004 | 2.83 | 0.42 | PA | 0 | 6 | 3 | 1 | 491 | 1.00 | 1 |
| Ramos 2004 | 2004 | 1.60 | 0.32 | SA | 0 | 6 | 3 | 1 | 491 | 1.00 | 1 |
| Remigio-Baker 2014 | 2014 | 1.17 | 0.23 | EA | 1 | 1 | 4 | 1 | 3437 | 1.00 | 1 |
| Remigio-Baker 2014 | 2014 | 0.51 | 0.22 | PA | 1 | 1 | 4 | 1 | 3437 | 1.00 | 1 |
| Remigio-Baker 2014 | 2014 | 0.52 | 0.24 | SA | 1 | 1 | 4 | 1 | 3437 | 1.00 | 1 |
| Ritchie 2009 | 2009 | 1.33 | 0.40 | EA | 0 | 5 | 4 | 2 | 942 | 0.58 | 0 |
| Ritchie 2009 | 2009 | 0.92 | 0.32 | PA | 0 | 5 | 4 | 2 | 942 | 0.58 | 0 |
| Rivera-Rivera 2015 | 2015 | 0.72 | 0.08 | EA | 0 | 5 | 4 | 4 | 9982 | 0.55 | 0 |
| Rizzo 2010 | 2010 | 0.94 | 0.41 | EA | 0 | 6 | 2 | 1 | 155 | 0.76 | 1 |
| Rohde 2008 | 2008 | 0.85 | 0.16 | PA | 0 | 3 | 3 | 1 | 4641 | 1.00 | 1 |
| Rohde 2008 | 2008 | 0.76 | 0.14 | SA | 0 | 3 | 3 | 1 | 4641 | 1.00 | 1 |
| Rorty 1994 | 1994 | 0.39 | 0.60 | EA | 1 | 6 | 3 | 1 | 120 | 1.00 | 1 |
| Rorty 1994 | 1994 | 0.89 | 0.84 | PA | 1 | 6 | 3 | 1 | 120 | 1.00 | 1 |
| Rorty 1994 | 1994 | 0.42 | 0.70 | SA | 1 | 6 | 3 | 1 | 120 | 1.00 | 1 |
| Roustit 2009 | 2009 | 0.29 | 0.28 | PA | 0 | 6 | 1 | 2 | 788 | 0.57 | 0 |
| Roustit 2009 | 2009 | 0.74 | 0.31 | SA | 0 | 6 | 1 | 2 | 788 | 0.57 | 0 |
| Rubino 2009 | 2009 | 1.22 | 0.53 | EA | 1 | 6 | 1 | 2 | 788 | 0.57 | 0 |
| Rubino 2009 | 2009 | 0.55 | 0.49 | PA | 1 | 6 | 1 | 2 | 788 | 0.57 | 0 |
| Rubino 2009 | 2009 | 0.79 | 0.92 | SA | 1 | 6 | 1 | 2 | 788 | 0.57 | 0 |
| Sadowski 2003 | 2003 | 0.96 | 0.40 | SA | 0 | 6 | 2 | 1 | 46 | 1.00 | 1 |
| Satinsky 2021 | 2021 | 1.92 | 0.55 | EA | 1 | 1 | 4 | 5 | 1626 | 0.56 | 1 |
| Satinsky 2021 | 2021 | 0.89 | 0.58 | PA | 1 | 1 | 4 | 5 | 1626 | 0.56 | 1 |
| Satinsky 2021 | 2021 | 0.55 | 0.22 | SA | 1 | 1 | 4 | 5 | 1626 | 0.56 | 1 |
| Saunders 1992 | 1992 | 0.70 | 0.22 | SA | 0 | 6 | 4 | 1 | 391 | 1.00 | 1 |
| Scott 1992 | 1992 | 1.22 | 0.17 | SA | 0 | 6 | . | 1 | 3131 | . | 1 |
| Sedney 1984 | 1984 | 0.88 | 0.41 | SA | 0 | 5 | 3 | 1 | 301 | 1.00 | 1 |
| Shah 2021 | 2021 | 0.99 | 0.24 | EA | 0 | 6 | 4 | 5 | 518 | 0.61 | 0 |
| Shah 2021 | 2021 | 0.52 | 0.32 | PA | 0 | 6 | 4 | 5 | 518 | 0.61 | 0 |
| Silverman 1996f | 1996 | 1.82 | 0.75 | PA | 0 | 5 | 4 | 1 | 187 | 1.00 | 1 |
| Silverman 1996f | 1996 | 1.83 | 0.64 | SA | 0 | 5 | 4 | 1 | 187 | 1.00 | 1 |
| Silverman 1996m | 1996 | 1.81 | 0.88 | PA | 0 | 5 | 4 | 1 | 188 | 1.00 | 1 |
| Skeen 2016 | 2016 | 0.41 | 0.08 | EA | 0 | 6 | 3 | 2 | 989 | 0.51 | 1 |
| Skeen 2016 | 2016 | 0.13 | 0.12 | PA | 0 | 6 | 3 | 2 | 989 | 0.51 | 1 |
| Sonnby 2011f | 2011 | 0.78 | 0.12 | SA | 0 | 5 | 4 | 2 | 2455 | 1.00 | 0 |
| Sonnby 2011m | 2011 | 0.87 | 0.21 | SA | 0 | 5 | 4 | 2 | 2455 | 0.00 | 0 |
| Springer 2007 | 2007 | 0.48 | 0.17 | PA | 0 | 4 | 3 | 1 | 2051 | 0.52 | 1 |
| Stuart 1990 | 1990 | 0.56 | 0.57 | SA | 0 | 6 | 1 | 1 | 145 | 1.00 | 1 |
| Subica 2013 | 2013 | 0.34 | 0.32 | PA | 0 | 6 | 2 | 1 | 172 | 0.45 | 1 |
| Subica 2013 | 2013 | 0.92 | 0.34 | SA | 0 | 6 | 2 | 1 | 172 | 0.45 | 1 |
| Subramaniam 2020 | 2020 | 1.16 | 0.27 | EA | 1 | 1 | 4 | 3 | 4441 | 0.50 | 0 |
| Subramaniam 2020 | 2020 | 0.83 | 0.22 | EN | 1 | 1 | 4 | 3 | 4441 | 0.50 | 0 |
| Subramaniam 2020 | 2020 | 1.28 | 0.30 | PA | 1 | 1 | 4 | 3 | 4441 | 0.50 | 0 |
| Subramaniam 2020 | 2020 | 0.53 | 0.35 | PN | 1 | 1 | 4 | 3 | 4441 | 0.50 | 0 |
| Subramaniam 2020 | 2020 | 0.83 | 0.38 | SA | 1 | 1 | 4 | 3 | 4441 | 0.50 | 0 |
| Sugaya 2012 | 2012 | 0.15 | 0.07 | PA | 0 | 1 | 4 | 1 | 34384 | 0.52 | 1 |
| Suzuki 2014 | 2014 | 1.21 | 0.42 | EA | 1 | 3 | 1 | 1 | 89 | 0.65 | 1 |
| Suzuki 2014 | 2014 | 1.64 | 0.43 | EN | 1 | 3 | 1 | 1 | 89 | 0.65 | 1 |
| Suzuki 2014 | 2014 | -0.40 | 0.41 | PA | 1 | 3 | 1 | 1 | 89 | 0.65 | 1 |
| Suzuki 2014 | 2014 | 0.23 | 0.41 | PN | 1 | 3 | 1 | 1 | 89 | 0.65 | 1 |
| Suzuki 2014 | 2014 | 0.45 | 0.41 | SA | 1 | 3 | 1 | 1 | 89 | 0.65 | 1 |
| Taillieu 2016 | 2016 | 1.24 | 0.10 | EA | 0 | 1 | 4 | 1 | 3426 | 0.58 | 1 |
| Tatham 2016 | 2016 | 1.38 | 0.53 | EA | 1 | 3 | 1 | 1 | 61 | . | 1 |
| Tatham 2016 | 2016 | 3.49 | 0.61 | EN | 1 | 3 | 1 | 1 | 61 | . | 1 |
| Tatham 2016 | 2016 | 1.20 | 0.53 | PA | 1 | 3 | 1 | 1 | 61 | . | 1 |
| Tatham 2016 | 2016 | 8.73 | 0.95 | PN | 1 | 3 | 1 | 1 | 61 | . | 1 |
| Tatham 2016 | 2016 | 0.67 | 0.52 | SA | 1 | 3 | 1 | 1 | 61 | . | 1 |
| Tietjen 2009 | 2009 | 0.94 | 0.12 | EA | 1 | 3 | 2 | 1 | 1348 | 0.88 | 1 |
| Tietjen 2009 | 2009 | 0.86 | 0.12 | EN | 1 | 3 | 2 | 1 | 1348 | 0.88 | 1 |
| Tietjen 2009 | 2009 | 0.66 | 0.14 | PA | 1 | 3 | 2 | 1 | 1348 | 0.88 | 1 |
| Tietjen 2009 | 2009 | 0.78 | 0.14 | PN | 1 | 3 | 2 | 1 | 1348 | 0.88 | 1 |
| Tietjen 2009 | 2009 | 0.67 | 0.13 | SA | 1 | 3 | 2 | 1 | 1348 | 0.88 | 1 |
| Treadway 2009 | 2009 | 2.55 | 0.66 | EA | 1 | 3 | 1 | 1 | 38 | 0.53 | 1 |
| Treadway 2009 | 2009 | 3.06 | 0.68 | EN | 1 | 3 | 1 | 1 | 38 | 0.53 | 1 |
| Treadway 2009 | 2009 | 2.70 | 0.66 | PA | 1 | 3 | 1 | 1 | 38 | 0.53 | 1 |
| Treadway 2009 | 2009 | 2.23 | 0.64 | PN | 1 | 3 | 1 | 1 | 38 | 0.53 | 1 |
| Treadway 2009 | 2009 | 1.86 | 0.63 | SA | 1 | 3 | 1 | 1 | 38 | 0.53 | 1 |
| Turner 2017 | 2017 | 1.17 | 0.21 | SA | 0 | 1 | 4 | 1 | 14564 | 0.00 | 1 |
| Ugwu 2015 | 2015 | 1.96 | 0.40 | EA | 1 | 3 | 1 | 2 | 92 | 0.60 | 1 |
| Ugwu 2015 | 2015 | 2.27 | 0.41 | EN | 1 | 3 | 1 | 2 | 92 | 0.60 | 1 |
| Ugwu 2015 | 2015 | 1.48 | 0.39 | PA | 1 | 3 | 1 | 2 | 92 | 0.60 | 1 |
| Ugwu 2015 | 2015 | 1.53 | 0.39 | PN | 1 | 3 | 1 | 2 | 92 | 0.60 | 1 |
| Ugwu 2015 | 2015 | 1.56 | 0.40 | SA | 1 | 3 | 1 | 2 | 92 | 0.60 | 1 |
| van Assche 2019 | 2019 | 0.66 | 0.03 | EA | 1 | 3 | 3 | 2 | 81 | 0.64 | 0 |
| van Assche 2019 | 2019 | 1.10 | 0.03 | EN | 1 | 3 | 3 | 2 | 81 | 0.64 | 0 |
| van Assche 2019 | 2019 | 0.44 | 0.03 | PA | 1 | 3 | 3 | 2 | 81 | 0.64 | 0 |
| van Assche 2019 | 2019 | 0.59 | 0.03 | PN | 1 | 3 | 3 | 2 | 81 | 0.64 | 0 |
| van Assche 2019 | 2019 | -0.55 | 0.03 | SA | 1 | 3 | 3 | 2 | 81 | 0.64 | 0 |
| Wahab 2013 | 2013 | 1.54 | 1.23 | SA | 0 | 5 | 2 | 3 | 51 | 1.00 | 0 |
| Wainwright 2002 | 2002 | 0.72 | 0.37 | PA | 0 | 6 | 3 | 1 | 3353 | 0.55 | 1 |
| Walker 1988 | 1988 | 1.07 | 0.57 | SA | 0 | 6 | 2 | 1 | 55 | 1.00 | 1 |
| Walker 1992 | 1992 | 1.56 | 0.66 | SA | 0 | 5 | 3 | 1 | 100 | 1.00 | 1 |
| Walton 2011 | 2011 | 0.99 | 0.28 | EA | 1 | 3 | 3 | 1 | 233 | 0.36 | 1 |
| Walton 2011 | 2011 | 1.31 | 0.32 | EN | 1 | 3 | 3 | 1 | 233 | 0.36 | 1 |
| Walton 2011 | 2011 | 0.63 | 0.27 | PA | 1 | 3 | 3 | 1 | 233 | 0.36 | 1 |
| Walton 2011 | 2011 | 0.43 | 0.28 | PN | 1 | 3 | 3 | 1 | 233 | 0.36 | 1 |
| Walton 2011 | 2011 | 0.72 | 0.29 | SA | 1 | 3 | 3 | 1 | 233 | 0.36 | 1 |
| Watson 2007 | 2007 | 2.81 | 0.58 | EA | 1 | 3 | 2 | 1 | 40 | 0.48 | 1 |
| Watson 2007 | 2007 | 3.89 | 0.58 | EN | 1 | 3 | 2 | 1 | 40 | 0.48 | 1 |
| Watson 2007 | 2007 | -0.18 | 0.58 | PA | 1 | 3 | 2 | 1 | 40 | 0.48 | 1 |
| Watson 2007 | 2007 | 2.43 | 0.58 | PN | 1 | 3 | 2 | 1 | 40 | 0.48 | 1 |
| Watson 2007 | 2007 | 2.78 | 0.58 | SA | 1 | 3 | 2 | 1 | 40 | 0.48 | 1 |
| Webster 2000 | 2000 | 1.34 | 0.49 | EA | 1 | 6 | 1 | 1 | 160 | 1.00 | 1 |
| Webster 2000 | 2000 | 1.56 | 0.83 | PA | 1 | 6 | 1 | 1 | 160 | 1.00 | 1 |
| Webster 2000 | 2000 | 0.72 | 0.46 | SA | 1 | 6 | 1 | 1 | 160 | 1.00 | 1 |
| Weeramanthri 2003 | 2003 | 0.96 | 0.40 | SA | 0 | 6 | 2 | 1 | 46 | 1.00 | 1 |
| Wessel 2001 | 2001 | 1.14 | 0.34 | EA | 1 | 3 | 1 | 2 | 117 | 0.54 | 0 |
| Wessel 2001 | 2001 | 1.04 | 0.34 | EN | 1 | 3 | 1 | 2 | 117 | 0.54 | 0 |
| Wessel 2001 | 2001 | 0.75 | 0.34 | PA | 1 | 3 | 1 | 2 | 117 | 0.54 | 0 |
| Wessel 2001 | 2001 | 0.13 | 0.34 | PN | 1 | 3 | 1 | 2 | 117 | 0.54 | 0 |
| Wessel 2001 | 2001 | 0.33 | 0.34 | SA | 1 | 3 | 1 | 2 | 117 | 0.54 | 0 |
| Widom 2007 | 2007 | 0.29 | 0.31 | PA | 0 | 6 | 1 | 1 | 1196 | 0.49 | 1 |
| Widom 2007 | 2007 | 0.09 | 0.35 | SA | 0 | 6 | 1 | 1 | 1196 | 0.49 | 1 |
| Wilsnack 1996 | 1996 | 0.97 | 0.15 | SA | 0 | 6 | 4 | 1 | 1099 | 1.00 | 1 |
| Wilson 2014 | 2014 | 2.34 | 0.79 | PA | 0 | 6 | 3 | 1 | 1698 | 0.54 | 1 |
| Wilson 2014 | 2014 | 0.66 | 0.50 | SA | 0 | 6 | 3 | 1 | 1698 | 0.54 | 1 |
| Windle 1995f | 1995 | -0.05 | 0.34 | PA | 0 | 5 | 2 | 1 | 321 | 1.00 | 1 |
| Windle 1995f | 1995 | -0.02 | 0.30 | SA | 0 | 5 | 2 | 1 | 321 | 1.00 | 1 |
| Windle 1995m | 1995 | 1.17 | 0.37 | PA | 0 | 5 | 2 | 1 | 481 | 0.00 | 1 |
| Windle 1995m | 1995 | 1.58 | 0.42 | SA | 0 | 5 | 2 | 1 | 481 | 0.00 | 1 |
| Wingenfeld 2013 | 2013 | 0.71 | 0.61 | EA | 1 | 3 | 1 | 2 | 38 | 0.82 | 0 |
| Wingenfeld 2013 | 2013 | 1.84 | 0.64 | EN | 1 | 3 | 1 | 2 | 38 | 0.82 | 0 |
| Wingenfeld 2013 | 2013 | 0.62 | 0.61 | PA | 1 | 3 | 1 | 2 | 38 | 0.82 | 0 |
| Wingenfeld 2013 | 2013 | 0.35 | 0.61 | PN | 1 | 3 | 1 | 2 | 38 | 0.82 | 0 |
| Wingenfeld 2013 | 2013 | -0.16 | 0.61 | SA | 1 | 3 | 1 | 2 | 38 | 0.82 | 0 |
| Wingenfeld 2017 | 2017 | 1.15 | 0.32 | EA | 1 | 3 | 1 | 2 | 137 | 1.00 | 0 |
| Wingenfeld 2017 | 2017 | 1.25 | 0.32 | EN | 1 | 3 | 1 | 2 | 137 | 1.00 | 0 |
| Wingenfeld 2017 | 2017 | -0.67 | 0.31 | PA | 1 | 3 | 1 | 2 | 137 | 1.00 | 0 |
| Wingenfeld 2017 | 2017 | 0.57 | 0.31 | PN | 1 | 3 | 1 | 2 | 137 | 1.00 | 0 |
| Wingenfeld 2017 | 2017 | 0.06 | 0.31 | SA | 1 | 3 | 1 | 2 | 137 | 1.00 | 0 |
| Wise 2001 | 2001 | 1.47 | 0.19 | PA | 0 | 5 | 4 | 1 | 732 | 1.00 | 1 |
| Wise 2001 | 2001 | 1.51 | 0.23 | SA | 0 | 5 | 4 | 1 | 732 | 1.00 | 1 |
| Xiao 2022 | 2022 | 1.36 | 0.18 | EA | 0 | 6 | 3 | 3 | 1134 | 0.74 | 0 |
| Xiao 2022 | 2022 | 0.41 | 0.18 | PA | 0 | 6 | 3 | 3 | 1134 | 0.74 | 0 |
| Xiao 2022 | 2022 | 0.71 | 0.17 | PN | 0 | 6 | 3 | 3 | 1134 | 0.74 | 0 |
| Xiong 2020 | 2020 | 0.51 | 0.12 | EA | 1 | 3 | 2 | 3 | 1065 | 0.64 | 0 |
| Xiong 2020 | 2020 | 0.66 | 0.12 | EN | 1 | 3 | 2 | 3 | 1065 | 0.64 | 0 |
| Xiong 2020 | 2020 | 0.29 | 0.11 | PA | 1 | 3 | 2 | 3 | 1065 | 0.64 | 0 |
| Xiong 2020 | 2020 | 0.86 | 0.12 | PN | 1 | 3 | 2 | 3 | 1065 | 0.64 | 0 |
| Xiong 2020 | 2020 | 0.48 | 0.11 | SA | 1 | 3 | 2 | 3 | 1065 | 0.64 | 0 |
| Yang 2017 | 2017 | 1.39 | 0.29 | EA | 1 | 3 | 1 | 3 | 168 | 0.73 | 0 |
| Yang 2017 | 2017 | 1.78 | 0.30 | EN | 1 | 3 | 1 | 3 | 168 | 0.73 | 0 |
| Yang 2017 | 2017 | 0.67 | 0.28 | PA | 1 | 3 | 1 | 3 | 168 | 0.73 | 0 |
| Yang 2017 | 2017 | 1.22 | 0.29 | PN | 1 | 3 | 1 | 3 | 168 | 0.73 | 0 |
| Yang 2017 | 2017 | 0.44 | 0.28 | SA | 1 | 3 | 1 | 3 | 168 | 0.73 | 0 |
| Yen 2008 | 2008 | 0.38 | 0.21 | PA | 0 | 6 | 4 | 3 | 1684 | 0.51 | 0 |
| Yin 2020 | 2020 | 2.15 | 0.31 | EA | 1 | 3 | 2 | 3 | 362 | 0.59 | 0 |
| Yin 2020 | 2020 | 1.83 | 0.28 | EN | 1 | 3 | 2 | 3 | 362 | 0.59 | 0 |
| Yin 2020 | 2020 | 1.27 | 0.22 | PA | 1 | 3 | 2 | 3 | 362 | 0.59 | 0 |
| Yin 2020 | 2020 | 1.40 | 0.24 | PN | 1 | 3 | 2 | 3 | 362 | 0.59 | 0 |
| Yin 2020 | 2020 | 0.98 | 0.21 | SA | 1 | 3 | 2 | 3 | 362 | 0.59 | 0 |
| Young 1997 | 1997 | 0.17 | 0.23 | EA | 1 | 6 | 2 | 1 | 650 | 0.67 | 1 |
| Young 1997 | 1997 | 0.31 | 0.27 | PA | 1 | 6 | 2 | 1 | 650 | 0.67 | 1 |
| Young 1997 | 1997 | 0.34 | 0.34 | SA | 1 | 6 | 2 | 1 | 650 | 0.67 | 1 |
| Yu 2022 | 2022 | 2.21 | 0.16 | EA | 0 | 6 | 3 | 3 | 687 | 0.64 | 0 |
| Zavaschi 2006 | 2006 | 0.89 | 0.37 | PA | 0 | 6 | 1 | 4 | 140 | 0.93 | 0 |
| Zavaschi 2006 | 2006 | 1.18 | 0.49 | SA | 0 | 6 | 1 | 4 | 140 | 0.93 | 0 |
| Zelikovsky 2002 | 2008 | 0.99 | 0.34 | EA | 0 | 6 | 3 | 1 | 100 | 0.51 | 1 |
| Zelikovsky 2002 | 2008 | 1.35 | 0.50 | PA | 0 | 6 | 3 | 1 | 100 | 0.51 | 1 |
| Zhao 2018 | 2018 | 0.94 | 0.72 | EA | 1 | 3 | 2 | 3 | 40 | 0.00 | 0 |
| Zhao 2018 | 2018 | 1.06 | 0.73 | EN | 1 | 3 | 2 | 3 | 40 | 0.00 | 0 |
| Zhao 2018 | 2018 | 0.86 | 0.70 | PA | 1 | 3 | 2 | 3 | 40 | 0.00 | 0 |
| Zhao 2018 | 2018 | 1.44 | 0.84 | PN | 1 | 3 | 2 | 3 | 40 | 0.00 | 0 |
| Zhao 2018 | 2018 | 0.48 | 0.65 | SA | 1 | 3 | 2 | 3 | 40 | 0.00 | 0 |
| Zheng 2016 | 2016 | 0.83 | 0.09 | EN | 0 | 5 | 3 | 3 | 9198 | . | 0 |
| Zuravin 1999 | 1999 | 1.46 | 0.24 | SA | 0 | 6 | 3 | 1 | 513 | 1.00 | 1 |

**Severity data**

| **study** | **year** | **zr** | **se(zr)** | **CM** | **complete abuse** | **CM meas code** | **sample type code** | **country code** | **effective N** | **% female** | **English-speaking** |
| --- | --- | --- | --- | --- | --- | --- | --- | --- | --- | --- | --- |
| Aguilera 2009 | 2009 | 0.33 | 0.04 | EA | 1 | 1 | 3 | 2 | 521 | 0.55 | 0 |
| Aguilera 2009 | 2009 | 0.28 | 0.04 | EN | 1 | 1 | 3 | 2 | 521 | 0.55 | 0 |
| Aguilera 2009 | 2009 | 0.13 | 0.04 | PA | 1 | 1 | 3 | 2 | 521 | 0.55 | 0 |
| Aguilera 2009 | 2009 | 0.12 | 0.04 | PN | 1 | 1 | 3 | 2 | 521 | 0.55 | 0 |
| Aguilera 2009 | 2009 | 0.22 | 0.04 | SA | 1 | 1 | 3 | 2 | 521 | 0.55 | 0 |
| Akbaba Turkoglu 2015 | 2015 | 0.37 | 0.12 | EA | 1 | 1 | 2 | 5 | 120 | 1.00 | 0 |
| Akbaba Turkoglu 2015 | 2015 | 0.31 | 0.12 | EN | 1 | 1 | 2 | 5 | 120 | 1.00 | 0 |
| Akbaba Turkoglu 2015 | 2015 | 0.16 | 0.09 | PA | 1 | 1 | 2 | 5 | 120 | 1.00 | 0 |
| Akbaba Turkoglu 2015 | 2015 | 0.22 | 0.12 | PN | 1 | 1 | 2 | 5 | 120 | 1.00 | 0 |
| Akbaba Turkoglu 2015 | 2015 | 0.16 | 0.12 | SA | 1 | 1 | 2 | 5 | 120 | 1.00 | 0 |
| Allen 1998 | 1998 | 0.15 | 0.08 | EA | 1 | 1 | 2 | 1 | 142 | 1.00 | 1 |
| Allen 1998 | 1998 | 0.27 | 0.08 | EN | 1 | 1 | 2 | 1 | 142 | 1.00 | 1 |
| Allen 1998 | 1998 | 0.11 | 0.08 | PA | 1 | 1 | 2 | 1 | 142 | 1.00 | 1 |
| Allen 1998 | 1998 | 0.09 | 0.08 | PN | 1 | 1 | 2 | 1 | 142 | 1.00 | 1 |
| Allen 1998 | 1998 | 0.16 | 0.08 | SA | 1 | 1 | 2 | 1 | 142 | 1.00 | 1 |
| Allen 2008 | 2008 | 0.22 | 0.07 | PA | 0 | 0 | 3 | 1 | 230 | 0.59 | 1 |
| Arata 2005 | 2005 | 0.41 | 0.05 | EA | 1 | 1 | 3 | 1 | 383 | 0.70 | 1 |
| Arata 2005 | 2005 | 0.35 | 0.05 | EN | 1 | 1 | 3 | 1 | 383 | 0.70 | 1 |
| Arata 2005 | 2005 | 0.30 | 0.05 | PA | 1 | 1 | 3 | 1 | 383 | 0.70 | 1 |
| Arata 2005 | 2005 | 0.32 | 0.05 | PN | 1 | 1 | 3 | 1 | 383 | 0.70 | 1 |
| Arata 2005 | 2005 | 0.24 | 0.05 | SA | 1 | 1 | 3 | 1 | 383 | 0.70 | 1 |
| Arslan 2015 | 2015 | 0.40 | 0.06 | EA | 0 | 1 | 3 | 5 | 320 | 0.66 | 0 |
| Auslander 2016 | 2016 | 0.41 | 0.07 | EA | 1 | 1 | 3 | 1 | 237 | 1.00 | 1 |
| Auslander 2016 | 2016 | 0.15 | 0.07 | PA | 1 | 1 | 3 | 1 | 237 | 1.00 | 1 |
| Auslander 2016 | 2016 | 0.26 | 0.07 | SA | 1 | 1 | 3 | 1 | 237 | 1.00 | 1 |
| Bagley 1994 | 1994 | 0.30 | 0.04 | EA | 0 | 0 | 4 | 1 | 750 | 0.00 | 1 |
| Bagley 1994 | 1994 | 0.37 | 0.04 | SA | 0 | 0 | 4 | 1 | 750 | 0.00 | 1 |
| Bailer 2014 | 2014 | 0.48 | 0.08 | EA | 1 | 1 | 2 | 2 | 162 | 0.59 | 0 |
| Bailer 2014 | 2014 | 0.40 | 0.08 | EN | 1 | 1 | 2 | 2 | 162 | 0.59 | 0 |
| Bailer 2014 | 2014 | 0.24 | 0.08 | PA | 1 | 1 | 2 | 2 | 162 | 0.59 | 0 |
| Bailer 2014 | 2014 | 0.30 | 0.08 | PN | 1 | 1 | 2 | 2 | 162 | 0.59 | 0 |
| Bailer 2014 | 2014 | 0.12 | 0.08 | SA | 1 | 1 | 2 | 2 | 162 | 0.59 | 0 |
| Balsam 2010 | 2010 | 0.34 | 0.04 | EA | 1 | 1 | 3 | 1 | 669 | 0.62 | 1 |
| Balsam 2010 | 2010 | 0.24 | 0.04 | PA | 1 | 1 | 3 | 1 | 669 | 0.62 | 1 |
| Balsam 2010 | 2010 | 0.08 | 0.04 | SA | 1 | 1 | 3 | 1 | 669 | 0.62 | 1 |
| Banducci 2014 | 2014 | 0.47 | 0.07 | EA | 0 | 1 | 2 | 1 | 280 | 0.30 | 1 |
| Basu 2013 | 2013 | 0.30 | 0.11 | PA | 0 | 1 | 3 | 1 | 88 | 1.00 | 1 |
| Basu 2013 | 2013 | 0.27 | 0.11 | SA | 0 | 1 | 3 | 1 | 88 | 1.00 | 1 |
| Berenbaum 2003 | 2003 | 0.20 | 0.12 | EA | 1 | 1 | 3 | 1 | 75 | 1.00 | 1 |
| Berenbaum 2003 | 2003 | 0.31 | 0.12 | EN | 1 | 1 | 3 | 1 | 75 | 1.00 | 1 |
| Berenbaum 2003 | 2003 | 0.26 | 0.12 | PA | 1 | 1 | 3 | 1 | 75 | 1.00 | 1 |
| Berenbaum 2003 | 2003 | 0.28 | 0.12 | PN | 1 | 1 | 3 | 1 | 75 | 1.00 | 1 |
| Berenbaum 2003 | 2003 | 0.12 | 0.12 | SA | 1 | 1 | 3 | 1 | 75 | 1.00 | 1 |
| Blain 2012 | 2012 | 0.35 | 0.07 | EA | 1 | 1 | 3 | 1 | 182 | 0.00 | 1 |
| Blain 2012 | 2012 | 0.38 | 0.07 | PA | 1 | 1 | 3 | 1 | 182 | 0.00 | 1 |
| Blain 2012 | 2012 | 0.37 | 0.07 | SA | 1 | 1 | 3 | 1 | 182 | 0.00 | 1 |
| Bohn 2013 | 2013 | 0.04 | 0.09 | EA | 1 | 1 | 3 | 2 | 117 | 0.84 | 0 |
| Bohn 2013 | 2013 | 0.04 | 0.09 | EN | 1 | 1 | 3 | 2 | 117 | 0.84 | 0 |
| Bohn 2013 | 2013 | 0.00 | 0.09 | PA | 1 | 1 | 3 | 2 | 117 | 0.84 | 0 |
| Bohn 2013 | 2013 | 0.10 | 0.09 | PN | 1 | 1 | 3 | 2 | 117 | 0.84 | 0 |
| Bohn 2013 | 2013 | 0.08 | 0.09 | SA | 1 | 1 | 3 | 2 | 117 | 0.84 | 0 |
| Brodsky 1995 | 1995 | 0.33 | 0.13 | SA | 0 | 0 | 3 | 1 | 60 | 1.00 | 1 |
| Brown 2016 | 2016 | 0.31 | 0.05 | EA | 1 | 1 | 3 | 1 | 339 | 0.49 | 1 |
| Brown 2016 | 2016 | 0.21 | 0.05 | EN | 1 | 1 | 3 | 1 | 339 | 0.49 | 1 |
| Brown 2016 | 2016 | 0.15 | 0.05 | PA | 1 | 1 | 3 | 1 | 339 | 0.49 | 1 |
| Brown 2016 | 2016 | 0.11 | 0.05 | PN | 1 | 1 | 3 | 1 | 339 | 0.49 | 1 |
| Brown 2016 | 2016 | 0.18 | 0.05 | SA | 1 | 1 | 3 | 1 | 339 | 0.49 | 1 |
| Burns 2012 | 2012 | 0.35 | 0.03 | EA | 0 | 1 | 3 | 1 | 996 | 1.00 | 1 |
| Burns 2012 | 2012 | 0.35 | 0.03 | EN | 0 | 1 | 3 | 1 | 996 | 1.00 | 1 |
| Caldwell 2011 | 2011 | 0.54 | 0.12 | EA | 1 | 1 | 3 | 1 | 76 | 1.00 | 1 |
| Caldwell 2011 | 2011 | 0.46 | 0.12 | EN | 1 | 1 | 3 | 1 | 76 | 1.00 | 1 |
| Caldwell 2011 | 2011 | 0.35 | 0.12 | PA | 1 | 1 | 3 | 1 | 76 | 1.00 | 1 |
| Caldwell 2011 | 2011 | 0.44 | 0.12 | PN | 1 | 1 | 3 | 1 | 76 | 1.00 | 1 |
| Caldwell 2011 | 2011 | 0.21 | 0.12 | SA | 1 | 1 | 3 | 1 | 76 | 1.00 | 1 |
| Calvete 2014 | 2014 | 0.29 | 0.03 | EA | 0 | 1 | 3 | 2 | 1052 | 0.47 | 0 |
| Cao 2020 | 2020 | 0.35 | 0.08 | EA | 1 | 1 | 2 | 3 | 239 | 0.00 | 0 |
| Cao 2020 | 2020 | 0.22 | 0.07 | EN | 1 | 1 | 2 | 3 | 239 | 0.00 | 0 |
| Cao 2020 | 2020 | 0.09 | 0.07 | PA | 1 | 1 | 2 | 3 | 239 | 0.00 | 0 |
| Cao 2020 | 2020 | 0.19 | 0.07 | PN | 1 | 1 | 2 | 3 | 239 | 0.00 | 0 |
| Cao 2020 | 2020 | 0.03 | 0.07 | SA | 1 | 1 | 2 | 3 | 239 | 0.00 | 0 |
| Carlson 2001 | 2001 | 0.20 | 0.08 | PA | 0 | 0 | 3 | 1 | 178 | 0.52 | 1 |
| Carlson 2001 | 2001 | 0.37 | 0.08 | SA | 0 | 0 | 3 | 1 | 178 | 0.52 | 1 |
| Chen 2017 | 2017 | 0.20 | 0.02 | EA | 1 | 1 | 3 | 3 | 1705 | 0.38 | 0 |
| Chen 2017 | 2017 | 0.09 | 0.02 | EN | 1 | 1 | 3 | 3 | 1705 | 0.38 | 0 |
| Chen 2017 | 2017 | 0.19 | 0.02 | PA | 1 | 1 | 3 | 3 | 1705 | 0.38 | 0 |
| Chen 2017 | 2017 | 0.16 | 0.02 | PN | 1 | 1 | 3 | 3 | 1705 | 0.38 | 0 |
| Chen 2017 | 2017 | 0.12 | 0.02 | SA | 1 | 1 | 3 | 3 | 1705 | 0.38 | 0 |
| Choi 2015 | 2015 | 0.34 | 0.11 | EA | 1 | 1 | 2 | 2 | 84 | 1.00 | 0 |
| Choi 2015 | 2015 | 0.00 | 0.11 | EN | 1 | 1 | 2 | 2 | 84 | 1.00 | 0 |
| Choi 2015 | 2015 | 0.21 | 0.11 | PA | 1 | 1 | 2 | 2 | 84 | 1.00 | 0 |
| Choi 2015 | 2015 | -0.01 | 0.11 | PN | 1 | 1 | 2 | 2 | 84 | 1.00 | 0 |
| Choi 2015 | 2015 | 0.26 | 0.11 | SA | 1 | 1 | 2 | 2 | 84 | 1.00 | 0 |
| Coates 2014 | 2014 | 0.39 | 0.04 | EA | 0 | 0 | 3 | 1 | 771 | 1.00 | 1 |
| Coates 2014 | 2014 | 0.30 | 0.04 | EN | 0 | 0 | 3 | 1 | 771 | 1.00 | 1 |
| Cohen 2017 | 2017 | 0.47 | 0.04 | EA | 1 | 1 | 3 | 1 | 580 | 0.58 | 1 |
| Cohen 2017 | 2017 | 0.40 | 0.04 | EN | 1 | 1 | 3 | 1 | 580 | 0.58 | 1 |
| Cohen 2017 | 2017 | 0.23 | 0.04 | PA | 1 | 1 | 3 | 1 | 580 | 0.58 | 1 |
| Cohen 2017 | 2017 | 0.27 | 0.04 | PN | 1 | 1 | 3 | 1 | 580 | 0.58 | 1 |
| Cohen 2017 | 2017 | 0.17 | 0.04 | SA | 1 | 1 | 3 | 1 | 580 | 0.58 | 1 |
| Courtney 2008 | 2008 | 0.52 | 0.07 | EA | 0 | 1 | 3 | 1 | 195 | 0.79 | 1 |
| Courtney 2008 | 2008 | 0.40 | 0.07 | PA | 0 | 1 | 3 | 1 | 195 | 0.79 | 1 |
| Crow 2014 | 2014 | 0.48 | 0.02 | EA | 1 | 1 | 3 | 1 | 3902 | 0.69 | 1 |
| Crow 2014 | 2014 | 0.39 | 0.02 | EN | 1 | 1 | 3 | 1 | 3902 | 0.69 | 1 |
| Crow 2014 | 2014 | 0.34 | 0.02 | PA | 1 | 1 | 3 | 1 | 3902 | 0.69 | 1 |
| Crow 2014 | 2014 | 0.30 | 0.02 | PN | 1 | 1 | 3 | 1 | 3902 | 0.69 | 1 |
| Crow 2014 | 2014 | 0.31 | 0.02 | SA | 1 | 1 | 3 | 1 | 3902 | 0.69 | 1 |
| Cui 2011 | 2011 | 0.31 | 0.05 | EA | 1 | 1 | 3 | 3 | 668 | 0.54 | 0 |
| Cui 2011 | 2011 | 0.38 | 0.05 | EN | 1 | 1 | 3 | 3 | 668 | 0.54 | 0 |
| Cui 2011 | 2011 | 0.14 | 0.04 | PA | 1 | 1 | 3 | 3 | 668 | 0.54 | 0 |
| Cui 2011 | 2011 | 0.30 | 0.04 | PN | 1 | 1 | 3 | 3 | 668 | 0.54 | 0 |
| Cui 2011 | 2011 | 0.11 | 0.04 | SA | 1 | 1 | 3 | 3 | 668 | 0.54 | 0 |
| Dackis 2012 | 2012 | 0.39 | 0.07 | EA | 1 | 1 | 3 | 1 | 236 | 1.00 | 1 |
| Dackis 2012 | 2012 | 0.23 | 0.07 | EN | 1 | 1 | 3 | 1 | 236 | 1.00 | 1 |
| Dackis 2012 | 2012 | 0.35 | 0.07 | PA | 1 | 1 | 3 | 1 | 236 | 1.00 | 1 |
| Dackis 2012 | 2012 | 0.28 | 0.07 | PN | 1 | 1 | 3 | 1 | 236 | 1.00 | 1 |
| Dackis 2012 | 2012 | 0.39 | 0.07 | SA | 1 | 1 | 3 | 1 | 236 | 1.00 | 1 |
| Day 2013 | 2013 | 0.50 | 0.10 | EA | 0 | 1 | 3 | 1 | 112 | 0.39 | 1 |
| Day 2013 | 2013 | 0.30 | 0.10 | PA | 0 | 1 | 3 | 1 | 112 | 0.39 | 1 |
| Dunkley 2010 | 2010 | 0.20 | 0.08 | EA | 1 | 1 | 3 | 1 | 170 | 0.78 | 1 |
| Dunkley 2010 | 2010 | 0.09 | 0.08 | EN | 1 | 1 | 3 | 1 | 170 | 0.78 | 1 |
| Dunkley 2010 | 2010 | 0.08 | 0.08 | PA | 1 | 1 | 3 | 1 | 170 | 0.78 | 1 |
| Dunkley 2010 | 2010 | 0.03 | 0.08 | PN | 1 | 1 | 3 | 1 | 170 | 0.78 | 1 |
| Dunkley 2010 | 2010 | 0.09 | 0.08 | SA | 1 | 1 | 3 | 1 | 170 | 0.78 | 1 |
| Dunlop 2015f | 2015 | 0.17 | 0.07 | SA | 0 | 1 | 2 | 1 | 191 | 1.00 | 1 |
| Dunlop 2015m | 2015 | 0.15 | 0.09 | SA | 0 | 1 | 2 | 1 | 140 | 0.00 | 1 |
| Ensink 2016 | 2016 | 0.39 | 0.08 | SA | 0 | 0 | 1 | 1 | 168 | 0.57 | 1 |
| Gerke 2006 | 2006 | 0.30 | 0.05 | EA | 1 | 1 | 3 | 1 | 417 | 1.00 | 1 |
| Gerke 2006 | 2006 | 0.26 | 0.05 | EN | 1 | 1 | 3 | 1 | 417 | 1.00 | 1 |
| Gerke 2006 | 2006 | 0.11 | 0.05 | PA | 1 | 1 | 3 | 1 | 417 | 1.00 | 1 |
| Gerke 2006 | 2006 | 0.21 | 0.05 | PN | 1 | 1 | 3 | 1 | 417 | 1.00 | 1 |
| Gerke 2006 | 2006 | 0.01 | 0.05 | SA | 1 | 1 | 3 | 1 | 417 | 1.00 | 1 |
| Gibb 2003 | 2003 | 0.35 | 0.08 | EA | 0 | 0 | 3 | 1 | 220 | 0.75 | 1 |
| Gibb 2007 | 2007 | 0.38 | 0.07 | EA | 0 | 0 | 3 | 1 | 212 | 0.74 | 1 |
| Gibb 2008 | 2008 | 0.59 | 0.10 | EA | 0 | 1 | 3 | 1 | 105 | 0.51 | 0 |
| Goldsmith 2005 | 2005 | 0.54 | 0.11 | EA | 0 | 0 | 3 | 1 | 80 | 0.63 | 1 |
| Goldsmith 2005 | 2005 | 0.71 | 0.11 | EN | 0 | 0 | 3 | 1 | 80 | 0.63 | 1 |
| Goldstein 2012 | 2012 | 0.37 | 0.11 | EA | 0 | 1 | 3 | 1 | 202 | 0.54 | 1 |
| Goldstein 2013 | 2013 | 0.68 | 0.07 | EA | 1 | 1 | 3 | 1 | 93 | 0.76 | 1 |
| Goldstein 2013 | 2013 | 0.29 | 0.11 | EN | 1 | 1 | 3 | 1 | 93 | 0.76 | 1 |
| Goldstein 2013 | 2013 | 0.20 | 0.11 | PA | 1 | 1 | 3 | 1 | 93 | 0.76 | 1 |
| Goldstein 2013 | 2013 | 0.24 | 0.11 | SA | 1 | 1 | 3 | 1 | 93 | 0.76 | 1 |
| Gong & Chan 2018 | 2018 | 0.27 | 0.03 | EA | 1 | 1 | 3 | 3 | 1102 | 0.73 | 0 |
| Gong & Chan 2018 | 2018 | 0.27 | 0.03 | EN | 1 | 1 | 3 | 3 | 1102 | 0.73 | 0 |
| Gong & Chan 2018 | 2018 | 0.06 | 0.03 | PA | 1 | 1 | 3 | 3 | 1102 | 0.73 | 0 |
| Gong & Chan 2018 | 2018 | 0.22 | 0.03 | PN | 1 | 1 | 3 | 3 | 1102 | 0.73 | 0 |
| Gong & Chan 2018 | 2018 | 0.05 | 0.03 | SA | 1 | 1 | 3 | 3 | 1102 | 0.73 | 0 |
| Gong 2016 | 2016 | 0.22 | 0.08 | EA | 1 | 1 | 1 | 3 | 183 | 0.77 | 0 |
| Gong 2016 | 2016 | 0.21 | 0.08 | EN | 1 | 1 | 1 | 3 | 183 | 0.77 | 0 |
| Gong 2016 | 2016 | -0.07 | 0.08 | PA | 1 | 1 | 1 | 3 | 183 | 0.77 | 0 |
| Gong 2016 | 2016 | 0.12 | 0.08 | PN | 1 | 1 | 1 | 3 | 183 | 0.77 | 0 |
| Gong 2016 | 2016 | 0.04 | 0.08 | SA | 1 | 1 | 1 | 3 | 183 | 0.77 | 0 |
| Gratz 2011 | 2011 | 0.60 | 0.05 | EA | 0 | 1 | 3 | 1 | 225 | 0.45 | 1 |
| Groleau 2012 | 2012 | 0.16 | 0.06 | EA | 1 | 0 | 1 | 1 | 315 | 1.00 | 1 |
| Groleau 2012 | 2012 | 0.22 | 0.06 | PA | 1 | 0 | 1 | 1 | 315 | 1.00 | 1 |
| Groleau 2012 | 2012 | 0.09 | 0.06 | SA | 1 | 0 | 1 | 1 | 315 | 1.00 | 1 |
| Haapasalo 1999 | 1999 | 0.03 | 0.07 | EA | 0 | 0 | 3 | 2 | 89 | 0.00 | 0 |
| Haj-Yahia 2008 | 2008 | 0.30 | 0.05 | PA | 0 | 0 | 3 | 5 | 476 | 0.51 | 0 |
| Hamilton 2016 | 2016 | 0.46 | 0.08 | EA | 0 | 1 | 3 | 1 | 410 | 0.53 | 1 |
| Hamilton 2016 | 2016 | 0.48 | 0.05 | EN | 0 | 1 | 3 | 1 | 410 | 0.53 | 1 |
| Hankin 2005 sample1 | 2005 | 0.26 | 0.04 | EA | 1 | 0 | 3 | 1 | 652 | 0.68 | 1 |
| Hankin 2005 sample1 | 2005 | 0.09 | 0.04 | PA | 1 | 0 | 3 | 1 | 652 | 0.68 | 1 |
| Hankin 2005 sample1 | 2005 | 0.15 | 0.04 | SA | 1 | 0 | 3 | 1 | 652 | 0.68 | 1 |
| Hankin 2005 sample2 | 2005 | 0.13 | 0.12 | EA | 1 | 0 | 3 | 1 | 75 | 0.55 | 1 |
| Hankin 2005 sample2 | 2005 | 0.13 | 0.12 | EN | 1 | 0 | 3 | 1 | 75 | 0.55 | 1 |
| Hankin 2005 sample2 | 2005 | 0.04 | 0.12 | PA | 1 | 0 | 3 | 1 | 75 | 0.55 | 1 |
| Hankin 2005 sample2 | 2005 | 0.03 | 0.12 | SA | 1 | 0 | 3 | 1 | 75 | 0.55 | 1 |
| Harding 2012 | 2012 | 0.33 | 0.08 | EN | 0 | 1 | 3 | 1 | 157 | 1.00 | 1 |
| Harding 2012 | 2012 | 0.22 | 0.08 | PA | 0 | 1 | 3 | 1 | 157 | 1.00 | 1 |
| Harding 2012 | 2012 | 0.17 | 0.08 | SA | 0 | 1 | 3 | 1 | 157 | 1.00 | 1 |
| Heckman 2006 | 2006 | 0.30 | 0.09 | EN | 0 | 0 | 3 | 1 | 138 | 0.84 | 1 |
| Heckman 2006 | 2006 | 0.30 | 0.09 | PN | 0 | 0 | 3 | 1 | 138 | 0.84 | 1 |
| Heckman 2006 | 2006 | 0.06 | 0.09 | SA | 0 | 0 | 3 | 1 | 138 | 0.84 | 1 |
| Hobfoll 2002 | 2002 | 0.34 | 0.08 | PA | 0 | 1 | 3 | 1 | 160 | 1.00 | 1 |
| Hobfoll 2002 | 2002 | 0.05 | 0.08 | SA | 0 | 1 | 3 | 1 | 160 | 1.00 | 1 |
| Hopwood 2011f | 2011 | 0.54 | 0.11 | EA | 1 | 1 | 2 | 1 | 82 | 1.00 | 1 |
| Hopwood 2011f | 2011 | 0.41 | 0.11 | PA | 1 | 1 | 2 | 1 | 82 | 1.00 | 1 |
| Hopwood 2011f | 2011 | 0.42 | 0.11 | SA | 1 | 1 | 2 | 1 | 82 | 1.00 | 1 |
| Hopwood 2011m | 2011 | 0.47 | 0.13 | EA | 1 | 1 | 2 | 1 | 66 | 0.00 | 1 |
| Hopwood 2011m | 2011 | 0.11 | 0.13 | PA | 1 | 1 | 2 | 1 | 66 | 0.00 | 1 |
| Hopwood 2011m | 2011 | 0.01 | 0.13 | SA | 1 | 1 | 2 | 1 | 66 | 0.00 | 1 |
| Huang 2021 | 2021 | 0.04 | 0.03 | PA | 0 | 0 | 4 | 1 | 1690 | 1.00 | 1 |
| Hund 2005 | 2005 | 0.15 | 0.04 | SA | 0 | 1 | 3 | 1 | 608 | 1.00 | 1 |
| Hund 2006 | 2006 | 0.35 | 0.04 | EA | 0 | 1 | 3 | 1 | 608 | 1.00 | 1 |
| Inanici 2017 | 2017 | 0.61 | 0.08 | EA | 1 | 1 | 3 | 5 | 144 | 1.00 | 0 |
| Inanici 2017 | 2017 | 0.35 | 0.08 | EN | 1 | 1 | 3 | 5 | 144 | 1.00 | 0 |
| Inanici 2017 | 2017 | 0.29 | 0.08 | PA | 1 | 1 | 3 | 5 | 144 | 1.00 | 0 |
| Inanici 2017 | 2017 | 0.41 | 0.08 | PN | 1 | 1 | 3 | 5 | 144 | 1.00 | 0 |
| Inanici 2017 | 2017 | 0.37 | 0.08 | SA | 1 | 1 | 3 | 5 | 144 | 1.00 | 0 |
| Jager-Hyman 2012 | 2012 | 0.40 | 0.02 | EA | 1 | 0 | 3 | 1 | 1819 | 0.60 | 1 |
| Jager-Hyman 2012 | 2012 | 0.34 | 0.02 | PA | 1 | 0 | 3 | 1 | 1819 | 0.60 | 1 |
| Jager-Hyman 2012 | 2012 | 0.26 | 0.02 | SA | 1 | 0 | 3 | 1 | 1819 | 0.60 | 1 |
| James 2012 | 2012 | 0.27 | 0.06 | EA | 0 | 1 | 3 | 1 | 286 | 0.00 | 1 |
| Jessar 2017 | 2017 | 0.45 | 0.07 | EA | 0 | 1 | 3 | 1 | 204 | 0.54 | 1 |
| Jessar 2017 | 2017 | 0.30 | 0.07 | EN | 0 | 1 | 3 | 1 | 204 | 0.54 | 1 |
| Jin 2014 | 2014 | 0.42 | 0.09 | EA | 1 | 1 | 3 | 5 | 134 | 0.00 | 0 |
| Jin 2014 | 2014 | 0.29 | 0.09 | EN | 1 | 1 | 3 | 5 | 134 | 0.00 | 0 |
| Jin 2014 | 2014 | 0.54 | 0.09 | PA | 1 | 1 | 3 | 5 | 134 | 0.00 | 0 |
| Jin 2014 | 2014 | 0.41 | 0.09 | PN | 1 | 1 | 3 | 5 | 134 | 0.00 | 0 |
| Jin 2014 | 2014 | 0.45 | 0.09 | SA | 1 | 1 | 3 | 5 | 134 | 0.00 | 0 |
| Kang 2020 | 2020 | 0.50 | 0.04 | EA | 1 | 1 | 3 | 3 | 942 | 0.52 | 0 |
| Kang 2020 | 2020 | 0.22 | 0.03 | EN | 1 | 1 | 3 | 3 | 942 | 0.52 | 0 |
| Kang 2020 | 2020 | 0.26 | 0.03 | PA | 1 | 1 | 3 | 3 | 942 | 0.52 | 0 |
| Kang 2020 | 2020 | 0.15 | 0.03 | PN | 1 | 1 | 3 | 3 | 942 | 0.52 | 0 |
| Kang 2020 | 2020 | 0.20 | 0.03 | SA | 1 | 1 | 3 | 3 | 942 | 0.52 | 0 |
| Kecojevic 2015 | 2015 | 0.45 | 0.07 | EA | 1 | 1 | 3 | 1 | 191 | 0.00 | 1 |
| Kecojevic 2015 | 2015 | 0.40 | 0.07 | PA | 1 | 1 | 3 | 1 | 191 | 0.00 | 1 |
| Kecojevic 2015 | 2015 | 0.31 | 0.07 | SA | 1 | 1 | 3 | 1 | 191 | 0.00 | 1 |
| Kilimnik 2016 | 2016 | 0.32 | 0.07 | SA | 0 | 1 | 3 | 1 | 222 | 1.00 | 1 |
| Kim 2017 | 2017 | 0.46 | 0.07 | EA | 1 | 1 | 3 | 3 | 207 | 0.59 | 0 |
| Kim 2017 | 2017 | 0.47 | 0.07 | EN | 1 | 1 | 3 | 3 | 207 | 0.59 | 0 |
| Kim 2017 | 2017 | 0.27 | 0.07 | PA | 1 | 1 | 3 | 3 | 207 | 0.59 | 0 |
| Kim 2017 | 2017 | 0.17 | 0.07 | PN | 1 | 1 | 3 | 3 | 207 | 0.59 | 0 |
| Kim 2017 | 2017 | 0.10 | 0.07 | SA | 1 | 1 | 3 | 3 | 207 | 0.59 | 0 |
| Klein 2008 | 2008 | 0.15 | 0.06 | EA | 0 | 1 | 3 | 1 | 250 | 1.00 | 1 |
| Klein 2008 | 2008 | 0.17 | 0.06 | SA | 0 | 1 | 3 | 1 | 250 | 1.00 | 1 |
| Klein 2009 | 2009 | 0.08 | 0.04 | SA | 0 | 1 | 2 | 1 | 808 | 0.55 | 1 |
| Kohrt 2004 | 2004 | 0.17 | 0.10 | EA | 0 | 0 | 3 | 3 | 99 | 0.00 | 0 |
| Kohrt 2004 | 2004 | 0.22 | 0.10 | PA | 0 | 0 | 3 | 3 | 99 | 0.00 | 0 |
| Kong 2009 | 2009 | 0.43 | 0.12 | EA | 1 | 1 | 3 | 3 | 73 | 0.97 | 0 |
| Kong 2009 | 2009 | 0.40 | 0.12 | EN | 1 | 1 | 3 | 3 | 73 | 0.97 | 0 |
| Kong 2009 | 2009 | 0.26 | 0.12 | PA | 1 | 1 | 3 | 3 | 73 | 0.97 | 0 |
| Kong 2009 | 2009 | 0.31 | 0.12 | PN | 1 | 1 | 3 | 3 | 73 | 0.97 | 0 |
| Kong 2009 | 2009 | 0.12 | 0.12 | SA | 1 | 1 | 3 | 3 | 73 | 0.97 | 0 |
| Kraaij 2001 | 2001 | 0.32 | 0.07 | EA | 1 | 0 | 4 | 2 | 194 | 0.52 | 0 |
| Kraaij 2001 | 2001 | 0.05 | 0.07 | PA | 1 | 0 | 4 | 2 | 194 | 0.52 | 0 |
| Kraaij 2001 | 2001 | 0.36 | 0.07 | SA | 1 | 0 | 4 | 2 | 194 | 0.52 | 0 |
| Krastins 2014 | 2014 | 0.32 | 0.05 | EA | 1 | 1 | 3 | 1 | 411 | 0.76 | 1 |
| Krastins 2014 | 2014 | 0.34 | 0.05 | EN | 1 | 1 | 3 | 1 | 411 | 0.76 | 1 |
| Krastins 2014 | 2014 | 0.27 | 0.05 | PA | 1 | 1 | 3 | 1 | 411 | 0.76 | 1 |
| Krastins 2014 | 2014 | 0.26 | 0.05 | PN | 1 | 1 | 3 | 1 | 411 | 0.76 | 1 |
| Krastins 2014 | 2014 | 0.15 | 0.05 | SA | 1 | 1 | 3 | 1 | 411 | 0.76 | 1 |
| Kuo 2011 | 2011 | 0.18 | 0.09 | EA | 1 | 1 | 1 | 1 | 132 | 0.52 | 1 |
| Kuo 2011 | 2011 | 0.32 | 0.09 | EN | 1 | 1 | 1 | 1 | 132 | 0.52 | 1 |
| Kuo 2011 | 2011 | 0.11 | 0.09 | PA | 1 | 1 | 1 | 1 | 132 | 0.52 | 1 |
| Kuo 2011 | 2011 | 0.13 | 0.09 | PN | 1 | 1 | 1 | 1 | 132 | 0.52 | 1 |
| Kuo 2011 | 2011 | 0.07 | 0.09 | SA | 1 | 1 | 1 | 1 | 132 | 0.52 | 1 |
| Lang 2004 | 2004 | 0.10 | 0.12 | EA | 1 | 1 | 3 | 1 | 72 | 1.00 | 1 |
| Lang 2004 | 2004 | 0.52 | 0.12 | EN | 1 | 1 | 3 | 1 | 72 | 1.00 | 1 |
| Lang 2004 | 2004 | 0.23 | 0.12 | PA | 1 | 1 | 3 | 1 | 72 | 1.00 | 1 |
| Lang 2004 | 2004 | -0.03 | 0.12 | PN | 1 | 1 | 3 | 1 | 72 | 1.00 | 1 |
| Lang 2004 | 2004 | -0.14 | 0.12 | SA | 1 | 1 | 3 | 1 | 72 | 1.00 | 1 |
| Lang 2006 | 2006 | 0.42 | 0.16 | SA | 0 | 1 | 3 | 1 | 44 | 1.00 | 1 |
| Lang 2010 | 2010 | 0.45 | 0.16 | EA | 1 | 1 | 3 | 1 | 44 | 1.00 | 1 |
| Lang 2010 | 2010 | 0.50 | 0.16 | EN | 1 | 1 | 3 | 1 | 44 | 1.00 | 1 |
| Lang 2010 | 2010 | 0.44 | 0.16 | PA | 1 | 1 | 3 | 1 | 44 | 1.00 | 1 |
| Lang 2010 | 2010 | 0.45 | 0.16 | PN | 1 | 1 | 3 | 1 | 44 | 1.00 | 1 |
| Lang 2010 | 2010 | 0.48 | 0.16 | SA | 1 | 1 | 3 | 1 | 44 | 1.00 | 1 |
| Leenarts 2013 | 2013 | 0.39 | 0.08 | EA | 1 | 1 | 2 | 2 | 154 | 1.00 | 0 |
| Leenarts 2013 | 2013 | 0.27 | 0.08 | EN | 1 | 1 | 2 | 2 | 154 | 1.00 | 0 |
| Leenarts 2013 | 2013 | 0.27 | 0.08 | PA | 1 | 1 | 2 | 2 | 154 | 1.00 | 0 |
| Leenarts 2013 | 2013 | 0.32 | 0.08 | PN | 1 | 1 | 2 | 2 | 154 | 1.00 | 0 |
| Leenarts 2013 | 2013 | 0.26 | 0.08 | SA | 1 | 1 | 2 | 2 | 154 | 1.00 | 0 |
| Lewis 2006 | 2006 | 0.38 | 0.10 | EA | 1 | 1 | 3 | 1 | 102 | 1.00 | 1 |
| Lewis 2006 | 2006 | 0.26 | 0.10 | EN | 1 | 1 | 3 | 1 | 102 | 1.00 | 1 |
| Lewis 2006 | 2006 | -0.22 | 0.10 | PA | 1 | 1 | 3 | 1 | 102 | 1.00 | 1 |
| Lewis 2006 | 2006 | 0.07 | 0.10 | PN | 1 | 1 | 3 | 1 | 102 | 1.00 | 1 |
| Lewis 2006 | 2006 | 0.00 | 0.10 | SA | 1 | 1 | 3 | 1 | 102 | 1.00 | 1 |
| Li 2020 | 2020 | 0.45 | 0.04 | EN | 0 | 1 | 3 | 3 | 961 | 0.51 | 0 |
| Li 2020 | 2020 | 0.31 | 0.03 | PA | 0 | 1 | 3 | 3 | 961 | 0.51 | 0 |
| Li 2020 | 2020 | 0.39 | 0.04 | PN | 0 | 1 | 3 | 3 | 961 | 0.51 | 0 |
| Li 2020 | 2020 | 0.18 | 0.03 | SA | 0 | 1 | 3 | 3 | 961 | 0.51 | 0 |
| Liu 2012 | 2012 | 0.07 | 0.06 | EA | 1 | 0 | 3 | 1 | 299 | 0.68 | 1 |
| Liu 2012 | 2012 | 0.06 | 0.06 | PA | 1 | 0 | 3 | 1 | 299 | 0.68 | 1 |
| Liu 2012 | 2012 | 0.01 | 0.06 | SA | 1 | 0 | 3 | 1 | 299 | 0.68 | 1 |
| Liu 2013 | 2013 | 0.21 | 0.13 | EA | 1 | 1 | 3 | 1 | 66 | 0.77 | 1 |
| Liu 2013 | 2013 | 0.02 | 0.13 | PA | 1 | 1 | 3 | 1 | 66 | 0.77 | 1 |
| Liu 2013 | 2013 | 0.06 | 0.13 | SA | 1 | 1 | 3 | 1 | 66 | 0.77 | 1 |
| Liu 2017 | 2017 | 0.35 | 0.02 | EA | 1 | 1 | 3 | 3 | 2469 | 0.72 | 0 |
| Liu 2017 | 2017 | 0.31 | 0.02 | EN | 1 | 1 | 3 | 3 | 2469 | 0.72 | 0 |
| Liu 2017 | 2017 | 0.27 | 0.02 | PA | 1 | 1 | 3 | 3 | 2469 | 0.72 | 0 |
| Liu 2017 | 2017 | 0.33 | 0.02 | PN | 1 | 1 | 3 | 3 | 2469 | 0.72 | 0 |
| Liu 2017 | 2017 | 0.30 | 0.02 | SA | 1 | 1 | 3 | 3 | 2469 | 0.72 | 0 |
| Locke 2007 | 2007 | 0.18 | 0.03 | EA | 1 | 1 | 3 | 1 | 904 | 1.00 | 1 |
| Locke 2007 | 2007 | 0.37 | 0.03 | EN | 1 | 1 | 3 | 1 | 904 | 1.00 | 1 |
| Locke 2007 | 2007 | 0.15 | 0.03 | PA | 1 | 1 | 3 | 1 | 904 | 1.00 | 1 |
| Locke 2007 | 2007 | 0.32 | 0.03 | PN | 1 | 1 | 3 | 1 | 904 | 1.00 | 1 |
| Locke 2007 | 2007 | 0.14 | 0.03 | SA | 1 | 1 | 3 | 1 | 904 | 1.00 | 1 |
| Lopez 2011 | 2011 | 0.30 | 0.04 | EA | 1 | 1 | 3 | 1 | 813 | 1.00 | 1 |
| Lopez 2011 | 2011 | 0.14 | 0.04 | EN | 1 | 1 | 3 | 1 | 813 | 1.00 | 1 |
| Lopez 2011 | 2011 | 0.19 | 0.04 | PA | 1 | 1 | 3 | 1 | 813 | 1.00 | 1 |
| Lopez 2011 | 2011 | 0.19 | 0.04 | PN | 1 | 1 | 3 | 1 | 813 | 1.00 | 1 |
| Lopez 2011 | 2011 | 0.19 | 0.04 | SA | 1 | 1 | 3 | 1 | 813 | 1.00 | 1 |
| Lutenbacher 2000 | 2000 | 0.37 | 0.13 | PA | 0 | 0 | 3 | 1 | 109 | 1.00 | 1 |
| Lutenbacher 2000 | 2000 | 0.54 | 0.13 | SA | 0 | 0 | 3 | 1 | 109 | 1.00 | 1 |
| Luterek 2004 | 2004 | 0.12 | 0.05 | SA | 0 | 0 | 3 | 1 | 355 | 1.00 | 1 |
| MacDonald 2014 | 2014 | 0.17 | 0.07 | EA | 1 | 1 | 2 | 1 | 200 | 0.46 | 1 |
| MacDonald 2014 | 2014 | 0.13 | 0.07 | EN | 1 | 1 | 2 | 1 | 200 | 0.46 | 1 |
| MacDonald 2014 | 2014 | 0.04 | 0.07 | PA | 1 | 1 | 2 | 1 | 200 | 0.46 | 1 |
| MacDonald 2014 | 2014 | 0.12 | 0.07 | PN | 1 | 1 | 2 | 1 | 200 | 0.46 | 1 |
| MacDonald 2014 | 2014 | 0.15 | 0.07 | SA | 1 | 1 | 2 | 1 | 200 | 0.46 | 1 |
| Marquee-Flentje 2015 | 2015 | 0.52 | 0.06 | EA | 1 | 1 | 3 | 1 | 300 | 1.00 | 1 |
| Marquee-Flentje 2015 | 2015 | 0.45 | 0.06 | PA | 1 | 1 | 3 | 1 | 300 | 1.00 | 1 |
| Marquee-Flentje 2015 | 2015 | 0.27 | 0.06 | SA | 1 | 1 | 3 | 1 | 300 | 1.00 | 1 |
| Marse 2002 | 2002 | 0.20 | 0.06 | EA | 0 | 0 | 3 | 1 | 128 | 0.33 | 1 |
| Marse 2002 | 2002 | 0.29 | 0.09 | PA | 0 | 0 | 3 | 1 | 128 | 0.33 | 1 |
| Martsolf 2004 | 2004 | 0.44 | 0.06 | EA | 1 | 1 | 3 | 4 | 258 | 0.66 | 0 |
| Martsolf 2004 | 2004 | 0.19 | 0.06 | EN | 1 | 1 | 3 | 4 | 258 | 0.66 | 0 |
| Martsolf 2004 | 2004 | 0.39 | 0.06 | PA | 1 | 1 | 3 | 4 | 258 | 0.66 | 0 |
| Martsolf 2004 | 2004 | 0.18 | 0.06 | PN | 1 | 1 | 3 | 4 | 258 | 0.66 | 0 |
| Martsolf 2004 | 2004 | 0.28 | 0.06 | SA | 1 | 1 | 3 | 4 | 258 | 0.66 | 0 |
| Massing-Schaffer 2015 | 2015 | 0.31 | 0.07 | EA | 1 | 1 | 3 | 1 | 185 | 0.75 | 1 |
| Massing-Schaffer 2015 | 2015 | 0.14 | 0.07 | PA | 1 | 1 | 3 | 1 | 185 | 0.75 | 1 |
| Massing-Schaffer 2015 | 2015 | 0.08 | 0.07 | SA | 1 | 1 | 3 | 1 | 185 | 0.75 | 1 |
| Mazzeo 2008 (Aawomen) | 2008 | 0.28 | 0.07 | EA | 1 | 1 | 3 | 1 | 192 | 1.00 | 1 |
| Mazzeo 2008 (Aawomen) | 2008 | 0.21 | 0.07 | EN | 1 | 1 | 3 | 1 | 192 | 1.00 | 1 |
| Mazzeo 2008 (Aawomen) | 2008 | 0.18 | 0.07 | PA | 1 | 1 | 3 | 1 | 192 | 1.00 | 1 |
| Mazzeo 2008 (Aawomen) | 2008 | 0.22 | 0.07 | PN | 1 | 1 | 3 | 1 | 192 | 1.00 | 1 |
| Mazzeo 2008 (Aawomen) | 2008 | 0.02 | 0.07 | SA | 1 | 1 | 3 | 1 | 192 | 1.00 | 1 |
| Mazzeo 2008 (Eawomen) | 2008 | 0.20 | 0.05 | EA | 1 | 1 | 3 | 1 | 412 | 1.00 | 1 |
| Mazzeo 2008 (Eawomen) | 2008 | 0.16 | 0.05 | EN | 1 | 1 | 3 | 1 | 412 | 1.00 | 1 |
| Mazzeo 2008 (Eawomen) | 2008 | 0.05 | 0.05 | PA | 1 | 1 | 3 | 1 | 412 | 1.00 | 1 |
| Mazzeo 2008 (Eawomen) | 2008 | 0.15 | 0.05 | PN | 1 | 1 | 3 | 1 | 412 | 1.00 | 1 |
| Mazzeo 2008 (Eawomen) | 2008 | -0.05 | 0.05 | SA | 1 | 1 | 3 | 1 | 412 | 1.00 | 1 |
| McGinn 2005 | 2005 | 0.38 | 0.14 | EA | 1 | 1 | 2 | 1 | 55 | 0.89 | 1 |
| McGinn 2005 | 2005 | 0.51 | 0.14 | EN | 1 | 1 | 2 | 1 | 55 | 0.89 | 1 |
| McGinn 2005 | 2005 | 0.29 | 0.14 | PA | 1 | 1 | 2 | 1 | 55 | 0.89 | 1 |
| McGinn 2005 | 2005 | 0.41 | 0.14 | PN | 1 | 1 | 2 | 1 | 55 | 0.89 | 1 |
| McGinn 2005 | 2005 | 0.18 | 0.14 | SA | 1 | 1 | 2 | 1 | 55 | 0.89 | 1 |
| Mehta 2017 | 2017 | 0.23 | 0.07 | SA | 0 | 0 | 2 | 1 | 229 | 0.64 | 1 |
| Melmed 2012 | 2012 | 0.60 | 0.08 | EA | 0 | 0 | 3 | 1 | 169 | 0.00 | 1 |
| Melmed 2012 | 2012 | 0.20 | 0.08 | PA | 0 | 0 | 3 | 1 | 169 | 0.00 | 1 |
| Meyerson 2002 | 2002 | 0.23 | 0.09 | SA | 0 | 0 | 3 | 1 | 130 | 0.55 | 1 |
| Michopoulos 2015 | 2015 | 0.46 | 0.03 | EA | 1 | 1 | 3 | 1 | 1110 | 0.80 | 1 |
| Michopoulos 2015 | 2015 | 0.44 | 0.03 | EN | 1 | 1 | 3 | 1 | 1110 | 0.80 | 1 |
| Michopoulos 2015 | 2015 | 0.30 | 0.03 | PA | 1 | 1 | 3 | 1 | 1110 | 0.80 | 1 |
| Michopoulos 2015 | 2015 | 0.37 | 0.03 | PN | 1 | 1 | 3 | 1 | 1110 | 0.80 | 1 |
| Michopoulos 2015 | 2015 | 0.31 | 0.03 | SA | 1 | 1 | 3 | 1 | 1110 | 0.80 | 1 |
| Mikaeili 2013 | 2013 | 0.05 | 0.03 | EA | 0 | 1 | 4 | 5 | 893 | 0.00 | 0 |
| Mikaeili 2013 | 2013 | 0.05 | 0.03 | EN | 0 | 1 | 4 | 5 | 893 | 0.00 | 0 |
| Mikaeili 2013 | 2013 | 0.07 | 0.03 | PA | 0 | 1 | 4 | 5 | 893 | 0.00 | 0 |
| Mikaeili 2013 | 2013 | 0.03 | 0.03 | PN | 0 | 1 | 4 | 5 | 893 | 0.00 | 0 |
| Miller 2014 | 2014 | 0.18 | 0.03 | EA | 1 | 0 | 3 | 1 | 674 | 0.46 | 1 |
| Miller 2014 | 2014 | 0.21 | 0.03 | PA | 1 | 0 | 3 | 1 | 674 | 0.46 | 1 |
| Miller 2014 | 2014 | 0.15 | 0.03 | SA | 1 | 0 | 3 | 1 | 674 | 0.46 | 1 |
| Miller 2017 | 2017 | 0.38 | 0.04 | EA | 0 | 1 | 3 | 1 | 682 |  | 1 |
| Min 2015 | 2015 | 0.46 | 0.03 | EA | 0 | 0 | 3 | 3 | 1198 | 0.67 | 0 |
| Min 2015 | 2015 | 0.34 | 0.03 | PA | 0 | 0 | 3 | 3 | 1198 | 0.67 | 0 |
| Minnich 2017 | 2017 | 0.39 | 0.03 | EN | 0 | 1 | 3 | 1 | 1344 | 0.64 | 1 |
| Minnich 2017 | 2017 | 0.29 | 0.03 | PN | 0 | 1 | 3 | 1 | 1344 | 0.64 | 1 |
| Miron 2014 | 2014 | 0.22 | 0.03 | EA | 1 | 0 | 3 | 1 | 377 | 0.64 | 1 |
| Miron 2014 | 2014 | 0.17 | 0.03 | PA | 1 | 0 | 3 | 1 | 377 | 0.64 | 1 |
| Miron 2014 | 2014 | 0.11 | 0.03 | SA | 1 | 0 | 3 | 1 | 377 | 0.64 | 1 |
| Miron 2016 | 2016 | 0.14 | 0.05 | SA | 0 | 0 | 3 | 1 | 1043 | 1.00 | 1 |
| Mitchell 2005 | 2005 | 0.26 | 0.08 | EA | 1 | 1 | 3 | 1 | 168 | 0.00 | 1 |
| Mitchell 2005 | 2005 | 0.29 | 0.08 | EN | 1 | 1 | 3 | 1 | 168 | 0.00 | 1 |
| Mitchell 2005 | 2005 | 0.35 | 0.08 | PA | 1 | 1 | 3 | 1 | 168 | 0.00 | 1 |
| Mitchell 2005 | 2005 | 0.31 | 0.08 | PN | 1 | 1 | 3 | 1 | 168 | 0.00 | 1 |
| Mitchell 2005 | 2005 | 0.30 | 0.08 | SA | 1 | 1 | 3 | 1 | 168 | 0.00 | 1 |
| Monteiro 2015 | 2015 | 0.46 | 0.06 | EA | 0 | 1 | 3 | 2 | 319 | 0.68 | 0 |
| Monteiro 2015 | 2015 | 0.34 | 0.06 | EN | 0 | 1 | 3 | 2 | 319 | 0.68 | 0 |
| Morokoff 2009 | 2009 | 0.24 | 0.05 | SA | 0 | 0 | 3 | 1 | 473 | 0.66 | 1 |
| Oliver 2003 | 2003 | 0.31 | 0.12 | PA | 0 | 0 | 3 | 1 | 76 | 0.00 | 1 |
| O'Mahen 2015 | 2015 | 0.37 | 0.09 | EA | 1 | 1 | 3 | 1 | 132 | 1.00 | 1 |
| O'Mahen 2015 | 2015 | 0.19 | 0.09 | EN | 1 | 1 | 3 | 1 | 132 | 1.00 | 1 |
| O'Mahen 2015 | 2015 | 0.20 | 0.09 | PA | 1 | 1 | 3 | 1 | 132 | 1.00 | 1 |
| O'Mahen 2015 | 2015 | 0.17 | 0.09 | PN | 1 | 1 | 3 | 1 | 132 | 1.00 | 1 |
| O'Mahen 2015 | 2015 | 0.23 | 0.09 | SA | 1 | 1 | 3 | 1 | 132 | 1.00 | 1 |
| Palosaari 2013 | 2013 | 0.39 | 0.07 | EA | 0 | 0 | 3 | 5 | 197 | 0.50 | 0 |
| Park 2018 | 2018 | 0.07 | 0.02 | PA | 0 | 0 | 4 | 3 | 1796 | 0.48 | 0 |
| Pieritz 2015 | 2015 | 0.50 | 0.13 | EA | 1 | 1 | 3 | 2 | 62 | 1.00 | 0 |
| Pieritz 2015 | 2015 | 0.28 | 0.13 | EN | 1 | 1 | 3 | 2 | 62 | 1.00 | 0 |
| Pieritz 2015 | 2015 | 0.31 | 0.13 | PA | 1 | 1 | 3 | 2 | 62 | 1.00 | 0 |
| Pieritz 2015 | 2015 | 0.29 | 0.13 | PN | 1 | 1 | 3 | 2 | 62 | 1.00 | 0 |
| Pieritz 2015 | 2015 | 0.34 | 0.13 | SA | 1 | 1 | 3 | 2 | 62 | 1.00 | 0 |
| Powers 2009 | 2009 | 0.34 | 0.05 | EA | 0 | 1 | 3 | 1 | 378 | 0.54 | 1 |
| Powers 2009 | 2009 | 0.27 | 0.05 | EN | 0 | 1 | 3 | 1 | 378 | 0.54 | 1 |
| Quevedo 2008 | 2008 | -0.02 | 0.08 | EA | 1 | 0 | 3 | 1 | 170 |  | 1 |
| Quevedo 2008 | 2008 | 0.15 | 0.08 | PA | 1 | 0 | 3 | 1 | 170 |  | 1 |
| Quevedo 2008 | 2008 | 0.30 | 0.08 | SA | 1 | 0 | 3 | 1 | 170 |  | 1 |
| Rabinovitch 2015 | 2015 | 0.18 | 0.08 | SA | 0 | 0 | 3 | 1 | 166 | 1.00 | 1 |
| Raes 2008 | 2008 | 0.38 | 0.10 | EA | 0 | 1 | 3 | 2 | 101 | 0.82 | 0 |
| Randolph 2006 | 2006 | 0.46 | 0.13 | SA | 0 | 0 | 3 | 1 | 63 | 1.00 | 1 |
| Rezaei 2016 | 2016 | 0.50 | 0.05 | EA | 0 | 1 | 3 | 5 | 439 | 1.00 | 0 |
| Rezaei 2016 | 2016 | 0.34 | 0.05 | EN | 0 | 1 | 3 | 5 | 439 | 1.00 | 0 |
| Rezaei 2016 | 2016 | 0.23 | 0.05 | PA | 0 | 1 | 3 | 5 | 439 | 1.00 | 0 |
| Rezaei 2016 | 2016 | 0.31 | 0.05 | PN | 0 | 1 | 3 | 5 | 439 | 1.00 | 0 |
| Rich 2005 | 2005 | 0.22 | 0.03 | EA | 1 | 0 | 3 | 1 | 551 | 1.00 | 1 |
| Rich 2005 | 2005 | 0.23 | 0.03 | PA | 1 | 0 | 3 | 1 | 551 | 1.00 | 1 |
| Rich 2005 | 2005 | 0.19 | 0.03 | SA | 1 | 0 | 3 | 1 | 551 | 1.00 | 1 |
| Riggs 2010 | 2010 | 0.04 | 0.06 | EA | 1 | 1 | 3 | 1 | 285 | 0.77 | 1 |
| Riggs 2010 | 2010 | 0.05 | 0.06 | PA | 1 | 1 | 3 | 1 | 285 | 0.77 | 1 |
| Riggs 2010 | 2010 | 0.12 | 0.06 | SA | 1 | 1 | 3 | 1 | 285 | 0.77 | 1 |
| Roatta 2000 | 2000 | 0.30 | 0.11 | SA | 0 | 0 | 3 | 1 | 92 | 1.00 | 1 |
| Roosa 1999 | 1999 | 0.27 | 0.03 | SA | 0 | 0 |  | 1 | 2003 | 1.00 | 1 |
| Salah 2015 | 2015 | -0.09 | 0.23 | EA | 1 | 1 | 3 | 2 | 22 | 0.95 | 0 |
| Salah 2015 | 2015 | 0.34 | 0.23 | EN | 1 | 1 | 3 | 2 | 22 | 0.95 | 0 |
| Salah 2015 | 2015 | 0.42 | 0.23 | PA | 1 | 1 | 3 | 2 | 22 | 0.95 | 0 |
| Salah 2015 | 2015 | 0.52 | 0.23 | PN | 1 | 1 | 3 | 2 | 22 | 0.95 | 0 |
| Salah 2015 | 2015 | -0.05 | 0.23 | SA | 1 | 1 | 3 | 2 | 22 | 0.95 | 0 |
| Schulz, Schmidt 2014 | 2014 | 0.18 | 0.02 | EA | 1 | 1 | 4 | 2 | 2265 | 0.53 | 0 |
| Schulz, Schmidt 2014 | 2014 | 0.15 | 0.02 | EN | 1 | 1 | 4 | 2 | 2265 | 0.53 | 0 |
| Schulz, Schmidt 2014 | 2014 | 0.11 | 0.02 | PA | 1 | 1 | 4 | 2 | 2265 | 0.53 | 0 |
| Schulz, Schmidt 2014 | 2014 | 0.10 | 0.02 | SA | 1 | 1 | 4 | 2 | 2265 | 0.53 | 0 |
| Schumm 2005 | 2005 | 0.23 | 0.08 | PA | 0 | 1 | 3 | 1 | 176 | 1.00 | 1 |
| Schumm 2005 | 2005 | 0.31 | 0.08 | SA | 0 | 1 | 3 | 1 | 176 | 1.00 | 1 |
| Seok 2012 | 2012 | 0.65 | 0.21 | EA | 1 | 0 | 1 | 3 | 52 | 0.73 | 0 |
| Seok 2012 | 2012 | 0.44 | 0.21 | PA | 1 | 0 | 1 | 3 | 52 | 0.73 | 0 |
| Seok 2012 | 2012 | 0.04 | 0.21 | SA | 1 | 0 | 1 | 3 | 52 | 0.73 | 0 |
| Shahar 2015 | 2015 | 0.34 | 0.07 | EA | 0 | 1 | 3 | 2 | 219 | 0.50 | 0 |
| Shahar 2015 | 2015 | 0.27 | 0.07 | EN | 0 | 1 | 3 | 2 | 219 | 0.50 | 0 |
| Shao 2020 | 2020 | 0.11 | 0.04 | EA | 1 | 1 | 3 | 3 | 718 | 0.51 | 0 |
| Shao 2020 | 2020 | 0.11 | 0.04 | EN | 1 | 1 | 3 | 3 | 718 | 0.51 | 0 |
| Shao 2020 | 2020 | 0.04 | 0.04 | PA | 1 | 1 | 3 | 3 | 718 | 0.51 | 0 |
| Shao 2020 | 2020 | 0.09 | 0.04 | PN | 1 | 1 | 3 | 3 | 718 | 0.51 | 0 |
| Shao 2020 | 2020 | 0.01 | 0.04 | SA | 1 | 1 | 3 | 3 | 718 | 0.51 | 0 |
| Shapero 2013 | 2013 | 0.76 | 0.08 | EA | 0 | 1 | 3 | 1 | 216 | 0.58 | 1 |
| Shi 2013 | 2013 | 0.56 | 0.04 | EA | 1 | 1 | 2 | 1 | 497 | 0.65 | 1 |
| Shi 2013 | 2013 | 0.14 | 0.04 | EN | 1 | 1 | 2 | 1 | 497 | 0.65 | 1 |
| Shi 2013 | 2013 | -0.26 | 0.04 | PA | 1 | 1 | 2 | 1 | 497 | 0.65 | 1 |
| Shi 2013 | 2013 | -0.01 | 0.04 | PN | 1 | 1 | 2 | 1 | 497 | 0.65 | 1 |
| Shi 2013 | 2013 | 0.08 | 0.04 | SA | 1 | 1 | 2 | 1 | 497 | 0.65 | 1 |
| Song 2016 | 2016 | 0.27 | 0.06 | EA | 1 | 1 | 2 | 3 | 305 | 0.57 | 0 |
| Song 2016 | 2016 | 0.28 | 0.06 | EN | 1 | 1 | 2 | 3 | 305 | 0.57 | 0 |
| Song 2016 | 2016 | 0.21 | 0.06 | PA | 1 | 1 | 2 | 3 | 305 | 0.57 | 0 |
| Song 2016 | 2016 | 0.17 | 0.06 | PN | 1 | 1 | 2 | 3 | 305 | 0.57 | 0 |
| Song 2016 | 2016 | 0.20 | 0.06 | SA | 1 | 1 | 2 | 3 | 305 | 0.57 | 0 |
| Song 2020 | 2020 | 0.39 | 0.01 | EA | 1 | 1 | 3 | 3 | 7643 | 0.66 | 0 |
| Song 2020 | 2020 | 0.37 | 0.01 | EN | 1 | 1 | 3 | 3 | 7643 | 0.66 | 0 |
| Song 2020 | 2020 | 0.21 | 0.01 | PA | 1 | 1 | 3 | 3 | 7643 | 0.66 | 0 |
| Song 2020 | 2020 | 0.31 | 0.01 | PN | 1 | 1 | 3 | 3 | 7643 | 0.66 | 0 |
| Song 2020 | 2020 | 0.19 | 0.01 | SA | 1 | 1 | 3 | 3 | 7643 | 0.66 | 0 |
| Spertus 2003 | 2003 | 0.40 | 0.07 | EA | 1 | 1 | 3 | 1 | 205 | 1.00 | 1 |
| Spertus 2003 | 2003 | 0.31 | 0.07 | EN | 1 | 1 | 3 | 1 | 205 | 1.00 | 1 |
| Spertus 2003 | 2003 | 0.15 | 0.07 | PA | 1 | 1 | 3 | 1 | 205 | 1.00 | 1 |
| Spertus 2003 | 2003 | 0.18 | 0.07 | SA | 1 | 1 | 3 | 1 | 205 | 1.00 | 1 |
| Spinhoven 2014 | 2014 | 0.37 | 0.02 | EA | 1 | 1 | 2 | 2 | 2308 | 0.66 | 0 |
| Spinhoven 2014 | 2014 | 0.39 | 0.02 | EN | 1 | 1 | 2 | 2 | 2308 | 0.66 | 0 |
| Spinhoven 2014 | 2014 | 0.23 | 0.02 | PA | 1 | 1 | 2 | 2 | 2308 | 0.66 | 0 |
| Spinhoven 2014 | 2014 | 0.28 | 0.02 | PN | 1 | 1 | 2 | 2 | 2308 | 0.66 | 0 |
| Spinhoven 2014 | 2014 | 0.13 | 0.02 | SA | 1 | 1 | 2 | 2 | 2308 | 0.66 | 0 |
| Stange 2014f | 2014 | 0.62 | 0.09 | EA | 0 | 1 | 3 | 1 | 138 | 1.00 | 1 |
| Stange 2014f | 2014 | 0.50 | 0.09 | EN | 0 | 1 | 3 | 1 | 138 | 1.00 | 1 |
| Stange 2014m | 2014 | 0.47 | 0.09 | EA | 0 | 1 | 3 | 1 | 119 | 0.00 | 1 |
| Stange 2014m | 2014 | 0.42 | 0.09 | EN | 0 | 1 | 3 | 1 | 119 | 0.00 | 1 |
| Steffey 2012 | 2012 | 0.42 | 0.07 | EA | 1 | 1 | 3 | 1 | 207 | 0.73 | 1 |
| Steffey 2012 | 2012 | 0.50 | 0.07 | EN | 1 | 1 | 3 | 1 | 207 | 0.73 | 1 |
| Steffey 2012 | 2012 | 0.22 | 0.07 | PA | 1 | 1 | 3 | 1 | 207 | 0.73 | 1 |
| Steffey 2012 | 2012 | 0.48 | 0.07 | PN | 1 | 1 | 3 | 1 | 207 | 0.73 | 1 |
| Steffey 2012 | 2012 | 0.32 | 0.07 | SA | 1 | 1 | 3 | 1 | 207 | 0.73 | 1 |
| Stewart 2015 | 2015 | -0.09 | 0.08 | SA | 0 | 1 | 2 | 1 | 163 | 0.77 | 1 |
| Tanaka 2011 | 2011 | 0.22 | 0.09 | EA | 1 | 1 | 3 | 1 | 117 | 0.55 | 1 |
| Tanaka 2011 | 2011 | 0.28 | 0.09 | EN | 1 | 1 | 3 | 1 | 117 | 0.55 | 1 |
| Tanaka 2011 | 2011 | 0.21 | 0.09 | PA | 1 | 1 | 3 | 1 | 117 | 0.55 | 1 |
| Tanaka 2011 | 2011 | 0.30 | 0.09 | PN | 1 | 1 | 3 | 1 | 117 | 0.55 | 1 |
| Tanaka 2011 | 2011 | 0.21 | 0.09 | SA | 1 | 1 | 3 | 1 | 117 | 0.55 | 1 |
| Tang 2018 | 2018 | 0.42 | 0.02 | EA | 0 | 1 | 2 | 3 | 5505 | 0.54 | 0 |
| Tang 2018 | 2018 | 0.12 | 0.02 | EN | 0 | 1 | 2 | 3 | 5505 | 0.54 | 0 |
| Tang 2018 | 2018 | 0.19 | 0.01 | PA | 0 | 1 | 2 | 3 | 5505 | 0.54 | 0 |
| Tang 2018 | 2018 | 0.13 | 0.01 | PN | 0 | 1 | 2 | 3 | 5505 | 0.54 | 0 |
| Thomas 2011 | 2011 | 0.38 | 0.10 | SA | 0 | 0 | 3 | 1 | 110 | 1.00 | 1 |
| Tlapek 2017 | 2017 | 0.40 | 0.07 | EA | 1 | 1 | 3 | 1 | 237 | 1.00 | 1 |
| Tlapek 2017 | 2017 | 0.14 | 0.07 | PA | 1 | 1 | 3 | 1 | 237 | 1.00 | 1 |
| Tlapek 2017 | 2017 | 0.24 | 0.07 | SA | 1 | 1 | 3 | 1 | 237 | 1.00 | 1 |
| Turner 2004 | 2004 | 0.14 | 0.04 | PA | 0 | 0 | 3 | 1 | 649 | 0.59 | 1 |
| Ullman 2014 | 2014 | 0.24 | 0.02 | SA | 0 | 0 | 3 | 1 | 1863 | 1.00 | 1 |
| Van Vugt 2014 | 2014 | 0.68 | 0.11 | EA | 1 | 1 | 2 | 1 | 89 | 1.00 | 1 |
| Van Vugt 2014 | 2014 | 0.42 | 0.11 | EN | 1 | 1 | 2 | 1 | 89 | 1.00 | 1 |
| Van Vugt 2014 | 2014 | 0.51 | 0.11 | PA | 1 | 1 | 2 | 1 | 89 | 1.00 | 1 |
| Van Vugt 2014 | 2014 | 0.32 | 0.11 | PN | 1 | 1 | 2 | 1 | 89 | 1.00 | 1 |
| Van Vugt 2014 | 2014 | 0.40 | 0.11 | SA | 1 | 1 | 2 | 1 | 89 | 1.00 | 1 |
| Virkler 2006 | 2006 | 0.32 | 0.12 | SA | 0 | 1 | 3 | 1 | 75 | 1.00 | 1 |
| Voth Schrag 2017 | 2017 | 0.34 | 0.10 | EA | 1 | 1 | 2 | 1 | 105 | 1.00 | 1 |
| Voth Schrag 2017 | 2017 | 0.16 | 0.10 | PA | 1 | 1 | 2 | 1 | 105 | 1.00 | 1 |
| Voth Schrag 2017 | 2017 | 0.10 | 0.10 | PN | 1 | 1 | 2 | 1 | 105 | 1.00 | 1 |
| Voth Schrag 2017 | 2017 | 0.22 | 0.10 | SA | 1 | 1 | 2 | 1 | 105 | 1.00 | 1 |
| Wang, Liu 2015 | 2015 | 0.33 | 0.05 | EA | 1 | 1 | 3 | 3 | 475 | 0.51 | 0 |
| Wang, Liu 2015 | 2015 | 0.38 | 0.05 | EN | 1 | 1 | 3 | 3 | 475 | 0.51 | 0 |
| Wang, Liu 2015 | 2015 | 0.46 | 0.06 | PA | 1 | 1 | 3 | 3 | 475 | 0.51 | 0 |
| Wang, Liu 2015 | 2015 | 0.41 | 0.05 | PN | 1 | 1 | 3 | 3 | 475 | 0.51 | 0 |
| Wang, Liu 2015 | 2015 | 0.34 | 0.05 | SA | 1 | 1 | 3 | 3 | 475 | 0.51 | 0 |
| Wanklyn 2012 | 2012 | 0.50 | 0.10 | EA | 1 | 1 | 3 | 1 | 110 | 0.39 | 1 |
| Wanklyn 2012 | 2012 | 0.32 | 0.10 | EN | 1 | 1 | 3 | 1 | 110 | 0.39 | 1 |
| Wanklyn 2012 | 2012 | 0.32 | 0.10 | PA | 1 | 1 | 3 | 1 | 110 | 0.39 | 1 |
| Wanklyn 2012 | 2012 | 0.33 | 0.10 | PN | 1 | 1 | 3 | 1 | 110 | 0.39 | 1 |
| Wanklyn 2012 | 2012 | 0.52 | 0.10 | SA | 1 | 1 | 3 | 1 | 110 | 0.39 | 1 |
| Weissmann 1992 | 1992 | 0.17 | 0.12 | PA | 0 | 0 | 3 | 1 | 259 | 1.00 | 1 |
| Whiffen 1999 | 1999 | 0.06 | 0.13 | SA | 0 | 0 | 3 | 1 | 60 | 1.00 | 1 |
| Whiffen 2000 | 2000 | 0.37 | 0.07 | SA | 0 | 0 | 3 | 1 | 192 | 0.57 | 1 |
| Woods 2010 | 2010 | 0.27 | 0.08 | EA | 1 | 1 | 3 | 1 | 157 | 1.00 | 1 |
| Woods 2010 | 2010 | 0.04 | 0.08 | EN | 1 | 1 | 3 | 1 | 157 | 1.00 | 1 |
| Woods 2010 | 2010 | 0.14 | 0.08 | PA | 1 | 1 | 3 | 1 | 157 | 1.00 | 1 |
| Woods 2010 | 2010 | 0.11 | 0.08 | PN | 1 | 1 | 3 | 1 | 157 | 1.00 | 1 |
| Woods 2010 | 2010 | 0.24 | 0.08 | SA | 1 | 1 | 3 | 1 | 157 | 1.00 | 1 |
| Wu 2018 | 2018 | 0.22 | 0.05 | EA | 1 | 1 | 3 | 3 | 358 | 0.63 | 0 |
| Wu 2018 | 2018 | 0.29 | 0.05 | EN | 1 | 1 | 3 | 3 | 358 | 0.63 | 0 |
| Wu 2018 | 2018 | 0.11 | 0.05 | PA | 1 | 1 | 3 | 3 | 358 | 0.63 | 0 |
| Wu 2018 | 2018 | 0.21 | 0.05 | PN | 1 | 1 | 3 | 3 | 358 | 0.63 | 0 |
| Wu 2018 | 2018 | 0.14 | 0.05 | SA | 1 | 1 | 3 | 3 | 358 | 0.63 | 0 |
| Wuest 2010 | 2010 | 0.37 | 0.06 | EA | 1 | 1 | 3 | 1 | 309 | 1.00 | 1 |
| Wuest 2010 | 2010 | 0.27 | 0.06 | PA | 1 | 1 | 3 | 1 | 309 | 1.00 | 1 |
| Wuest 2010 | 2010 | 0.20 | 0.06 | SA | 1 | 1 | 3 | 1 | 309 | 1.00 | 1 |
| Zalewski 2013 | 2013 | 0.55 | 0.10 | EA | 1 | 1 | 2 | 1 | 95 | 1.00 | 1 |
| Zalewski 2013 | 2013 | 0.47 | 0.10 | EN | 1 | 1 | 2 | 1 | 95 | 1.00 | 1 |
| Zalewski 2013 | 2013 | 0.38 | 0.10 | PA | 1 | 1 | 2 | 1 | 95 | 1.00 | 1 |
| Zalewski 2013 | 2013 | 0.40 | 0.10 | PN | 1 | 1 | 2 | 1 | 95 | 1.00 | 1 |
| Zalewski 2013 | 2013 | 0.55 | 0.10 | SA | 1 | 1 | 2 | 1 | 95 | 1.00 | 1 |
| Zhang 2020 | 2020 | 0.38 | 0.04 | EA | 1 | 1 | 2 | 3 | 1040 | 1.00 | 0 |
| Zhang 2020 | 2020 | 0.31 | 0.03 | EN | 1 | 1 | 2 | 3 | 1040 | 1.00 | 0 |
| Zhang 2020 | 2020 | 0.24 | 0.03 | PA | 1 | 1 | 2 | 3 | 1040 | 1.00 | 0 |
| Zhang 2020 | 2020 | 0.34 | 0.03 | PN | 1 | 1 | 2 | 3 | 1040 | 1.00 | 0 |
| Zhang 2020 | 2020 | 0.27 | 0.03 | SA | 1 | 1 | 2 | 3 | 1040 | 1.00 | 0 |
| Zhang 2022 | 2022 | 0.59 | 0.01 | EA | 1 | 1 | 3 | 3 | 6228 | 0.52 | 0 |
| Zhang 2022 | 2022 | 0.54 | 0.01 | EN | 1 | 1 | 3 | 3 | 6228 | 0.52 | 0 |
| Zhang 2022 | 2022 | 0.30 | 0.01 | PA | 1 | 1 | 3 | 3 | 6228 | 0.52 | 0 |
| Zhang 2022 | 2022 | 0.20 | 0.01 | PN | 1 | 1 | 3 | 3 | 6228 | 0.52 | 0 |
| Zhang 2022 | 2022 | 0.18 | 0.01 | SA | 1 | 1 | 3 | 3 | 6228 | 0.52 | 0 |
| Zhou 2019 | 2019 | 0.31 | 0.06 | EA | 1 | 3 | 1 | 3 | 312 | 0.58 | 0 |
| Zhou 2019 | 2019 | 0.39 | 0.07 | EN | 1 | 3 | 1 | 3 | 312 | 0.58 | 0 |
| Zhou 2019 | 2019 | -0.10 | 0.06 | PA | 1 | 3 | 1 | 3 | 312 | 0.58 | 0 |
| Zhou 2019 | 2019 | 0.30 | 0.06 | PN | 1 | 3 | 1 | 3 | 312 | 0.58 | 0 |
| Zhou 2019 | 2019 | -0.06 | 0.06 | SA | 1 | 3 | 1 | 3 | 312 | 0.58 | 0 |
